# Efficacy of home treatment and inpatient treatment for children and adolescents in psychiatric crisis: a systematic review and meta-analysis 

**DOI:** 10.1007/s00787-026-03060-0

**Published:** 2026-06-01

**Authors:** Karolina Foremnik, Gaby Sroczynski, Jan Stratil, Marjan Arvandi, Anja Neumann, Barbara Buchberger

**Affiliations:** 1 Medical Faculty, University of Duisburg-Essen, Essen, Germany; 2 UMIT TIROL - University for Health Sciences and Technology, Hall in Tirol, Austria; 3 Robert Koch Institute, Berlin, Germany

**Keywords:** Home treatment, Inpatient treatment, Psychiatric crisis, Children, Adolescents, Meta-analysis

## Abstract

Inpatient treatment is widely used for children and adolescents in psychiatric crisis but may disrupt family life and routines. Home treatment offers a less restrictive alternative. This systematic review and meta-analysis compared both approaches regarding psychopathology, family functioning, psychosocial functioning, and readmissions, considering post-treatment vs. follow-up effects and sequential vs. stand-alone models. Following PRISMA 2020, systematic searches were conducted in Medline, Cochrane Library, Embase, PsycInfo, and trial registries. Two reviewers independently screened articles, extracted data, and assessed risk of bias. Random-effects meta-analyses were conducted where feasible, and certainty of evidence was rated using Grading of Recommendations Assessment, Development and Evaluation (GRADE). The protocol was registered in PROSPERO (CRD42023458888). Fifteen studies (*N* = 617 participants) were included, with eleven contributing to meta-analyses. No significant differences were found between HT and IT for psychopathology, psychosocial functioning, or readmissions. A significant subgroup difference between post-treatment and follow-up was observed for psychosocial functioning (*p* = 0.0058), with inpatient treatment showing short-term advantages and HT more favorable outcomes at follow-up. Effects on psychopathology were small and non-significant, and readmission rates did not differ (risk ratio (RR) = 1.27, 95% CI [0.93–1.74]). Evidence on sequential models and family functioning was limited and showed no clear superiority of either approach. HT appears comparable to IT and may offer more sustained improvements in psychosocial functioning. Due to the limited quality of the included studies, these results should be interpreted with caution.

**Supplementary Information:**

The online version contains supplementary material available at 10.1007/s00787-026-03060-0.

## Introduction

Approximately 14% of children and adolescents aged 10–19 years globally experience a mental disorder [1], with depression, anxiety, and behavioral disorders among the leading causes of illness [1]. Suicide is the third leading cause of death among adolescents and young adults aged 15–29 years globally [1]. During the Coronavirus Disease 2019 (COVID-19) pandemic, psychiatric emergencies among adolescents increased substantially, with school closures, disrupted routines, and reduced social contact contributing to increased mental distress [2].

Despite the substantial burden, access to inpatient and outpatient child and adolescent psychiatric services remains inadequate worldwide [1, 3], which is particularly concerning given the high risk of chronicity, as mental health problems in childhood and adolescence often persist into adulthood [4]. Structural barriers-including insufficient service capacity, long waiting times, workforce shortages, and financial constraints- continue to impede timely access to care, while stigma and negative attitudes towards mental health treatment may further discourage help-seeking [3]. Internationally comparable and standardized indicators of access to inpatient psychiatric treatment, such as waiting times, remain scarce [5]. Nevertheless, available reports from high-income countries suggest considerable structural strain within acute psychiatric services. For example, due to capacity constraints, children and adolescents may be admitted far from their home region [6] or placed in adult psychiatric wards, which often lack age-appropriate therapeutic, educational, and social environments [7].

Inpatient treatment (IT) is traditionally considered the standard response to severe psychiatric crises in children and adolescents. Although inpatient admission may be unavoidable in situations involving acute risk of harm to self or others, it primarily focuses on symptom stabilization and requires temporary separation from the family and everyday environment [8, 9]. Yet psychiatric crises in children and adolescents are frequently embedded in family and social contexts, which inpatient treatment can only address to a limited extent [10, 11]. Moreover, the highly structured hospital setting may limit the transferability of treatment gains to the often more complex and potentially dysfunctional home environment following discharge [8].

Although there is no universally accepted definition of a psychiatric crisis [12–16], the term is used here to describe acute phases of severe psychological distress requiring immediate clinical intervention, which may extend beyond initial stabilization and include short- to medium-term intensive treatment [17–19]. This focus reflects the clinical indication for treatment rather than the presence of a particular disorder and distinguishes crisis-oriented care from longer-term treatment approaches (e.g., residential treatment) for chronic mental health conditions. In some cases, such crises may escalate into a psychiatric emergency characterized by an immediate risk to the life or health of the individual or others. The transition between psychiatric crisis and emergency is therefore often gradual, with fluid boundaries between both states [19].

Capacity constraints as well as conceptual and clinical considerations have led to the development of home treatment (HT) as an alternative model for managing psychiatric crises in children and adolescents within the patient’s home environment [20]. HT is commonly used as an umbrella term for a heterogeneous group of outreach-based interventions aimed at reducing or avoiding inpatient admissions while enabling participation in everyday life within the familiar social and family environment [20, 21]. In this context, HT refers to outreach-based treatment delivered at home as a clinically viable alternative to inpatient admission [20]. Programs may range from flexible, non-manualized home-based crisis services to structured, manualized outreach models such as Multisystemic Therapy (MST) [21–23].

Evidence on the effectiveness of inpatient and home treatment in children and adolescents remains limited. While inpatient treatment may facilitate short-term stabilization, findings on sustained improvement after discharge are inconsistent [24, 25]. Existing reviews suggest that home treatment represents a viable alternative to inpatient treatment without demonstrating clear superiority, but are characterized by substantial heterogeneity in service models and overall limited evidence quality [20, 21]. Randomized controlled trials remain scarce, partly due to ethical and practical challenges in acute settings [26]. A recent meta-analysis by Graf et al. (2024) found home treatment to be non-inferior to inpatient treatment across psychosocial functioning, psychopathology, and readmission rates. However, heterogeneity and limited comparability of included models remain important limitations [27].

In contrast to previous meta-analyses, the present study applies stricter conceptual criteria to define both home and inpatient treatment, focusing exclusively on interventions representing direct alternatives in acute psychiatric crises. This allows for improved comparability of treatment models and a more precise estimation of treatment effects. Specifically, we distinguish between home treatment interventions delivered entirely in the patient’s home environment (here referred to as stand-alone models) and those involving an initial inpatient stabilization followed by home-based continuation of care (sequential models), as these represent conceptually distinct care pathways. Furthermore, we restrict the analysis to acute inpatient psychiatric treatment with continuous 24-hour supervision and to home treatment models representing a direct alternative to hospital admission. This approach aims to reduce heterogeneity by excluding settings primarily oriented toward longer-term psychosocial rehabilitation (e.g., residential care), which differ substantially in structure and intensity. In addition, we examine potential time-dependent effects by comparing short-term and follow-up outcomes under more comparable conceptual conditions. From a clinical perspective, inpatient treatment may facilitate rapid symptom stabilization, whereas home treatment may promote more sustained functional adaptation by embedding therapeutic processes within the family and social context [20, 28]. Finally, we adopt a more differentiated approach to outcome assessment by considering clinically meaningful domains (e.g., internalizing and externalizing symptoms, as well as different aspects of psychosocial functioning), as aggregated outcomes may obscure relevant differences between treatment settings.

The present meta-analysis includes only studies that directly compare HT with IT. By combining a structural differentiation of treatment models with a time-sensitive and domain-specific analytic approach, this review aims to provide a more refined understanding of the comparative effectiveness of these interventions in children and adolescents experiencing psychiatric crises.

## Methods

This systematic review was conducted in accordance with the Preferred Reporting Items for Systematic Reviews and Meta-Analyses (PRISMA) guidelines [29] (see Online Resource 1). All relevant data, including the PRISMA checklist, have been stored within the Open Science Framework (OSF) and can be accessed via the following link: https://osf.io/sz4fh/?view_only=ddf867083de5400cbd0311853f85536c.

The protocol for this systematic review was prospectively registered with the International Prospective Register of Systematic Reviews (PROSPERO; registration number CRD42023458888) and submitted to PLOS ONE along with the manuscript (Manuscript number: PONE-D-24-45550R1; see Online Resource 2 for the full version of the study protocol). ChatGPT assisted with R-script drafting; all analyses and interpretations were independently conducted and fully assumed by the first author.

### Information sources

A systematic search was conducted in the bibliographic databases Medline, Cochrane Library, Embase, and PsycInfo. To ensure comprehensive coverage and minimize publication bias, grey literature was explored through sources such as Google Scholar and the Education Resources Information Center (ERIC), with the first 50 results screened (although OpenGrey was originally listed in the review protocol, it was excluded due to its discontinuation). Additional sources included hand searches and reference list screenings. Ongoing and unpublished trials were identified via ClinicalTrials.gov and the WHO International Clinical Trials Registry Platform (ICTRP). Where necessary, study authors and relevant organizations were contacted for unpublished data, clarification or raw data. In total, three authors were contacted. All searches were conducted from database inception to May 2024. An updated literature search was conducted in the main bibliographic databases to identify studies published between 01.04.2024 and 04.08.2025.

## Eligibility criteria

Only quantitative primary studies with a comparative design were considered. Eligibility criteria were structured according to the PICO framework. Complete inclusion and exclusion criteria are provided in the study protocol (see Online Resource 2).

## Population

Children and adolescents under the age of 18 living with stable, non-institutional caregivers were included. No restrictions were applied regarding socioeconomic or cultural background. Participants had to present with severe mental disorders with marked functional impairment (first episode or as an acute exacerbation); comorbidities were not exclusionary. Studies addressing acute mental health crises such as suicidality, self-harm, mood or anxiety disorders, psychosis, agitation, or trauma-related symptoms were eligible. Exclusion criteria included chronic psychiatric courses requiring intensive treatment, neurodevelopmental disorders (e.g., autism), and severe somatic conditions (e.g., intoxication, anorexia-related decompensation). Full eligibility criteria, including ICD-10 codes, are detailed in the protocol (see Online Resource 2).

## Intervention

Only HT interventions designed as direct alternatives to IT were included. Eligible interventions were delivered in non-clinical settings (e.g., home, school), involved multidisciplinary teams, ensured round-the-clock crisis support. Programs primarily designed as single-provider models, institutional programs (e.g., group homes), preventive services, and community-based interventions (e.g. Assertive Community Treatment [ACT]), primarily targeting rehabilitation in the context of chronic mental health conditions were excluded. Interventions could range from short-term crisis stabilization to more extended treatment phases. Similarly, inpatient treatment could encompass both acute crisis stabilization and subsequent short- to medium-term treatment phases.

## Comparator

The comparator was standard inpatient psychiatric treatment, defined as continuous (24/7) supervision and multidisciplinary treatment in hospital-based settings. Studies with comparison groups located in emergency departments or general somatic hospital wards without structured psychiatric treatment were excluded. Analogous to the intervention criteria, institutional treatment formats such as residential treatment models were also excluded, as these programs typically represent longer-term therapeutic placements aimed at psychosocial rehabilitation.

### Outcomes

The review focused on studies reporting between-group differences (intervention vs. comparator) in at least one of the following outcomes: psychosocial functioning, psychopathology, or family functioning (assessed by children and adolescents, parents, teachers or clinicians) and readmission rates.

## Search strategy

A highly sensitive search strategy was developed and piloted by the review team for each database (see Online Resource 3). To ensure maximum sensitivity, the search was limited to terms related to Population, Intervention, and Comparator, intentionally omitting outcome-specific keywords listed under the eligibility criteria. No date restrictions were applied. Only studies published in English or German were included, using language filters in the databases; no additional search filters were applied. To enhance comprehensiveness, backward citation tracking was performed for all included studies. The update search applied the same search strategy and eligibility criteria.

## Data management and study selection

The search results were managed using the web application Rayyan, which was also used for deduplication. Relevant studies were selected stepwise using the predefined inclusion and exclusion criteria. Title/abstract screening and full-text screening were conducted in Rayyan. The screening and selection process was conducted using a double-review procedure at each stage. All screening steps were performed by KF and independently reviewed by a second reviewer (title and abstract screening by CR, full-text screening by NB). Discrepancies were resolved through discussion or consultation with a third reviewer (BB).The results of the inclusion and exclusion process for all studies were presented in a PRISMA flowchart that illustrates the entire screening and selection process [30]. Additionally, reasons for exclusion at the full-text screening stage and for the quantitative synthesis were documented (see Online Resource 4).

### Data items and data extraction

Data from eligible full texts were manually extracted using predefined, structured Excel tables. To ensure accuracy and reduce bias, all data were independently verified by a second reviewer [31]; discrepancies were resolved through discussion and consensus. No automation tools were used. In cases of missing or unclear data, study authors were contacted. No assumptions were made beyond the information explicitly stated in the articles.

The extracted data covered:

1. Study characteristics such as author, publication year, country, sample size, age, clinical presentation, outcome measures, and assessment time points.
2. Intervention characteristics: For HT interventions- intervention type, setting, duration, frequency, team composition, and treatment modes. Information on IT characteristics was extracted from each study, including intervention type, duration, team composition, and treatment modes. Extracted intervention characteristics are summarized in the results section (under “home treatment interventions” and “comparison interventions”) and reported in detail in the Supplementary Material (see Online Resource 7).
3. For continuous outcomes (psychosocial functioning, psychopathology, and family functioning), data were extracted to calculate standardized mean differences (SMDs; Hedges’ g), including effect direction, sample sizes, means, and standard deviations for intervention and comparison groups at each relevant assessment point.
4. Data on readmission (readmission probabilities, follow-up duration, sample size). Readmissions were defined as any psychiatric rehospitalization after discharge.

### Study risk of bias assessment

The Cochrane RoB 2 tool was applied to assess the risk of bias in randomized controlled trials, while the ROBINS-I tool was used for non-randomized intervention studies [32, 33]. Risk of bias was assessed independently by two reviewers (KF and NB). Disagreements were resolved through discussion or, if necessary, by consultation with a third reviewer (BB) to reach consensus. The results of the assessment were presented in structured risk-of-bias tables. Judgements were reported as low risk, some concerns, or high risk (RoB 2), and as low, moderate, serious, or critical risk of bias (ROBINS-I), in accordance with the criteria of the respective tools. Visual summaries of risk-of-bias assessments were created using the robvis tool [34].

### Effect measures

To compare pre-post differences between intervention and groups across different outcome measures, standardized mean differences (hedges’g) for psychopathology, psychosocial functioning and family functioning based on change scores, using the method proposed by Morris (2008) were calculated [35, 36]. Change scores were computed from pre- and post-means, assuming a pre-post correlation of *r* = 0.5 [37]. Calculations were conducted manually in R-Studio Version 2024.12.0 + 467. Standard error was calculated using the formula by Borenstein et al. [38, 39].

For secondary outcomes, the probability of readmissions, the sample size, as well as the follow-up duration for each study arm were extracted. The analysis was performed on the logarithmic scale: the natural logarithm of the RR (ln[RR]) was calculated for each study, pooled across studies, and subsequently back-transformed to the RR scale for interpretation [37, 40]. Standard errors were derived on the ln(RR) scale [41]. 

### Synthesis methods

All included studies were tabulated by key characteristics and baseline/follow-up data (means, standard deviations, sample sizes) to calculate Hedges’ g (see Online Resource 5). Continuous outcomes were classified by rater (clinician, parent, self, teacher) and assigned to domains: Psychopathology (general, internalizing, externalizing), Psychosocial functioning (overall, social, school/work, substance use), and Family functioning (cohesion, adaptability, control, general). Continuous outcomes pooled in the meta-analyses included measures of psychopathology and psychosocial functioning, while readmission rates were synthesized using pooled risk ratios. Outcomes related to family functioning and sequential treatment models were not pooled due to the limited number of studies and were therefore summarized narratively.

Missing statistical information required for effect size calculation (e.g., group-specific means, standard deviations or readmission rates) was identified in three studies (Ougrin et al., 2021; Graf et al., 2025; Boege et al., 2021). Additional information for readmission rates was provided by Graf et al. (2025) and Ougrin et al. (2021) and incorporated into the quantitative synthesis, whereas the requested data for the remaining studies were not available.

To ensure statistical independence, only one effect size per study and outcome domain was included [42]. When multiple measures were available within a domain, pre-defined selection criteria were applied. First, a rater hierarchy was used (clinician > parent > self). Second, outcome prioritization within domains was applied (for psychopathology: general > internalizing > externalizing; for psychosocial functioning: overall > social > school/work > risk). For follow-up analyses, the shortest available follow-up interval was selected. If, after applying these criteria, multiple outcomes within the same domain remained, a single pooled effect size (Hedges’ g) was calculated assuming a correlation of *r* = 0.5 between measures [43]. Aggregation was not performed when outcomes were conceptually distinct or derived from different rater perspectives. For example, self-reported and clinician-rated outcomes were not combined, and outcomes reflecting different constructs within the same study (e.g., mood symptoms vs. self-harm) were treated separately to avoid combining heterogeneous constructs.

Effect sizes were Hedges’ g with 95% CIs for continuous outcomes and pooled risk ratios based on ln(RR) for readmissions [37, 40]. Separate syntheses were conducted for sequential and stand-alone home treatment models. Differences between post-treatment and follow-up effects were examined within a single meta-analytic model using subgroup analyses.

Despite the limited number of studies, we opted for a quantitative synthesis to provide a more systematic, transparent and reproducible estimate of effect direction compared to a narrative summary. Where data were too limited, results were not pooled but summarized narratively. To account for the small sample sizes and anticipated clinical heterogeneity, we employed random-effects meta-analyses using Hartung- Knapp (HK) confidence intervals with the Paule- Mandel (PM) estimator, as recommended by the Cochrane Working Group for meta-analyses with few studies [44]. Where applicable, an ad hoc variance correction was applied [45]. Pooling was conducted when at least two independent comparisons were available (k ≥ 2; k = number of independent study populations) and the measures were conceptually compatible. Otherwise, a narrative synthesis was provided, presenting all reported effects individually to capture the full range of findings and to avoid bias through selective aggregation. All evidence syntheses were visualized using forest plots. The outcomes Family functioning and sequential treatment models were synthesized narratively; however, exploratory forest plots were generated to illustrate the direction of effects across studies.

Heterogeneity (assessed only for pooled analyses) was evaluated using Cochran’s Q and quantified with the I² statistic [33]. In addition, the τ² estimate (between-study variance) was reported to quantify dispersion across studies, and Chi² tests were used to explore subgroup differences (e.g., post vs. follow-up). Analyses were conducted in R using the meta package (metagen) [44, 46].

To explore potential moderators of effect sizes and to allow a more differentiated examination of outcome domains, predefined subgroup analyses were planned. These included outcome domains: global, social, school/work, and substance-related functioning for psychosocial functioning and for psychopathology: internalizing, externalizing and general, rating perspective (clinician-, parent-, self-, and teacher-rated) and follow- up duration (> 12 months vs. ≤ 12 months) based on the full dataset. For readmission rates, subgroups were defined according to follow-up duration (> 12 months vs. ≤ 12 months). Where subgroup estimates were based on very small numbers of studies (k < 3), results were summarized descriptively or reported in the Supplementary Material (see Online Resource 11). No meta-regression was performed due to the limited number of studies (k < 10) [44].

Sensitivity analyses were performed to assess the robustness of the findings and to explore potential sources of heterogeneity. These analyses included the application of the DerSimonian–Laird estimator, inclusion of sequential HT models, exclusion of high-risk psychiatric emergency cohorts within MST studies, restriction to randomized controlled trials, exclusion of studies at high risk of bias, and harmonization across raters and outcome domains (see Online Resource 10).

### Certainty assessment

Publication bias was assessed as part of the GRADE evaluation for each outcome. In the case of fewer than ten studies per outcome, no formal assessment of publication bias (e.g., funnel plots or Egger’s test) was planned [47]. GRADE assessments were conducted separately for each outcome using Guideline Development Tool (GRADEpro GDT) [48], considering risk of bias, inconsistency, indirectness, imprecision, and publication bias. Certainty was rated as high, moderate, low, or very low [49] (see Online Resource 6).

## Results

### Study selection

**Fig. 1:**
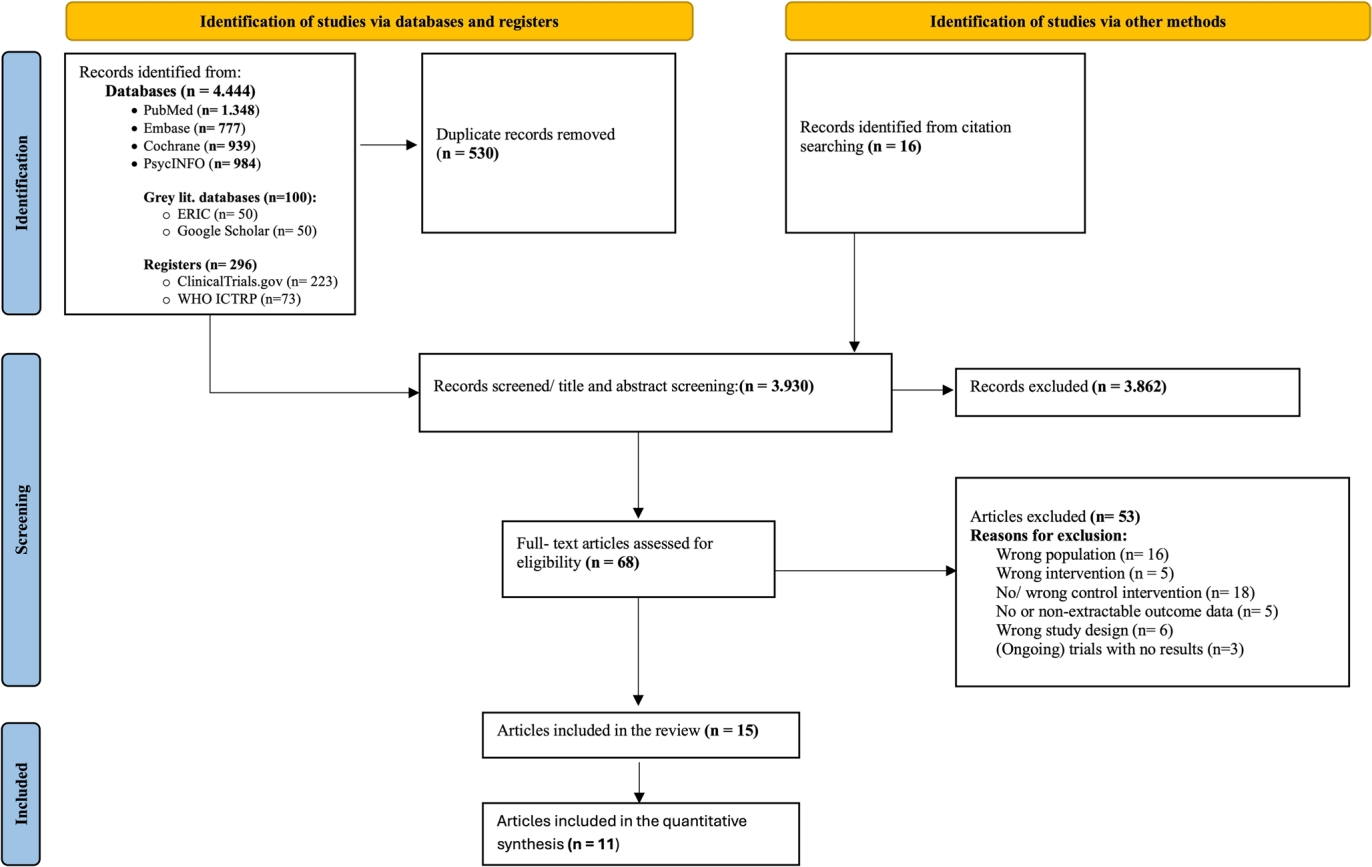
Prisma flow chart [29] illustrating the selection process

The initial database search yielded 4,460 records. After removing 530 duplicates, 3,930 records remained. After duplicate removal and title/abstract screening, a total of 15 studies were included based on a-priori eligibility and selection criteria (see Fig. 1). Of these, 11 studies provided sufficient data to be included in the quantitative synthesis. Four studies were not included in the meta-analysis because they either reported overlapping samples with more complete publications or did not provide sufficient statistical information to calculate effect sizes. In such cases, the most comprehensive dataset was retained. However, these studies were retained in the review to provide contextual information on intervention characteristics and study design (Table 1 and Online Resource 7).

**Table 1:** Characteristics of included studies: Home treatment and inpatient treatment

Author, Year, Country	Study type	No. (*n*)	Age (years)	Clinical population type	Intervention vs. Comparator	Assessment Timepoints	Relevant outcome measures	Included in meta-analysis	Risk of bias
Stand-alone interventions									
Henggeler et al., 1999 (USA) [23]	RCT	113 (IG: 57; CG: 56)	10–17 yrs. (M = 13)	Children and adolescents in psychiatric crisis (e.g., suicidality, psychosis, homicidal ideation, or other risk to self/others)	MST (4 months; daily visits → 3×/w; 24/7 crisis support) vs. Inpatient treatment (1–2 weeks); followed by a return to standard outpatient care	Discharge: MST (~ 4 months post recruitment)/Inpatient treatment: (1–2 weeks post recruitment)	Psychopathology: GSI-BSI, CBCL externalizing/internalizing (parent and teacher rated) Psychosocial functioning: FFS Self-esteem, CBCL Social, School attendance, arrest Family functioning: FACES-III -family cohesion and adaptability, GSI-BSI (caregiver)	Yes	High (RoB2)
Schoenwald et al., 2000, (USA) [56]	RCT	113 (IG: 57; CG: 56)	10–17 yrs. (M = 13)	Same sample as Henggeler et al. (1999)	Same intervention and comparator as Henggeler et al. (1999)	Discharge: MST (~ 4 months post recruitment)/Inpatient treatment: (1–2 weeks post recruitment)	Proportion of participants hospitalized. Mean days hospitalized. Mean days per hospitalized participants. Mean length of stay per episode for hospitalized participants	Yes	Some concerns (RoB2)
Huey et al., 2004, (USA) [58]	RCT	113 (IG: 57; CG: 56)	10–17 yrs. (M = 13)	Same sample as Henggeler et al. (1999)	Same intervention and comparator as Henggeler et al. (1999)	12 months post-treatment	Family functioning: FFS (parental control) Psychopathology: Depressive affect: BSI and CBCL, Hopelessness Scale for Children/Attempted suicide: CBCL and YRBS/Suicidal ideation: BSI and YRBS	Yes	High (RoB2)
Henggeler et al., 2003 (USA) [24]	RCT	113 (IG: 57; CG: 56)	10–17 yrs. (M = 13)	Same sample as Henggeler et al. (1999)	Same intervention and comparator as Henggeler et al. (1999)	Discharge: MST (~ 4 months post recruitment)/Inpatient treatment: (1–2 weeks post recruitment) 6 months post-treatment 12 months post-treatment	Psychopathology: GSI- BSI; CBCL Psychosocial functioning: FFS (self- esteem); School attendance Family functioning: FACES-III	No	High (RoB2)
Sheidow et al., 2004 (USA) [57]	RCT	113 (IG: 57; CG: 56)	10–17 yrs. (M = 13)	Same sample as Henggeler et al. (1999)	Same intervention and comparator as Henggeler et al. (1999)	Discharge: MST (~ 4 months post recruitment)/Inpatient treatment: (1–2 weeks post recruitment) 12 months post-treatment	Cost-effectiveness ratio: cost: Medicaid spending ($)/effectiveness: CBCL (externalizing and internalizing score); GSI	No	High (RoB2)
Graf et al., 2023 (Switzerland) [61] pilot evaluation	CBA (preference-based group allocation)	133 (IG: 37; CG: 96)	6–17 yrs. (M = 13.71)	Adolescents with acute mental disorders (affective, anxiety, behavioral); severe risk cases (e.g., suicidality, child welfare) excluded	AT_Home (avg. 84 days; 1×/d (60–120 min); 24/7 crisis line) vs. Inpatient treatment (no time limit; avg. 101 days)	Discharge	Psychopathology: HoNOSCA; HoNOSCA-SR	No	Moderate (ROBINS-I)
Graf et al., 2025 (Switzerland) [62]	CBA (preference-based group allocation)	75 (IG: 27; CG: 48)	6–17 yrs. (M = 15.92 yrs., SD = 2.87)	Same sample as Graf et al. (2023)	Same intervention and comparator as Graf et al. (2023)	18–24 months post-treatment	Psychopathology: HoNOSCA; HoNOSCA-SR Psychosocial functioning: GAF Readmission rates	Yes	Moderate (ROBINS-I)
Mattejat et al., 2001 (Germany) [51]	RCT	Mannheim: 27 (IG: 12; CG: 15/Marburg: 41(IG: 23; CG: 18)	M = 11.75 yrs. (SD = n/a)	Severe psychiatric disorders unresponsive to outpatient care; broad diagnoses incl. conduct, emotional, eating, ADHD, neurotic disorders	Home treatment (avg. 121 days; daily to weekly visits) vs. Inpatient treatment (avg. 91 days)	Discharge: M = 3.7 yrs. post-treatment (range: 2.1–5.2 yrs.)	Psychopathology: Number of marked symptoms (MSS) Psychosocial functioning: Rating of psychosocial competency: adaption at school or work	Yes	Some concerns (RoB2)
Schmidt et al., 2006 (Switzerland) [8]	CBA (preference-based group allocation)	105 (IG: 70; CG:35)	6–17 yrs. (IG: M = 10.9 yrs., SD = 3.0; CG: M = 11.3 yrs., SD = 3.1)	Children/adolescents with SGKJ ≤ 5 (inpatient-level need); excludes acute suicidality, psychosis, autism, rare disorders (< 4% prevalence)	Home treatment (max. 3 months; 2×/w → 1×/w + daily phone support (in crisis)) vs. Inpatient treatment (no time limit)	Discharge 1-year post-treatment	Psychopathology: MEI/blind ratings of symptom improvement (− 2 to + 4) Psychosocial functioning/Family Functioning: SGKJ; 5D-Scale (Marcus et al., 1993): family, school performance, peers, interests, autonomy)	Yes	Serious (ROBINS-I)
**Sequential HT interventions**									
Ougrin et al., 2018 (UK) [55]	RCT	106 (IG: 53; CG: 53)	12–17 yrs. (IG: M = 16.23 yrs., SD = 1.54; CG: M = 16.34 yrs., SD = 1.7)	Adolescents presenting with psychiatric emergencies; no exclusion based solely on risk level (if risk to self or others was deemed manageable).	SDS/ICCS (Early inpatient discharge (median 34 d), followed by flexible SDS/ICCS home/day care (mean total 116.3 d); daily contact possible; available 8–20 h + 24/7 on-call) vs. Inpatient treatment (median 50 days) followed by CAMHS outpatient treatment)	6 months after randomization	Psychopathology: SDQ; Self-harm questionnaire (multiple ≥ 5 episodes of self-harm) Psychosocial functioning: CGAS; School attendance/reintegration; Number of days not in education, employment, or training Cost-effectiveness ratio Mean total inpatient days	Yes	Some concerns (RoB2)
Ougrin et al., 2021 (UK) [59]	RCT	106 (IG: 53; CG: 53)	12–17 yrs. (IG: M = 16.23 yrs., SD = 1.54; CG: M = 16.34 yrs., SD = 1.7)	Same sample as Ougrin et al. (2018)	Same intervention and comparator as Ougrin et al. (2018)	6 months after randomization	Psychopathology: SHQ; CGI; HoNOSCA Psychosocial functioning: CIS Presentations to emergency departments with self-harm Total presentations to emergency departments Readmissions to inpatient psychiatric units Occupied bed-days whilst readmitted	Yes	Some concerns (RoB2)
Boege et al., 2014 (Germany) [53]	RCT	92 (IG: 51; CG: 41)	5–17 yrs.	Psychiatric disorders requiring inpatient care; broad spectrum incl. affective, anxiety, behavioral, eating & psychotic disorders	BeZuHG/HoT-BITS (Early inpatient discharge (avg. 48 days) + 12 weeks Hot-BITs hometreatment; 3×/week sessions; 5×/week crisis support (10 h/day); 24/7 on-call; monthly outpatient follow-up) vs. Inpatient treatment (avg. 70 d); outpatient follow-up (1×/month)	Discharge	Psychopathology: HoNOSCA, SDQ Psychosocial functioning: CGAS, CIS Length of inpatient stay	Yes	Some concerns (RoB2)
Boege et al., 2015 (Germany) [60]	RCT	92 (IG: 51; CG: 41)	5–17 yrs.	Same sample as Boege et al. (2014)	Same intervention and comparator as Boege et al. (2014)	Discharge 8.4 months post-treatment	Psychopathology: HoNOSCA; SDQ Psychosocial functioning: CGAS, CIS	Yes	Some concerns (RoB2)
Boege et al., 2015a (Germany) [54]	RCT	92 (IG: 51; CG: 41)	5–17 yrs.	Same sample as Boege et al. (2014)	Same intervention and comparator as Boege et al. (2014)	Discharge 8 months post-treatment	Psychosocial functioning: CGAS Cost-effectiveness ratio	No	Some concerns (RoB2)
Boege et al., 2021 (Germany)[52]	RCT	92 (IG: 51; CG: 41)	5–17 yrs.	Same sample as Boege et al. (2014)	Same intervention and comparator as Boege et al. (2014)	Discharge 8.4 months post-treatment 4.3 yrs. post-treatment	Psychopathology: HoNOSCA A/B Psychosocial functioning: CGAS	Yes	Some concerns (RoB2)

### Study and baseline characteristics

Fifteen studies published between 1999 and 2025 were included, covering four countries: Germany (*n* = 5), Switzerland (*n* = 3), the United Kingdom (*n* = 2), and the United States (*n* = 5). Twelve studies were randomized controlled trials (RCTs [22, 23, 50–59]. The remaining studies were classified according to the framework by Reeves et al. [30] as controlled before-and-after (CBA) studies with preference-based group allocation [8, 60, 61].

The total sample comprised 617 participants, with individual study sizes ranging from 68 to 133 (median = 105,5). Follow-up periods ranged from 6 months to 4.3 years. Participants were children and adolescents aged 6–17 years, primarily in early to mid-adolescence. Diagnoses covered a broad spectrum of internalizing and externalizing disorders, often comorbid. Consequently, ten studies examined children and adolescents experiencing psychiatric crises with marked functional impairment who might otherwise be considered for inpatient admission, but without an immediate life-threatening risk. In contrast to these ten studies, the MST trials (*n* = 5) included participants with more acute psychiatric emergencies, such as suicidality, homicidal ideation, psychosis, or other threats of harm to self or others warranting emergency hospitalization [22, 23, 55–57].

The included HT interventions were categorized into: (1) standalone HT programs (*n* = 9 studies), and (2) sequential models (*n* = 6) combining brief inpatient treatment with subsequent HT. An overview of the study characteristics, intervention, and comparator characteristics is provided in Table 1. Further details are presented in the Supplementary Material (Online Resource 7), including Table S1 (Overview of Study and Intervention Characteristics) and Table S2 (Summary of Outcomes, Risk of Bias, and Main Findings Across Included Studies). The pooled results of the meta-analyses are summarized in Table 2, while detailed study-level results are presented in the forest plots.

**Table 2:** Summary of pooled meta-analysis results

Outcome	Treatment model	Timepoint	k (studies)	Effect measure	Effect size	95%CI	I^2^ (%)
Psychosocial functioning	Standalone HT	Post treatment	4	Hedges‘g	−0.27	−0.80 to 0.26	35.8
Psychosocial functioning	Standalone HT	Follow-up	4	Hedges‘g	0.36	−0.13 to 0.85	16.5
Psychopathology	Standalone HT	Post treatment	5	Hedges‘g	−0.23	−0.77 to 0.30	66.7
Psychopathology	Standalone HT	Follow- up	5	Hedges‘g	0.05	−0.18 to 0.27	0
Readmissions	Standalone HT	Follow- up	3	Risk Ratio (RR)	1.41	0.98 to 2.01	0
Readmissions	All models combined	Follow-up	5	Risk Ratio (RR)	1.27	0.93 to 1.74	0

Random-effects meta-analyses were conducted using the Hartung–Knapp adjustment with the Paule–Mandel estimator. Negative values of Hedges’ g indicate better outcomes for inpatient treatment, whereas positive values indicate better outcomes for home treatment. For readmissions, risk ratios (RR) greater than 1 indicate higher readmission rates in the home treatment group

To ensure comparability across the 15 included studies, outcomes were categorized into four overarching domains. Table 3 provides a systematic overview of this mapping, assigning each individual study and instrument (e.g., CGAS, SDQ) to a specific domain. A key distinction should be noted regarding the number of studies: for instance, while 11 studies examined psychosocial functioning, only 6 were included in the quantitative meta-analysis (pooling). This reflects a rigorous methodological approach to avoid artificial inflation of results (*double counting*); specifically, follow-up reports of identical patient cohorts were excluded from the statistical synthesis. Detailed justifications for studies that were part of the systematic review but excluded from the meta-analysis are provided in the footnotes of Table 3.

**Table 3:** Overview of outcome domains, variables, and measurement instruments across the included studies

Outcome Domain	Variables/Symptom Domains	Instruments/Measures	No. of Studies (Total *N* = 15)	No. of Studies included in the Synthesis (Total *N* = 11)
**Psychosocial Functioning**	Global/overall, social, and school/educational functioning	CBCL (social subscale)[23], CGAS [52–55, 60], SGKJ [8], school attendance/adaptation[8, 23, 24, 51, 55], GAF [62], FFS (self-esteem subscale)[23, 24], 5D-Scale [8], CIS [53, 59, 60]	**11**	**6**^**a**^
**Psychopathology**	Internalizing, externalizing, general	CBCL (internalizing/externalizing subscales)[23, 24, 58], HoNOSCA[52, 53, 59–62], SDQ[53, 55, 60], MEI[8], MSS[51], GSI-BSI[23, 24, 58], Hopelessness Scale for Children[58], YRBS[58], SHQ[59], CGI[59]	**11**	**8**^**b**^
**Family Functioning**	Cohesion, adaptability, parental control, general	FACES-III[23, 24], FFS (Control)[23, 58], Five-Dimensional Assessment of Family Functioning[8]	**4**	**3**^**c**^
**Readmissions**	Any psychiatric rehospitalization	Readmission rates [8, 52, 56, 59, 62]	**5**	**5**

*5D-Scale * Five-Dimensional Assessment of Social Functioning; *CBCL* Child Behavior Checklist; *CGAS* Children’s Global Assessment Scale; *CIS* Children’s Global Assessment Scale – Impairment Scale; *CGI* Clinical Global Impression; *FACES-III* Family Adaptability and Cohesion Evaluation Scales; *FFS* Family Functioning Scale; *GAF* Global Assessment of Functioning; *GSI-BSI* Global Severity Index – Brief Symptom Inventory; *HoNOSCA* Health of the Nation Outcome Scales for Children and Adolescents; *MEI* Mannheim Parent Interview; *MSS* Marburg Symptom Scale; *SDQ* Strengths and Difficulties Questionnaire; *SGKJ* Skala zur Gesamtbeurteilung von Kindern und Jugendlichen/Global Assessment Scale for Children and Adolescents; *SHQ* Self-Harm Questionnaire; *YRBS *Youth Risk Behavior Survey

^a^ Exclusions: 4 follow-up reports (Boege et al. 2015, 2015a, 2021; Huey et al. 2004) and 1 study with missing data (Ougrin et al. 2021). Note: Pooling was only conducted for standalone models (*n* = 4); sequential models (*n* = 2) were displayed separately

^b^ Exclusions: 2 follow-up reports (Graf et al. 2023; Henggeler et al. 2003) and 1 study with missing data (Ougrin et al. 2021). Note: Pooling was conducted for standalone models (*n* = 5); sequential models (*n* = 3) were not pooled

^c^ Exclusion: 1 follow-up report (Henggeler et al. 2003) was excluded to avoid double-counting. Note: Although 3 reports provided data, no pooled effect size was calculated due to the limited number of independent study populations (two reports, Huey et al. 2004 and Henggeler et al. 1999, were based on the same cohort)

## Risk of bias in studies

### Risk of bias assessment (RoB2)

**Fig. 2:**
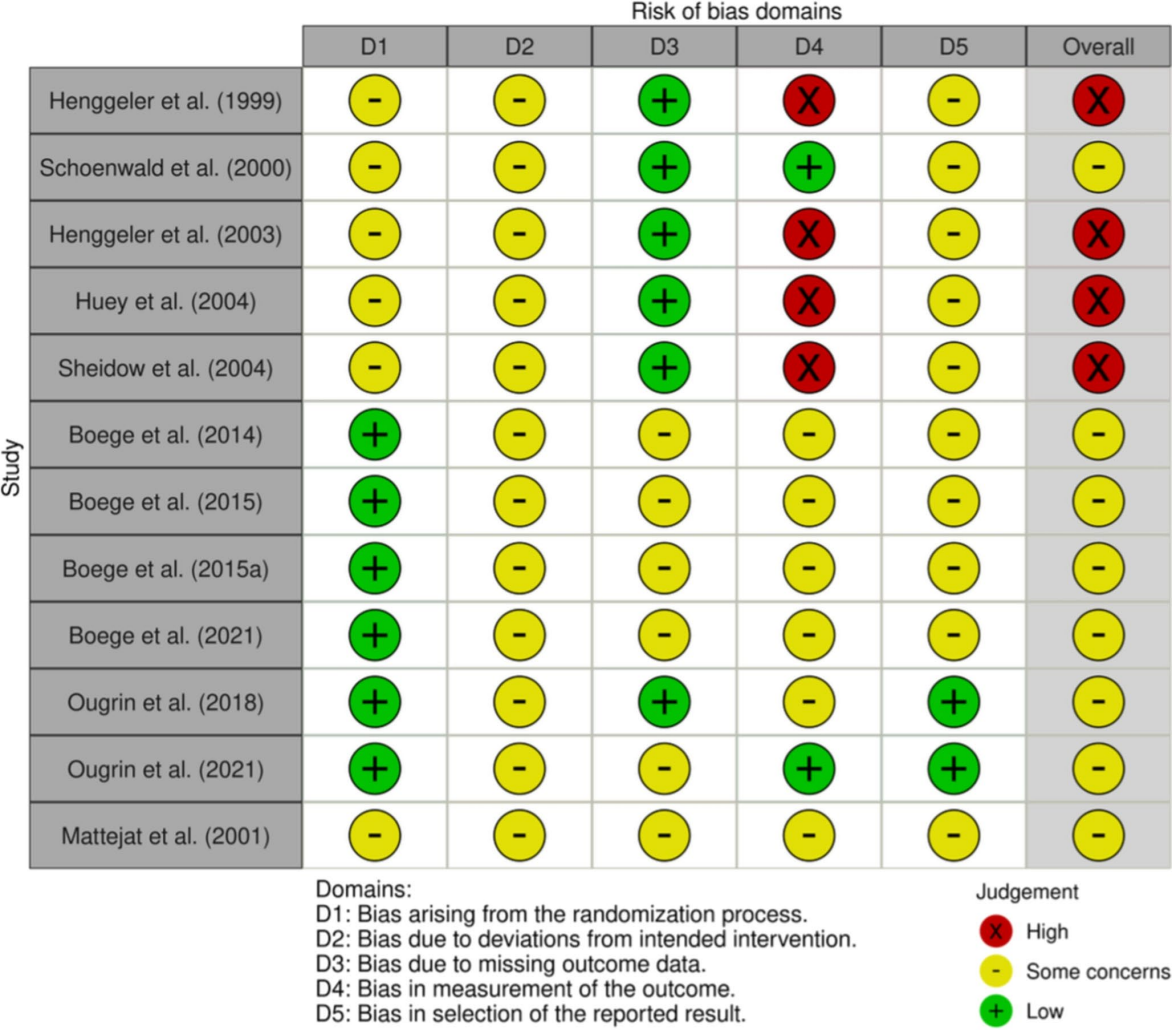
Risk of bias assessment using the RoB 2 tool

**Fig. 3:**
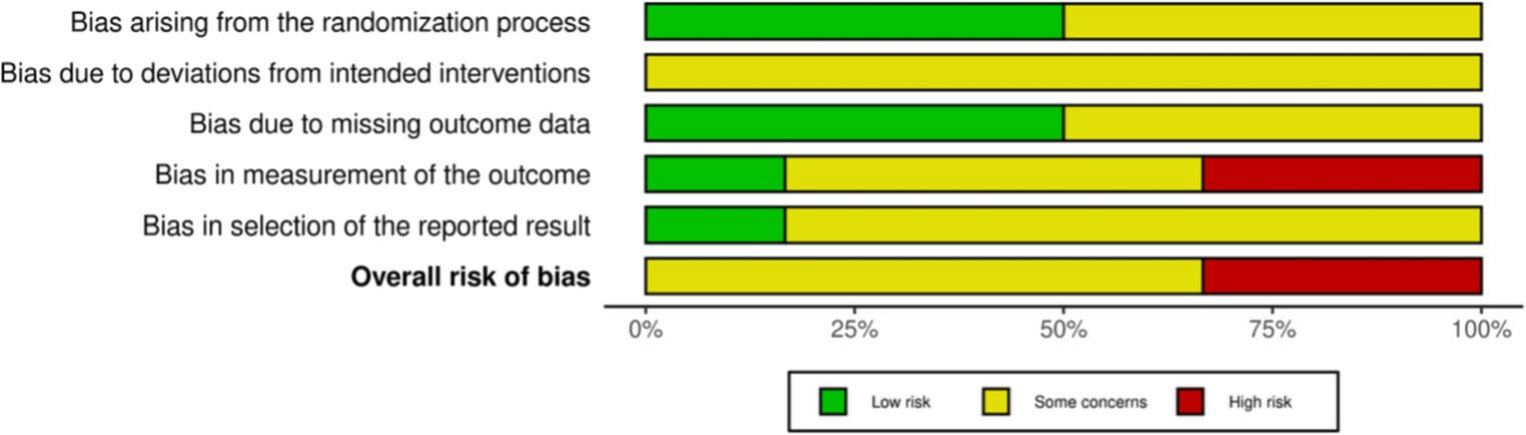
Summary plot of the RoB 2 quality appraisal showing the percentage of studies rated at low risk, some concerns, or high risk for each bias domain

The risk of bias in all included randomized controlled trials was assessed using the RoB 2. The primary trial by Henggeler et al., 1999 and the follow-awaup studies by Henggeler et al., 2003, Huey et al., 2004, and Sheidow et al., 2004 were rated as having a high risk of bias (Domain 4), as outcome assessors (parents and teachers) were not blinded. Consequently, their assessments were likely influenced by knowledge of the assigned intervention (e.g., due to satisfaction or dissatisfaction with allocation). In the follow-up study by Schoenwald et al. (2000), objective outcomes (e.g., length of stay) were assessed, which are not susceptible to this type of bias. All remaining studies were rated as having some concerns (see Figs. 2 and 3). Domain-specific assessments are provided in Online Resource 8.

#### Home treatment interventions

Across studies, treatment was primarily delivered in the patient’s home, although sessions could also take place in other relevant community settings such as schools or environments within daily life. Interventions were provided by multidisciplinary teams, including child and adolescent psychiatrists, psychologists, and nursing staff. In the study by Schmidt et al. treatment was carried out by psychiatric nurses and medical students under the supervision of child and adolescent psychiatrists [8]. The duration of home treatment ranged from approximately three to four months, with contact frequency varying from weekly sessions to daily visits. Teams were accessible 24/7 through telephone support. In most studies, hospitalization remained possible in cases of acute self- or other endangerment requiring continuous supervision. Only Mattejat et al. did not explicitly describe procedures for hospitalization during the home treatment phase [50]. Only MST represents a fully manualized intervention [22, 23, 55–57], whereas the remaining programs were service-based home treatment models without a standardized treatment manual. Across studies, treatment included multimodal approaches such as psychotherapeutic interventions (e.g., cognitive-behavioral therapy), family-focused interventions (e.g., family therapy or parent training), and case management addressing the psychosocial environment.

#### Comparison interventions

Across studies, the comparison intervention consisted of standard hospital-based inpatient psychiatric treatment delivered in specialized child and adolescent psychiatric units. Treatment was provided by multidisciplinary teams including psychiatrists, psychologists, nursing staff, and allied professionals such as social workers or educators. Inpatient treatment comprised multimodal therapeutic programs including psychiatric assessment, individual and group psychotherapy, family interventions, and pharmacological treatment. The duration of inpatient treatment varied across studies, ranging from short-term crisis hospitalization (approximately 1–2 weeks in Henggeler et al., 1999) to longer admissions of approximately three months (mean ≈ 101 days in Schmidt et al., 2006), depending on clinical need and the respective treatment model. Overall, the MST studies (*n* = 5) used brief crisis-oriented inpatient admissions, whereas the remaining studies (*n* = 10) described more comprehensive therapeutic programs without predefined time limits.

### Risk of bias in non-randomized studies (ROBINS-I)

**Fig. 4:**
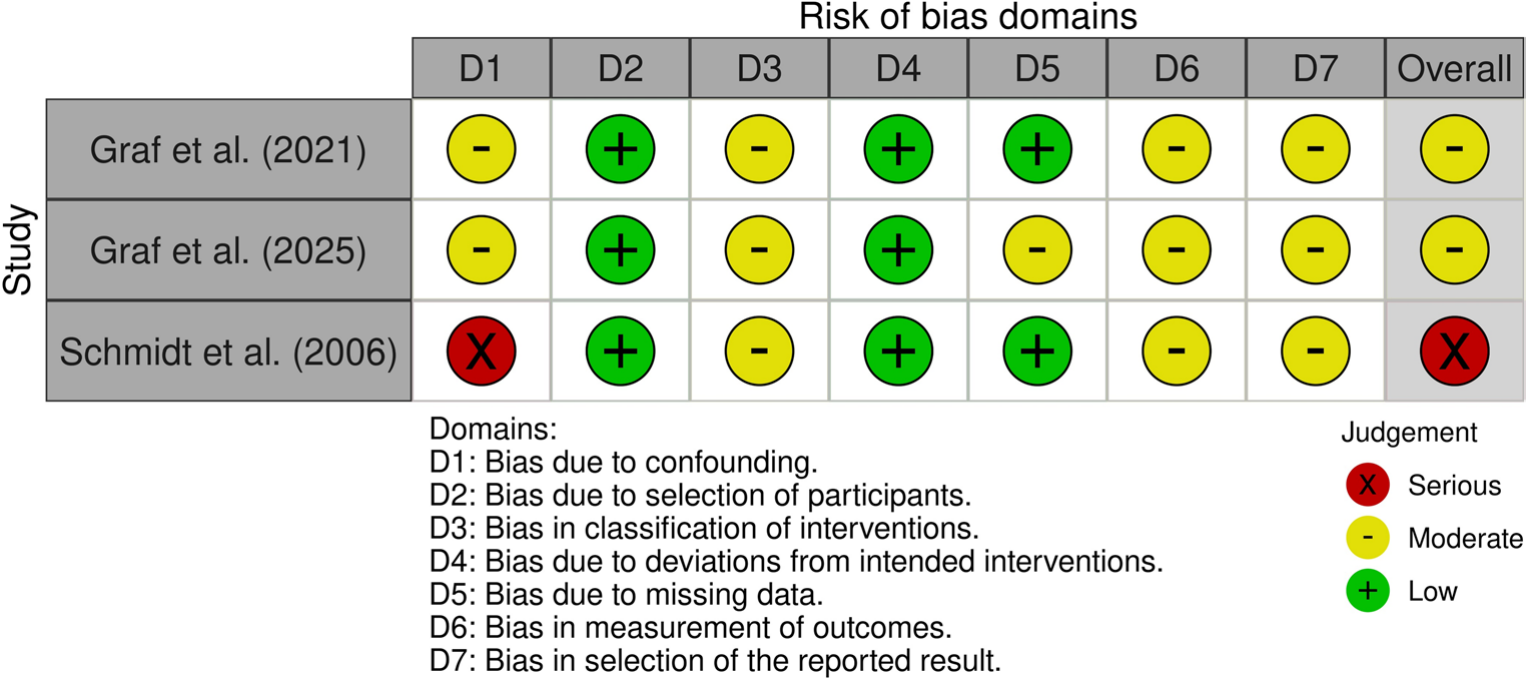
Risk of bias assessment using the ROBINS-I tool. The traffic light plot displays judgments for each bias domain across all included non-randomized studies (green = low risk, yellow = moderate risk, red= serious risk)

Risk of bias in the three included non-randomized studies was assessed using the ROBINS-I tool. The most prominent source of bias was confounding due to non-randomized allocation (Domain 1). One study (Schmidt et al., 2006) was rated as having a serious risk of bias, as group allocation was based on participant preferences, without statistical adjustment for confounders. In contrast, the studies by Graf et al. were judged to have a moderate risk of bias, as they applied partial randomization or statistical adjustment methods (e.g., Augmented Inverse Probability Weighting). Across the remaining domains, the risk of bias was generally rated as low to moderate (Fig 4).

## Results of synthesis

**Fig. 5:**
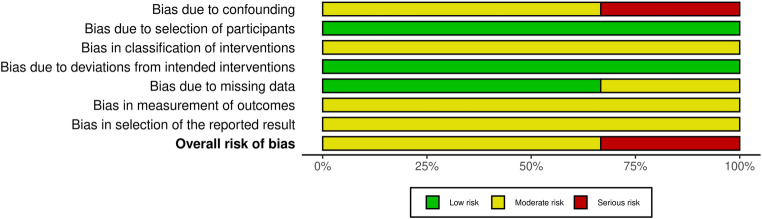
Summary plot of the ROBINS-I quality appraisal showing the percentage of studies rated at low, moderate, serious, or critical risk of bias for each domain

Of the fifteen included studies, eleven provided sufficient quantitative data and were therefore included in the meta-analyses, as indicated in Table 1 (column “Included in meta-analysis”) (Fig 5).

### Psychosocial functioning: stand- alone and sequential models

**Fig. 6:**
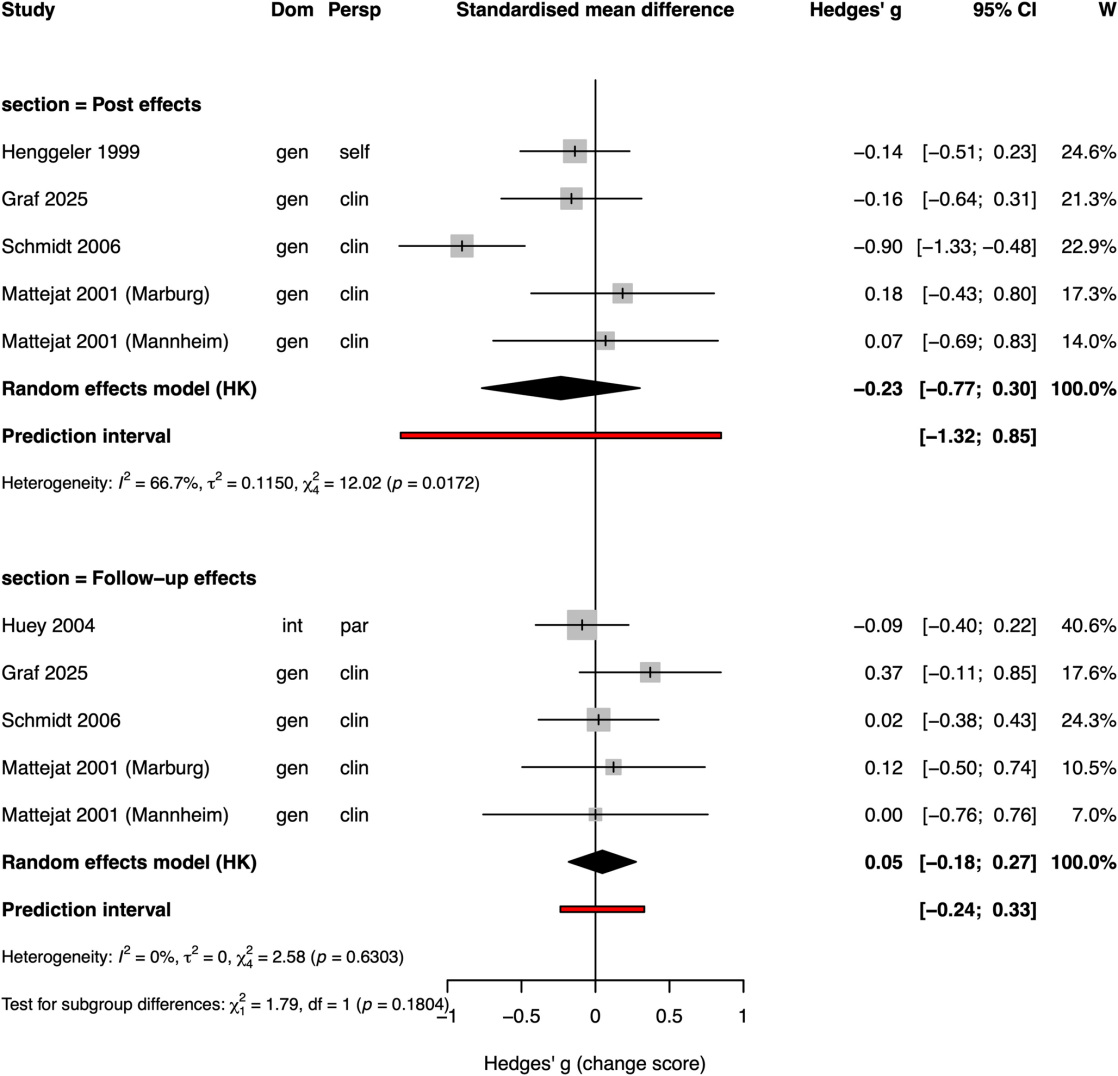
Forest plot of pre–post and follow-up effect sizes (Hedges’ g) for psychopathology in stand-alone studies. Random-effects estimates were calculated using the Hartung–Knapp adjustment with the Paule–Mandel estimator; τ² and 95% prediction intervals are reported. I² was not interpreted when k ≤ 2 (k = number of independent comparisons). Negative values indicate better outcomes for inpatient treatment; positive values indicate better outcomes for home treatment. Abbreviations: Dom = symptom domain (gen = general, int = internalizing, ext = externalizing); Persp = rating perspective (clin= clinician-rated, self = self-rated, par = parent-rated); CI = confidence interval; W = weight; HK = Hartung–Knapp

**Fig. 7:**
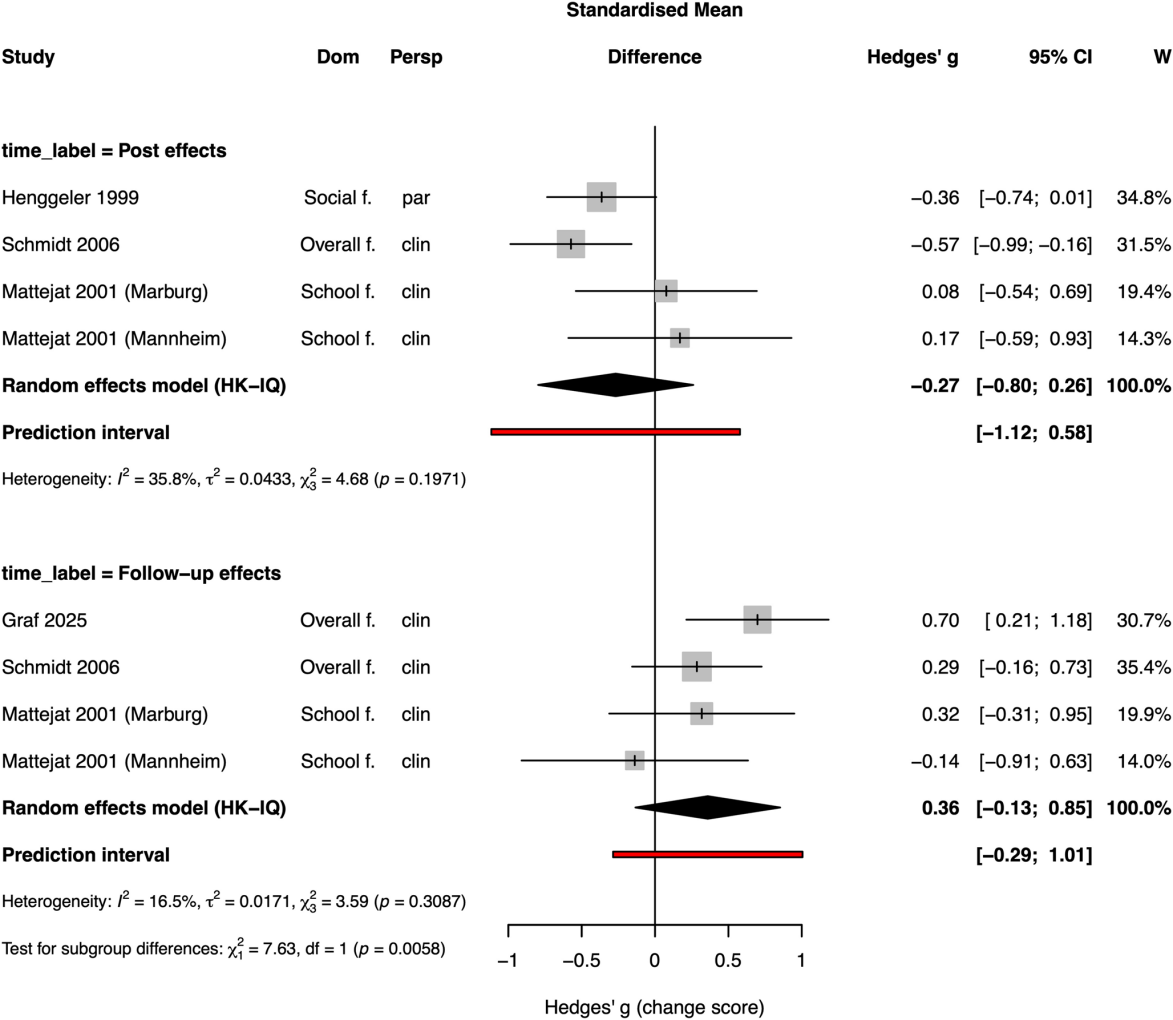
Forest plot of pre–post and follow-up effect sizes (Hedges’ g) for psychosocial functioning in stand-alone models. Random-effects estimates were computed using the Hartung–Knapp adjustment with the Paule–Mandel estimator; τ² and 95% prediction intervals are reported. I² was not interpreted when k ≤ 2 (k = number of independent comparisons). Negative values indicate better outcomes for inpatient treatment, positive values indicate better outcomes for home treatment. Abbreviations: Dom = domain; f. = functioning; Persp = rating perspective (clin = clinician-rated, self = self-rated, par = parent-rated); CI = confidence interval; W = study weight; HK = Hartung–Knapp adjustment

 A total of 11 studies reported outcomes related to psychosocial functioning for both stand-alone and sequential models. Of these, 6 studies provided sufficient data for quantitative synthesis (see Table 3 for details) For stand-alone models in the post-treatment period (k = 4; N_IG_: 162; N_CG_:124), there was no significant difference between home treatment and inpatient treatment (Hedges’ g = −0.27, 95% CI [−0.8, 0.26]; I² = 35.8%). At follow-up (k = 4; N_IG_: 121; N_CG_: 109), the pooled effect remained non-significant (Hedges’ g = 0.36, 95% CI [−0.13, 0.85]; I² = 16.5%) (see Fig. 6). This subgroup comparison between post-treatment and follow-up effects revealed a statistically significant change over time (χ² = 7.63, *p* = 0.0058). Heterogeneity was moderate at post-treatment (I² = 35.8%) and low at follow-up (I² = 16.5%) (see Fig. 7).

Sensitivity analyses produced effect estimates similar in magnitude and direction to the primary analysis. When sequential treatment models were included, the pooled follow-up effect reached statistical significance (g = 0.37, 95% CI [0.07, 0.67]). All other sensitivity analyses—including rater-harmonized analyses, analyses restricted to randomized controlled trials, analyses excluding studies with high risk of bias or psychiatric emergency cases during MST treatment, and analyses using the DerSimonian–Laird estimator—produced comparable effect sizes that remained non-significant and were consistent with the primary analysis. The subgroup difference between post-treatment and follow-up effects observed in the primary analysis was not consistently reproduced across all sensitivity analyses (see Online Resource 7 Figs. 1, 2, 3, 4, 5 and 6).

Exploratory subgroup analyses by outcome domain, follow-up duration, and rater perspective are presented in the Supplementary Material (see Online Resource 11). None of the pooled subgroup effects reached statistical significance. Tests for subgroup differences were not performed because only one subgroup could be pooled in each analysis.

**Fig. 8:**
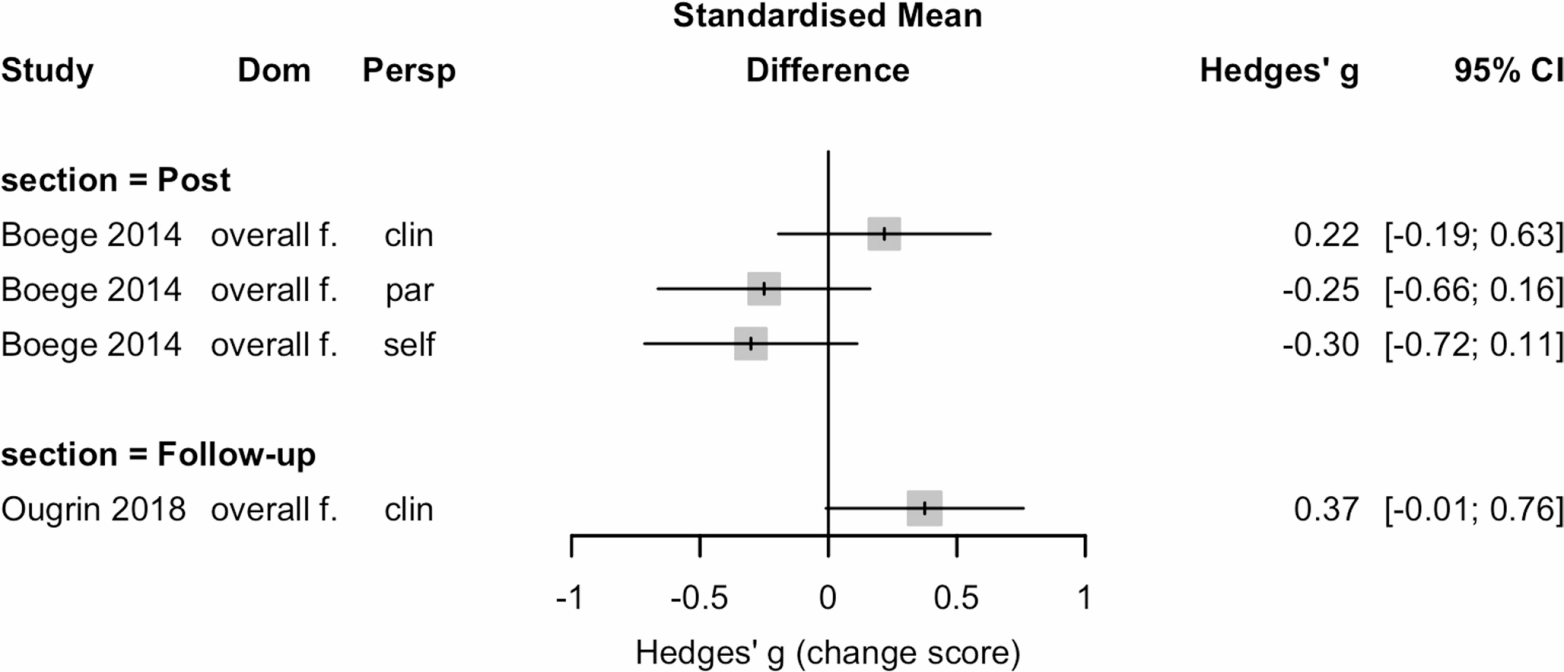
Exploratory Forest plot of pre–post and follow-up effect sizes (Hedges’ g) for psychosocial functioning in sequential models. No pooling was performed because k ≤ 2 (k = number of independent comparisons). Negative values indicate better outcomes for inpatient treatment; positive values indicate better outcomes for home treatment. Abbreviations: Dom = domain; f. = functioning; Persp = rating perspective (clin = clinician-rated, self = self-rated, par = parent-rated); CI = confidence interval

Post-treatment effects in the sequential studies were reported in one study (Boege et al., 2014) and varied across rater perspectives. Clinician ratings (CGAS) yielded an effect size of g = 0.22; 95% CI [− 0.19, 0.63], whereas parent- and self-ratings (CIS) showed effect sizes of g = −0.25; 95% CI [−0.66, 0.16] and g = −0.30; 95% CI [− 0.72, 0.11], respectively. At follow-up, one study (Ougrin et al., 2018) reported a clinician-rated effect size of g = 0.37; 95% CI [− 0.02, 0.77]) Due to the small number of studies, no meta-analysis, sensitivity analyses, or subgroup analyses were conducted; results are therefore reported descriptively (see Fig. 8).

### Psychopathology: stand alone and sequential models

A total of 11 studies reported outcomes related to psychopathology for both stand-alone and sequential models. Of these, 8 studies were included in the quantitative synthesis (see Table 3 for details) For stand-alone models at post-treatment (k = 5; N_IG_: 179; N_CG_: 169), there was no significant between-group difference (Hedges’ g = − 0.23, 95% CI [–0.77, 0.30]; I²= 66.7%). At follow-up (k = 5; N_IG_: 199; N_CG_: 187), the pooled effect remained non-significant (Hedges’ g = 0.05, 95% CI [–0.18, 0.27]; I² = 0%). The difference between post-treatment and follow-up effects was not significant (χ² = 0.90, *p* = 0.3439).

A series of sensitivity analyses was conducted to examine the robustness of the findings. Restricting the analysis to randomized controlled trials (RCTs) resulted in pooled effects of g = −0.04 (95% CI −0.46 to 0.38; k = 3) at post-treatment and g = −0.04 (95% CI −0.62 to 0.54; k = 3) at follow-up. Excluding studies with high risk of bias yielded pooled effects of g = −0.01 (95% CI −0.49 to 0.46; k = 3) at post-treatment and g = 0.22 (95% CI −0.25 to 0.69; k = 4) at follow-up. In both cases, heterogeneity was no longer observed (I² = 0%). Additional sensitivity analyses-including domain-harmonized analyses, inclusion of sequential designs, rater-harmonized analyses, analyses using the DerSimonian–Laird estimator, and analyses excluding psychiatric emergency cases during MST treatment- yielded comparable pooled estimates and did not materially change the results (see Online Resource 10, Figs. 7, 8, 9, 10, 11, 12 and 13).

Exploratory subgroup analyses by outcome domain, follow-up duration, and rater perspective are presented in the Supplementary Material (see Online Resource 11). None of the pooled subgroup effects reached statistical significance. Tests for subgroup differences were not performed because only one subgroup could be pooled in each analysis.

For sequential models (k = 2 at post-treatment and k = 2 at follow-up; note that Boege 2014 and 2015 refer to the same study population), results assessing general symptom domains were mixed across rater perspectives. At post-treatment, both parent and clinician ratings showed statistically significant effects towards inpatient treatment (parent-rated: g = − 0.65, 95% CI [− 1.07, − 0.22]; clinician-rated: g = − 0.42, 95% CI [− 0.83, − 0.00]). In contrast, self-ratings did not indicate a significant between-group difference (g = 0.20, 95% CI [− 0.21, 0.61]).

 At follow-up, no statistically significant between-group differences were observed for either clinician ratings (g = 0.10, 95% CI [− 0.32, 0.51]) or self-ratings (g = 0.32, 95% CI [− 0.07, 0.71]). Parent ratings were not available at this time point.

**Fig. 9:**
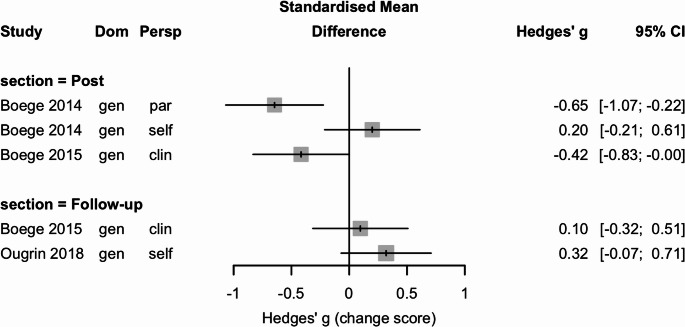
Exploratory Forest plot of pre–post and follow-up effect sizes (Hedges’ g) for psychopathology in sequential models. No pooling was performed because k ≤ 2 (k = number of independent comparisons). Negative values indicate better outcomes for inpatient treatment; positive values indicate better outcomes for home treatment. Abbreviations: Dom = symptom domain (gen = general, int = internalizing, ext = externalizing); Persp = rating perspective (clin = clinician-rated, self = self-rated, par = parent-rated); CI = confidence interval; W = weight; HK = Hartung–Knapp

Due to the small number of studies, no meta-analysis, sensitivity analyses, or subgroup analyses were conducted; results are therefore reported descriptively (see Fig. 9).

### Family functioning

 A total of 4 studies reported outcomes related to family functioning for both stand-alone and sequential models. Of these, 3 studies were included in the synthesis, while 1 follow-up report (Henggeler et al. 2003) was excluded (see Table 3 for details).

Given the limited evidence base (k = 3 reports; however, two were based on the same study population: Huey et al. 2004 and Henggeler et al. 1999), study-level effects are presented narratively and in forest plots without meta-analytical pooling.

**Fig. 10:**
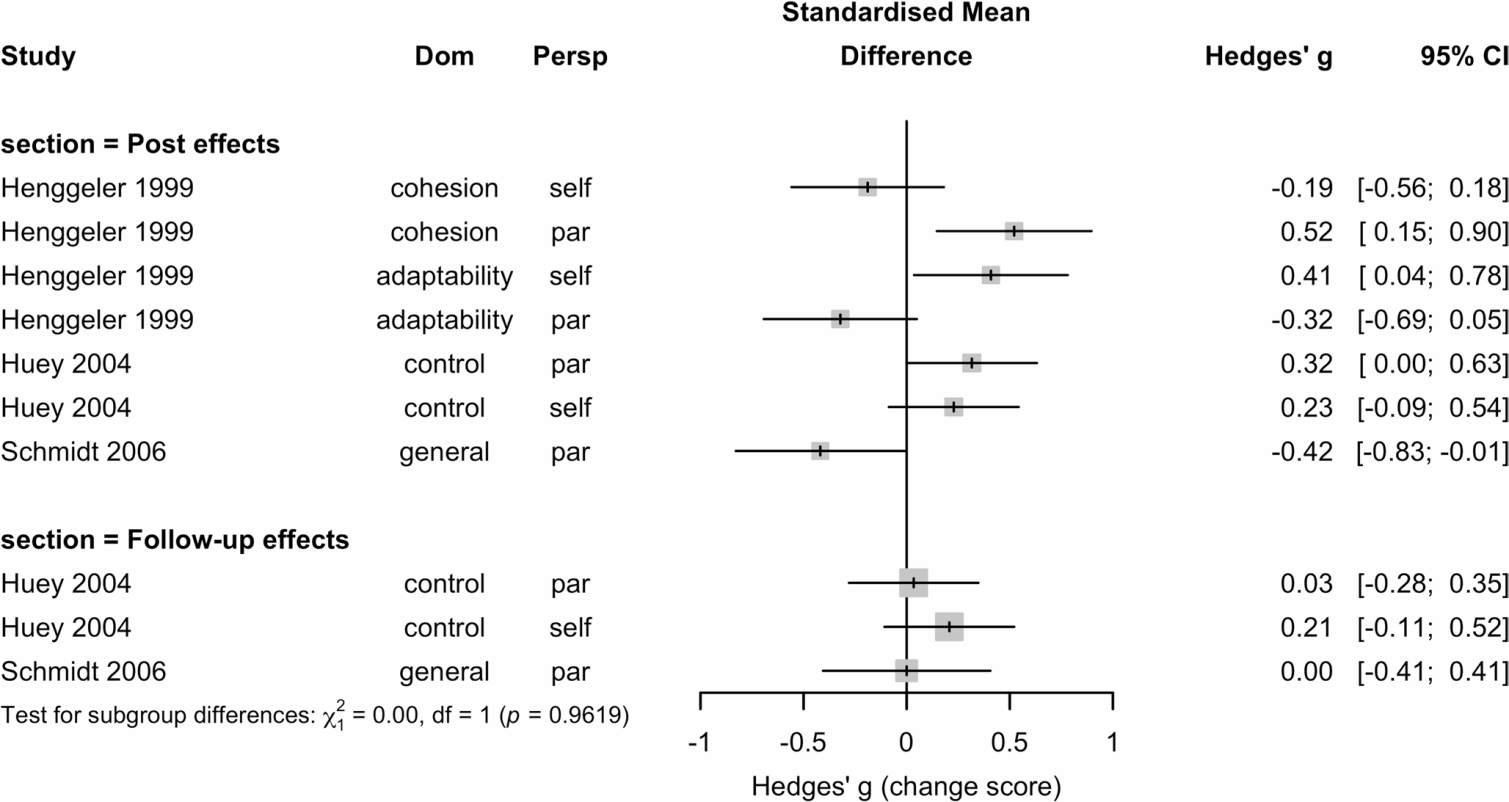
Exploratory Forest plot of pre–post and follow-up effect sizes (Hedges’ g) for family functioning. No pooled estimate was calculated because k ≤ 2 (k = number of independent comparisons). Henggeler (1999) and Huey (2003) refer to the same study population. Negative values indicate better outcomes for inpatient treatment, positive values for home treatment. Abbreviations: Persp = rating perspective (clin = clinician-rated, self = self-rated, par = parent-rated); CI = confidence interval

 At post-treatment, results varied across domains and rater perspectives (Fig. 10). Statistically significant effects were observed for parent-rated cohesion (g = 0.52, 95% CI [0.15, 0.90]), self-rated adaptability (g = 0.41, 95% CI [0.04, 0.78]), and parent-rated control (g = 0.32, 95% CI [0.00, 0.63]), indicating better outcomes for the intervention. One significant effect favoring the comparator was observed for parent-rated general family functioning (g = −0.42, 95% CI [−0.83, −0.01]).

At follow-up, none of the effects across domains and raters were statistically significant (Fig. 10).

###  Readmissions

A total of 5 studies reported psychiatric readmission rates for both stand-alone and sequential models. All 5 studies provided sufficient data and were included in the quantitative synthesis (see Table 3 for details).

**Fig. 11:**
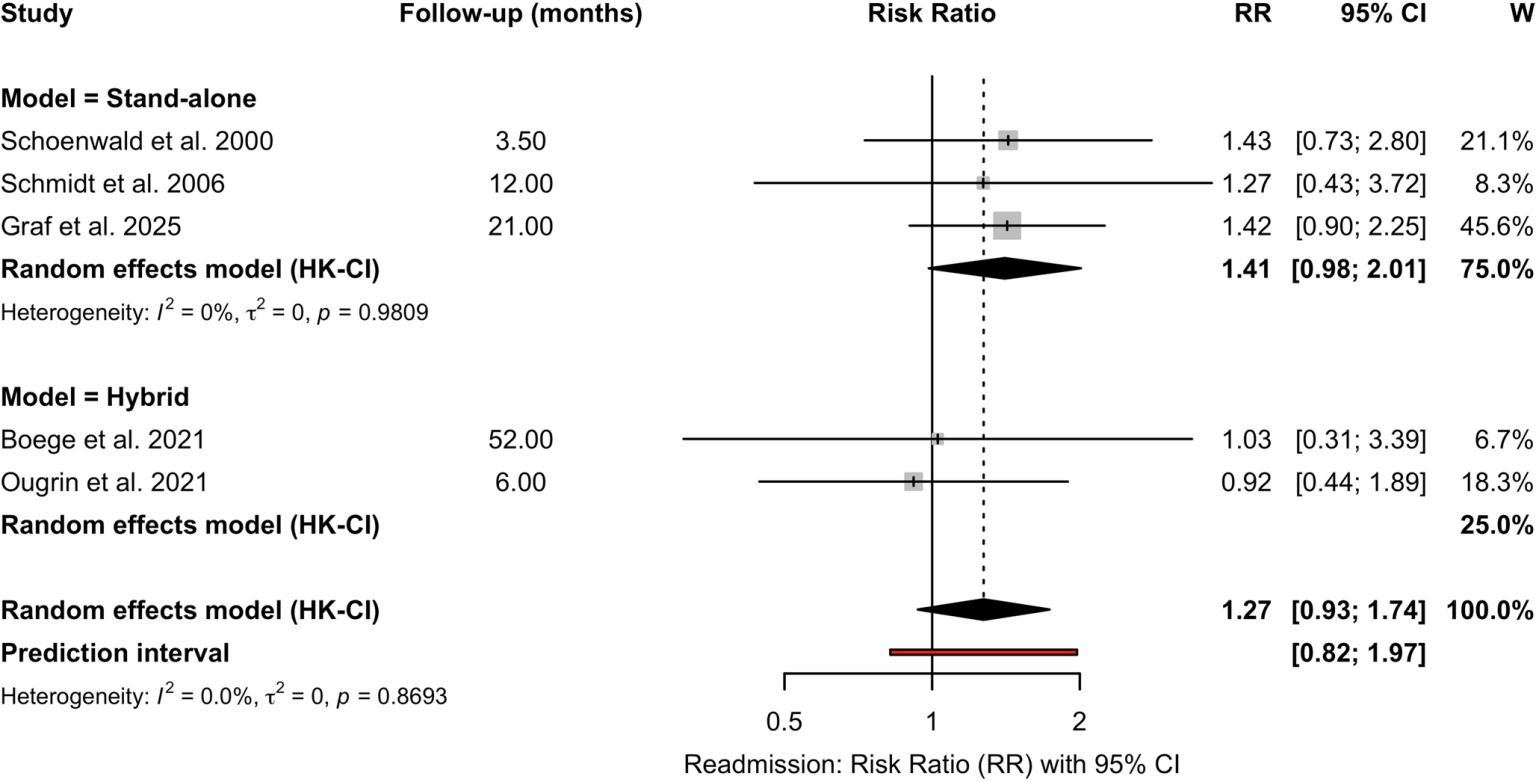
Forest plot of pooled log risk ratios (ln RR) for readmissions based on a random-effects model using the Hartung–Knapp adjustment with the Paule–Mandel estimator. Diamonds represent pooled effects, expressed as exp (pooled ln RR) with corresponding 95% confidence intervals. RR > 1 indicates higher readmission rates in the intervention group (home treatment) compared with inpatient treatment. Abbreviations: RR = risk ratio; HK–CI = Hartung–Knapp confidence interval; W = weight

Across these studies, the pooled random-effects estimate indicated no significant difference in readmission rates between HT and IT (RR = 1.27, 95% CI [0.93–1.74]). For stand-alone models (k = 3) the pooled estimate was RR = 1.41, 95% CI [0.98–2.01] with no observed heterogeneity (I² = 0%) (see Fig. 11). For sequential models (k = 2), no pooled estimate was calculated due to insufficient study numbers; individual study estimates were not statistically significant. Follow-up durations varied substantially (3.5 months, 12 months, 18–24 months [median 21 months], 52 months).Sensitivity analyses (without ad hoc variance correction; using the DerSimonian–Laird estimator; using a common-effects model; excluding studies with high risk of bias; restricting analyses to randomized controlled trials; excluding MST psychiatric emergency cases; and including sequential models) did not change the results (see Online Resource 10; Figs. 14, 15, 16, 17, 18, 19 and 20). An exploratory subgroup analysis based on follow-up duration (≤ 1 year vs. > 1 year) showed no statistically significant subgroup difference (χ² = 0.71, *p* = 0.40) (see Online Resource 11; Fig. 11).

### Reporting biases and certainty of evidence

 The quality of evidence was assessed using the GRADE approach across the domains of risk of bias, inconsistency, indirectness, and imprecision [49]. Overall, the certainty of evidence ranged from very low to moderate. For stand-alone home treatment models, evidence for psychopathology, family functioning and psychosocial functioning was mostly rated as very low to low, mainly due to risk of bias, inconsistency, and imprecision. For sequential models, evidence for psychopathology and psychosocial functioning reached low to moderate certainty, limited primarily by imprecision. No data on family functioning were available for sequential models, indicating a relevant evidence gap in this domain Considerable inconsistency was observed across studies, reflecting heterogeneity that could only partially be explained through sensitivity analyses, which were constrained by the limited number of available studies. Imprecision represented another major limitation, as the confidence intervals frequently encompassed both positive and negative effects. This pattern reflects inconsistent directions of effects, with inconsistent findings across studies For readmissions, the certainty of evidence was low for both model types. In sequential models, this was mainly due to serious imprecision and the limited number of available studies, whereas for stand-alone models, the evidence was downgraded because of risk of bias and imprecision

Publication bias was unlikely, as suggested by a comprehensive search including grey literature and trial registries; however, statistical tests such as Egger’s regression or funnel plots were not conducted due to < 10 studies per outcome [47]. Detailed GRADE assessments are provided in the Summary of Findings (see Online Resource 6).

## Discussion

This systematic review and meta-analysis compared the effectiveness of HT with IT for children and adolescents in psychiatric crises. Across the outcomes - psychopathology, psychosocial functioning, family functioning, and readmission no consistent superiority of either intervention was observed. Overall, both treatment settings achieved comparable improvements, suggesting that psychiatric crises can be managed effectively in both inpatient and home-treatment settings. In contrast to previous analyses, the present study distinguished between stand-alone home treatment models and sequential models that combine a brief inpatient stabilization phase with subsequent home-based continuation of care. Moreover, the comparison group was restricted to hospital-based inpatient psychiatric treatment with continuous clinical supervision. To further explore potential differences between treatment approaches, subgroup analyses were conducted to examine structural and temporal variations in treatment effects:

A relevant finding concerns the time course of effects. Although pooled estimates for **psychosocial functioning** were not statistically significant, subgroup analyses indicated a statistically significant difference between post-treatment and follow-up effects. Immediately after treatment, inpatient treatment tended to yield better results, whereas at follow-up, effects tended to favor home treatment. A possible explanation is that inpatient treatment may provide short-term relief by temporarily removing children and adolescents from stressful environments [61]. In contrast, home treatment integrates therapeutic interventions within the family context and daily routines, which may facilitate the transfer of therapeutic strategies into everyday life and has been suggested to support longer-term adaptation [20]. However, long-term follow-up data remain limited [51], and this pattern was not consistently reproduced across all sensitivity analyses (see Online Resource 7, Figs. 1, 2, 3, 4, 5 and 6).

The influence **of treatment duration** may represent an additional aspect affecting outcomes. Across studies, HT was often delivered over longer periods than inpatient treatment [22, 50, 52, 57], in some cases with substantial differences (e.g., in Henggeler et al. 1999: 122 vs. 5.77 days). These discrepancies likely reflect structural characteristics of the treatment models rather than differences in effectiveness, with inpatient treatment focusing on shorter, intensive stabilization and HT providing longer-term support within the family context. Consistent with this, Graf et al. (2024) found no overall difference in treatment duration, albeit with high heterogeneity [27].

For **psychopathology**, effect estimates across stand-alone models were close to zero at both post-treatment and follow-up, indicating no significant differences between HT and IT at either time point. This suggests that both treatment approaches may be considered equivalent regarding symptom reduction. Consistent with previous studies, both interventions were associated with clear and stable improvements in psychopathology over time compared to baseline [8, 22, 50, 62, 63]. However, noticeable heterogeneity was observed (I² = 66.7% at post-treatment). This variability appears to be multifactorial and was only partly explained by the conducted sensitivity analyses. While restricting analyses to randomized controlled trials and excluding studies with high risk of bias substantially reduced statistical heterogeneity, this effect may partly reflect the exclusion of individual studies with outlying effect estimates (e.g., Schmidt et al., 2006) as well as the reduced number of included studies. Taken together, these findings suggest that heterogeneity in this field is not solely methodological but also reflects differences in treatment models, their implementation, and broader contextual factors- such as variations in healthcare systems- that are not fully captured in the available quantitative data.

Evidence regarding **sequential treatment models** remains limited, with only two independent study populations identified (Boege et al.; Ougrin et al.). For psychosocial functioning, no consistent advantage of either treatment setting was observed. Parent- and self-ratings tended to favor inpatient treatment immediately after treatment, possibly reflecting continued stabilization within the structured clinical environment, whereas home treatment participants had already been re-exposed to family-related stressors [52]. However, no superiority of either intervention was evident in the long term, as also reflected by the absence of group differences at follow-up [51]. A similar pattern was observed for psychopathology. While parent- and clinician-rated outcomes significantly favored inpatient treatment at post-treatment, this advantage was no longer evident at follow-up. A plausible explanation is short-term symptom stabilization resulting from temporary removal from high-conflict family environments [8, 60]. In contrast, self-ratings tended to favor home treatment, although effects were small and not statistically significant. These discrepancies across rater perspectives further underline the heterogeneity of findings.

For **family functioning**, some studies reported advantages in favor of home treatment; however, the evidence base is limited, as findings are largely derived from two MST studies within the same population [22, 57]. Parents reported greater family cohesion under HT [22], possibly due to stronger involvement in therapy and improved understanding of the illness [64]. In contrast, adolescents initially reported a decline in cohesion, followed by later improvement, which has been interpreted as a reaction to increased structure and parental monitoring [23]. Adolescents also rated family adaptability (i.e., family structure) significantly more positively under HT, which may reflect the direct integration of new rules and routines into the family context, thereby facilitating their implementation in everyday life [8]. In contrast parents perceived greater structural improvements following inpatient treatment, possibly because the more structured clinical environment may be viewed as providing clearer organization than the home setting [22].

Across both model types, the pooled RR of 1.27 (95% CI: 0.93–1.74) indicates no evidence of reduced **readmission** risk in home treatment compared to inpatient treatment. Notably, within stand-alone models, the direction of effects even tended to favor inpatient treatment, although confidence intervals were wide and crossed unity. Thus, the commonly held assumption that home treatment reduces readmissions by facilitating a better transfer of treatment gains into everyday life cannot be clearly supported based on the current evidence, aligning with recent meta-analytic findings [61].The nonsignificant subgroup test further suggests that readmission outcomes do not differ meaningfully between stand-alone and sequential service models.

The present meta-analysis represents an initial attempt to structure outcomes into clinically meaningful domains. Primary outcomes (e.g., psychopathology, psychosocial functioning, and family functioning) were categorized into corresponding **subdomains** (see Online Resource 11), to allow a more differentiated analysis of potential treatment effects. This approach may help to identify whether specific patient groups (e.g., internalizing vs. externalizing disorders) benefit differently from home versus inpatient treatment.

For example, findings on school functioning as a subdomain of psychosocial functioning were inconsistent and based on limited data, suggesting that outcomes in this domain may depend on whether educational reintegration is explicitly addressed within the treatment model [22, 52, 54, 58]. However, the current evidence base remains limited overall, as most subgroup analyses were based on a small number of studies.

## Limitations

Despite the rigorous methodology of this systematic review and meta-analysis, several limitations should be considered when interpreting the findings:

First, the number of available (randomized) trials comparing home treatment with inpatient treatment in children and adolescents remains limited, and many studies included small sample sizes, reducing statistical power. Consequently, overall pooling was not feasible for sequential models, the outcome family functioning, and several subgroup analyses, as fewer than three studies were available. This also meant that certain subgroup comparisons (e.g., internalizing vs. externalizing domains or rater perspectives) could not be meaningfully tested when fewer than two pooled subgroups were present. Furthermore, because fewer than ten studies per outcome were available, meta-regression was not conducted in accordance with Cochrane guidelines [44]; instead, leave-one-out sensitivity analyses were applied, which also have limited interpretability when only few studies exist [65].

Second, substantial statistical heterogeneity was observed for the outcome psychopathology at post-treatment. Although heterogeneity decreased to zero when analyses were restricted to randomized controlled trials, the number of available RCTs was too small to draw stable conclusions.

Third, although standardized mean differences were used to ensure comparability across different measurement instruments, conceptual variation across scales and rater perspectives may nevertheless have contributed to variability in effect directions.

Fourth, incidence rate ratios (IRRs) could not be calculated because the number of readmissions per patient was not reported; only the occurrence of at least one readmission event (yes/no) was available. In addition, follow-up durations varied considerably across studies. This variability was examined in subgroup analyses (≤ 1 year vs. > 1 year), which did not reveal any meaningful subgroup differences.

Fifth, the certainty of evidence was rated as moderate to very low in the GRADE assessment, primarily due to wide confidence intervals, wide prediction intervals, and inconsistent effect directions. In addition, the risk of bias was partly high: non-randomized studies often relied on preference-based allocation, which resulted in baseline differences (e.g., higher anxiety or greater psychosocial burden at baseline), introducing confounding and selection bias. In randomized trials, blinding of participants and outcome assessors was difficult or impossible to implement, and several studies suffered from incomplete follow-up data, further reducing overall certainty.

Sixth, it should be noted that the pattern of comparatively better outcomes of home treatment at follow-up in psychosocial functioning may partly reflect methodological factors. Follow-up assessments in clinical studies frequently involve substantial rates of missing data. In several of the studies contributing to the analysis of follow-up outcomes in psychosocial functioning (Mattejat et al., 2001; Schmidt et al., 2006), missing follow-up data were handled using complete-case analyses, meaning that only participants with available follow-up data were included in the analyses. Except for Graf et al. (2024), no statistical approaches for handling missing data (e.g., imputation procedures or model-based corrections) were applied when analyzing psychosocial functioning outcomes at follow-up. Consequently, the results may be susceptible to attrition bias.

Seventh, the included studies were conducted exclusively in high-income countries of the Global North. This may limit generalizability, as family structures and the role of social support differ substantially across cultural contexts. In many regions of the Global South, family systems play a more central role in mental health care, which may influence the effectiveness of home-based interventions [66].

### Implications

 Due to methodological limitations, the findings of this review should be interpreted cautiously and primarily provide a preliminary orientation for clinicians as well as a basis for future research. For clinical decision-making, the observed equivalence of both treatment approaches indicates that home treatment may be a viable alternative to inpatient treatment, especially when a less restrictive environment and strong family involvement are beneficial for more sustainable improvements in psychosocial functioning.

To provide more precise recommendations on when HT or IT should be prioritized, future meta-analyses would benefit from structuring outcomes into clinically more nuanced domains. This would allow for the identification of potential differential effects across patient subgroups, for example, by distinguishing between internalizing and externalizing symptoms within psychopathology, or specific school and social functioning within the broader domain of psychosocial functioning. Furthermore, research should differentiate more clearly between stand-alone and sequential home treatment models, as treatment effects may vary depending on service structure. Although many studies exclude children and adolescents with acute self-harm risk, existing evidence suggests that HT combined with adequate safety protocols may reduce suicide attempts [57] and repeated self-injury [58]. Research in this high-risk subgroup remains ethically and practically challenging but would be highly valuable.

To clarify whether HT effectively mitigates the “revolving door” effect, a more differentiated assessment of readmissions is required. In the included studies, this outcome was often only treated as a secondary measure. Future research should prioritize frequency, time-to-event analyses, and distinctions between planned and unplanned admissions, supported by longer follow-up periods. Regional care structures, service capacity, and aftercare quality should also be considered, as they significantly influence readmission patterns. 

This should be pursued through methodologically robust, preferably multicenter RCTs with standardized outcomes and, where possible, blinded assessments. Outcome reporting should be stratified by rater perspective and include long-term follow-up. As demonstrated in adult psychiatry (e.g., the AKtiV trial [67]), large-scale multicenter trials- including children and adolescents- are needed to generate generalizable evidence, particularly in child and adolescent populations, where family and systemic factors play a central role.

Preliminary evidence suggests that certain home treatment models may be more cost-effective than inpatient treatment. However, this evidence is limited and primarily derives from a small number of individual studies evaluating specific intervention models, such as MST, SDS, and HoT-BITs (Hometreatment brings inpatient-treatment outside) [53, 54, 56].

## Supplementary Information

Below is the link to the electronic supplementary material.

Supplementary Material 1

Supplementary Material 2

Supplementary Material 3

Supplementary Material 4

Supplementary Material 5

Supplementary Material 6

Supplementary Material 7

Supplementary Material 8

Supplementary Material 9

Supplementary Material 10

Supplementary Material 11

Supplementary Material 12

## Data Availability

All data and analysis scripts are available at OSF: [https://osf.io/sz4fh/?view_only=ddf867083de5400cbd0311853f85536c](https:/osf.io/sz4fh/?view_only=ddf867083de5400cbd0311853f85536c); all underlying data supporting the findings are provided in the manuscript and Supplementary Information (SI).

## Abbreviations

5D-Scale: Five-Dimensional Assessment of Social Functioning
5DR Program: 5-Day Residential Program
AAH: Acute At Home
AIPW: Augmented Inverse Probability Weighting
BCFPI: Brief Child and Family Phone Interview
BeZuHG: Behandelt zu Hause gesund werden
CAFAS: Child and Adolescent Functional Assessment Scale
CAMHS: Child and Adolescent Mental Health Services
CBCL: Child Behavior Checklist
CBA: Controlled Before-After Study
CG: Comparison Group
CGAS: Children’s Global Assessment Scale
CI: Confidence Interval
CIS: Children’s Global Assessment Scale – Impairment Scale
CGI: Clinical Global Impression
FACES-III: Family Adaptability and Cohesion Evaluation Scales
FFS: Family Functioning Scale
FP Program: Family Preservation Program
GAF: Global Assessment of Functioning
GSI-BSI: Global Severity Index – Brief Symptom Inventory
HoNOSCA: Health of the Nation Outcome Scales for Children and Adolescents
HoT-BITS: Hometreatment brings inpatient-treatment outside
ICCS: Intensive Community Care Service
ICER: Incremental Cost-Effectiveness Ratio
IG: Intervention Group
IHT: Intensive Home Treatment
IFS: Intensive-Family Service
M: Mean
MEI: Mannheim Parent Interview
MSS: Marburg Symptom Scale
MST: Multisystemic Therapy
NHS: National Health Service
NICE: National Institute for Health and Care Excellence
OR: Odds Ratio
QALY: Quality-Adjusted Life Year
RCT: Randomized Controlled Trial
RoB: Risk-of-Bias tool for randomized trials
ROBINS-I: Risk Of Bias In Non-randomized Studies – of Interventions
RT: Residential Treatment
SCIS: Standardized Client Information System
SD: Standard Deviation
SDQ: Strengths and Difficulties Questionnaire
SDS: Supported Discharge Service
SGKJ: Skala zur Gesamtbeurteilung von Kindern und Jugendlichen / Global Assessment Scale for Children and Adolescents
SHQ: Self-Harm Questionnaire
SMD: Standardized Mean Difference
SSRS: Social Skills Rating System
YRBS: Youth Risk Behavior Survey

## Code Availability

All analysis scripts (R code) used in this study are openly available at OSF and in the SI.
