# Supplementary material for: Efficacy of home treatment and inpatient treatment for children and adolescents in psychiatric crisis: a systematic review and meta-analysis
Source: Eur Child Adolesc Psychiatry. 2026 Jun 1;35(7):2103–27. doi: 10.1007/s00787-026-03060-0 (PMC13427882; doi:10.1007/s00787-026-03060-0)
Supplement: Supplementary file 4 — Supplementary Material 4 [file 787_2026_3060_MOESM4_ESM.pdf]

Article title: Efficacy of home treatment and inpatient treatment for children and adolescents in psychiatric crisis: A systematic review and meta-analysis
Journal: European Child & Adolescent Psychiatry
Authors: Karolina Foremnik, Gaby Sroczynski, Jan Stratil, Marjan Arvandi, Anja Neumann, Barbara Buchberger

Medical Faculty, University of Duisburg-Essen, Germany
Corresponding author (KF) E-Mail: karolina.foremnik@uni-due.de

Search strategies for all databases and registers

PubMed via Medline (last search on 20/04/2024)

Search Components	Search String	Search Results
**1 Patient/ Population: children/ adolescents**	child* or adolesc* OR juvenil* OR pediatric* OR infant* OR jugendlich* OR kleinkind* OR kid OR kids OR youth* OR teenage* OR "young adult*" OR (german [Language]) AND (kind*)	**4.516.611**
	MeSH: (child [Mesh]) OR (adolescent [Mesh]) OR (infant [Mesh]) OR (adolescent behavior [Mesh]) OR (child behavior [Mesh]) OR (young adult [Mesh]) OR (adolescent psychiatry [Mesh])	
**in psychiatric crisis**	"psychiatric cris*" OR "mental health cris*" OR "psychiatric emergenc*" OR "mental health emergenc*" OR "severe mental illness*" OR "severe mental disorder*" OR "severe psychiatric illness*" OR "severe psychiatric disorder*" OR "acute mental illness*" OR "acute mental disorder*" OR "acute psychiatric disorder*" OR "acute psychiatric illness*" OR "suicidal ideation*" OR "attempted suicide*" OR suicid* OR "selfharm*" OR "psychiatrische krise*" OR "psychische krise*" OR suizid* OR selbstverletz* OR "mental health problems" OR "psychiatric disorder*"	**211.788**
	MeSH:(suicide, attempted [Mesh]) OR (suicidal ideation [Mesh]) OR (self-injurious behavior [Mesh])	
**2 Intervention: home- based interventions**	hometreat* OR "home treat*" OR "home therapy" OR "inpatient equivalent" OR "ward equivalent" OR stationsaquivalent* OR StaB OR StaeB OR StäB OR "aufsuchende behandlung*" OR mst OR "multisystemic treat*" OR "multisystemic therap*" OR multisystemisch* OR multisystemic OR multi- systemic OR "home care" OR "intensive community treat*" OR "crisis resolution team*" OR "crisis team*" OR CRT OR "family centered treat*" OR FCT OR "assertive community treat*" OR "assertive outreach" OR "mobile treat*" OR "alternative to hospitalization" OR "alternative to hospitalisation" OR "discharge support" OR "discharge service*" OR "intensive support service*" OR "psychiatrische krisenintervention*" OR "case management"	**189.371**
	**MeSH:**(home care service [MeSH Terms]) OR (home care services, hospital based [MeSH Terms]) OR (community mental health centers [MeSH Terms]) OR (community mental health services [MeSH Terms]) OR (mobile health units [Mesh]) OR (community mental health services [Mesh]) OR (crisis intervention [Mesh]) OR (emergency services, psychiatric [Mesh])	

Medical Faculty, University of Duisburg-Essen, Germany
Corresponding author (KF) E-Mail: karolina.foremnik@uni-due.de

**3 <u>Comparison/ Control:</u> Inpatient psychiatric treatment**	"inpatient treat*" OR "psychiatric hospitalization" OR "psychiatric hospitalisation" OR "mental health hospitalization" OR "mental health hospitalisation" OR "psychiatric inpatient care" OR "inpatient mental health care" OR "psychiatric admission*" OR "psychiatric ward*" OR "psychiatric unit*" OR "psychiatric facilit*" OR "psychiatric hospital*" OR "psychiatric clinic*" OR "mental health hospital*" OR "mental health clinic*" OR "mental health facilit*" OR "mental health unit*" OR stationär* OR stationar* OR stationaer*	**264.418**
	**MeSH:** (inpatients [MeSH Terms]) OR (child psychiatry [MeSH Terms]) OR (adolescent psychiatry [MeSH Terms]) OR ("emergency services, psychiatric"[Mesh]) OR (hospitals, psychiatric [Mesh]) OR (mental health services [Mesh])	
1 AND 2 AND 3		**1.453**
limit to (english or german)		**<u>1.348</u>**
MeSH= Medical Subject Headings<br>*: Truncation (variable number of characters)		

PsycINFO via Ovid (last search on 11/05/2024)

**Search Components**	**Search String**	**Search Results**
**1 <u>Patient/ Population</u> children/ adolescents**	child* OR adolescen* OR juvenil* OR pediatric* OR infant* OR jugendlich* OR kleinkind* OR kid? OR youth* OR teenage* OR “young adult*” OR (kind.af. and german.lg)	**1.422.499**
	**Mesh:** exp child psychiatry/ OR exp adolescent psychiatry/ OR exp child behavior/ OR exp adolescent behavior/ OR exp adolescent health/ OR exp early adolescence/ OR exp late adolescence/ OR exp youth mental health/	
**in psychiatric crisis**	“psychiatric cris#s” OR “mental health cris#s” OR “psychiatric emergenc*” OR "mental health emergenc*" OR (severe adj5 “mental illness*”) OR (severe adj5 “mental disorder?”) OR (severe adj5 “psychiatric illness*”) OR (severe adj5 “psychiatric disorder?”) OR (acute adj5 “mental illness*") OR (acute adj5 “mental disorder?") OR (acute adj5 “psychiatric disorder?") OR (acute adj5 “psychiatric illness*”) OR “suicidal ideation?” OR (attempt* adj5 suicide?) OR sucid* OR (self adj1 harm*) OR “psychiatrische krise*” OR “psychische krise*” OR suizid* OR selbstverletz* OR “mental health problem?” OR “psychiatric disorder?”	**352.121**

Medical Faculty, University of Duisburg-Essen, Germany
Corresponding author (KF) E-Mail: karolina.foremnik@uni-due.de

	**Mesh:** exp youth suicide/ OR exp serious mental illness/ OR exp suicide/ OR exp suicidal behavior/ OR exp suicidality/ OR exp suicidal ideation/ OR exp nonsuicidal self-injury/ OR exp self-destructive behavior/ OR exp self-poisoning/ OR exp emotional disturbances/ OR exp antisocial behavior/ OR exp antisocial personality disorder/ OR exp behavior disorders/ OR exp crises/	
**2 <u>Intervention</u> home- based interventions**	hometreat* OR “home treat*” OR “home therapy” OR “inpatient equivalent” OR “ward equivalent” OR stations#quivalent* OR St#B OR “aufsuchende behandlung*” OR mst OR “multisystemic treat*” OR “multisystemic therap*” OR multisystemisch* OR multisystemic OR (multi adj1 systemic) OR “home care” OR “intensive community treat*” OR “crisis resolution team?” OR “crisis team?” OR crt OR “family centered treat*” OR FCT OR “assertive community treat*” OR “assertive outreach” OR “mobile treat*” OR “alternative to hospitali#ation” OR “discharge support” OR “discharge service?” OR “intensive support service?” OR “psychiatrische krisenintervention*” OR “case management”	**50.657**
	**Mesh:** exp home visiting programs/ OR exp community mental health/ OR exp assertive community treatment/ OR exp community mental health centers/ OR exp community psychiatry/ OR exp multisystemic therapy/ OR exp crisis Intervention	
**3 <u>Comparison/ Control</u> Inpatient Psychiatric Treatment**	"inpatient treat*" OR "psychiatric hospitali#ation" OR "mental health hospitali#ation" OR "psychiatric inpatient care" OR "inpatient mental health care" OR "psychiatric admission?" OR "psychiatric ward?" OR "psychiatric unit?" OR "psychiatric facilit*" OR "psychiatric hospital?" OR "psychiatric clinic?" OR "mental health hospital?" OR "mental health clinic?" OR "mental health facilit*" OR "mental health unit?" OR station#r*	**128.401**
	**Mesh:** exp child psychiatry/ OR exp adolescent psychiatry/ OR exp Emergency Services/ OR exp mental health services/	
1 AND 2 AND 3		**1.020**
limit to (english or german)		**<u>984</u>**
*: Truncation (variable number of characters)<br>#: Truncation (exactly one character) ?:<br>Truncation (no or one character) adj*n*:<br>proximity operator		

Article title: Efficacy of home treatment and inpatient treatment for children and adolescents in psychiatric crisis: A systematic review and meta-analysis
Journal: European Child & Adolescent Psychiatry
Authors: Karolina Foremnik, Gaby Sroczynski, Jan Stratil, Marjan Arvandi, Anja Neumann, Barbara Buchberger

Medical Faculty, University of Duisburg-Essen, Germany
Corresponding author (KF) E-Mail: karolina.foremnik@uni-due.de

Embase via Elsevier (last search on 12/05/2024)

**Search Components**	**Search String**	**Search Results**
**1 Patient/ Population** children/ adolescents	child* OR adolescen* OR juvenil* OR pediatric* OR infant* OR jugendlich* OR kleinkind* OR kid$ OR youth* OR teenage* OR "young adult*" OR (kind* AND [german]/lim)	**6.888.948**
	**Emtree:** 'juvenile'/syn OR 'adolescence'/syn OR 'young adult'/syn	
**in psychiatric crisis**	"psychiatric cris?s" OR "mental health cris?s" OR "psychiatric emergenc*" OR "mental health emergenc*" OR (severe NEAR/5 "mental illness*") OR (severe NEAR/5 "mental disorder$") (severe NEAR/5 "psychiatric illness*") OR (severe NEAR/5 "psychiatric disorder$") OR (acute NEAR/5 "mental illness*") OR (acute NEAR/5 "mental disorder$") OR (acute NEAR/5 "psychiatric disorder$") OR (acute NEAR/5 "psychiatric illness*") OR "suicidal ideation$" OR (attempt* NEAR/5 suicide$) OR sucid* OR (self NEAR/1 harm*) OR "psychiatrische krise*" OR "psychiatrische krise*" OR "psychische krise*" OR "psychiatrische krisenintervention*" OR suizid* OR selbstverletz* OR "mental health problem$" OR "psychiatric disorder$"	**182.206**
	**Emtree:** 'suicidal behavior'/syn OR 'automutilation'/syn	
**2 Intervention** home- based interventions	Hometreat* OR "home treat*" OR "home therapy" OR "inpatient equivalent" OR "ward equivalent" OR stations?quivalent* OR St?B OR "aufsuchende behandlung*" OR mst OR "multisystemic treat*" OR "multisystemic therap*" OR multisystemisch* OR multisystemic OR (multi NEAR/1 systemic) OR "home care" OR "intensive community treat*" OR "crisis resolution team$" OR "family centered treat*" OR FCT OR "assertive community treat*" OR "assertive outreach" OR "mobile treat*" OR "alternative to hospitali?ation" OR "discharge support" OR "discharge service$" OR "intensive support service$" OR "psychiatrische krisenintervention*" OR "case management"	**235.267**
	**Emtree:** 'home care'/syn OR 'community mental health service'/syn OR 'community mental health center'/syn OR 'social psychiatry'/syn OR 'crisis intervention'/syn	

Medical Faculty, University of Duisburg-Essen, Germany
Corresponding author (KF) E-Mail: karolina.foremnik@uni-due.de

**3 <u>Comparison/ Control</u> Inpatient Psychiatric Treatment**	"inpatient treat*" OR "psychiatric hospitali?ation" OR "mental health hospitali?ation" OR "psychiatric inpatient care" OR "inpatient mental health care" OR "psychiatric admission$" OR "psychiatric ward$" OR "psychiatric unit$" OR "psychiatric facilit*" OR "psychiatric hospital$" OR "psychiatric clinic$" OR "mental health hospital$" OR "mental health clinic$" OR "mental health facilit*" OR "mental health unit$" OR station?r*	**696.915**
	**Emtree:** 'child psychiatry'/syn OR 'emergency health service'/syn OR 'mental health service'/syn	
1 AND 2 AND 3		**790**
limit to (english or german)		**<u>777</u>**
/syn: Search for the respective EMTREE term including all subheadings and synonyms, additionally conduct a free-text search for all synonyms<br>*: Truncation (variable number of characters)<br>?: Truncation (exactly one character)<br>$: Truncation (no or one character) NEAR/n: proximity operator		

Article title: Efficacy of home treatment and inpatient treatment for children and adolescents in psychiatric crisis: A systematic review and meta-analysis
Journal: European Child & Adolescent Psychiatry
Authors: Karolina Foremnik, Gaby Sroczynski, Jan Stratil, Marjan Arvandi, Anja Neumann, Barbara Buchberger

Medical Faculty, University of Duisburg-Essen, Germany
Corresponding author (KF) E-Mail: karolina.foremnik@uni-due.de

Cochrane via Cochrane library (last search on 15/05/2024)

**Search Components**	**Search String**	**Search Results**
**1 <u>Patient/ Population:</u> children/ adolescents**	child* OR adolescen* OR juvenil* OR pediatric* OR infant* OR jugendlich* OR kleinkind* OR youth OR teenager* OR kid? Or young adult* OR (German:la AND (kind*))	**450.788**
	**MeSH:** [mh child] OR [mh adolescent] OR [mh “young adult”] OR [mh infant] OR [mh youth] OR [mh teenager] OR [mh “adolescent health”] OR [mh “child health”]	
**In psychiatric crisis**	(psychiatric NEAR/5 cris*) OR (mental NEAR/5 cris*) OR (psychiatric NEAR/5 emergenc*) OR (mental NEAR/5 emergenc*) OR ("severe mental" NEAR/5 illness*) OR ("severe mental" NEAR/5 disorder*) OR ("severe psychiatric" NEAR/5 illness*) OR ("severe psychiatric" NEAR/5 disorder*) OR ("acute mental" NEAR/5 illness*) OR ("acute mental" NEAR/5 disorder*) OR ("acute psychiatric" NEAR/5 disorder*) OR ("acute psychiatric" NEAR/5 illness*) OR (suicidal NEAR/1 ideation*) OR (attempt* NEAR/1 suicide*) OR suicid* OR (self NEAR/1 harm*) OR (psychiatrische NEAR/1 krise*) OR (psychische NEAR/1 krise*) OR suizid* OR selbstverletz* OR ("mental health" NEAR/5 problem*) OR (psychiatric NEAR/5 disorder*)	**119.627**
	MeSH: [mh suicide] OR [mh "mental disorder"] OR [mh "selfinjurious behavior"]	

Medical Faculty, University of Duisburg-Essen, Germany
Corresponding author (KF) E-Mail: karolina.foremnik@uni-due.de

**2 Intervention home-based interventions**	hometreat* OR (home NEXT treat*) OR "home therapy" OR "inpatient equivalent" OR "ward equivalent" OR stations?quivalent* OR St?B OR (aufsuchende NEXT behandlung*) OR mst OR (multisystemic NEXT treat*) OR (multisystemic NEXT therap*) OR multisystemisch* OR multisystemic OR (multi NEXT systemic) OR "home care" OR ("intensive community" NEXT treat*) OR ("crisis resolution" NEXT team*) OR ("family centered" NEXT treat*) OR FCT OR ("assertive community" NEXT treat*) OR "assertive outreach" OR (mobile NEXT treat*) OR "alternative to hospitalisation" OR "alternative to hospitalization" OR "discharge support" OR (discharge NEXT service*) OR ("intensive support" NEXT service*) OR (psychiatrische NEXT krisenintervention*) OR "case management"	**14.111**
	MeSH: [mh "home care"] OR [mh "community mental health centers"] OR [mh "community mental health services"] OR [mh "crisis intervention"] OR [mh "community psychiatry"] OR [mh "mobile health units"] OR [mh "emergency services, psychiatric"]	
**3 Comparison/ Control Inpatient Psychiatric Treatment**	(inpatient NEAR/5 treat*) OR (psychiatr* NEAR/5 hospitali?ation) OR inpatient* OR psychiatr* OR ("mental health" NEAR/5 hospitali?ation) OR "psychiatric inpatient care" OR "inpatient mental health care" OR (psychiatric NEAR/5 admission*) OR (psychiatric NEXT ward*) OR (psychiatric NEXT unit*) OR (psychiatric NEXT facilit*) OR (psychiatric NEXT hospital*) OR (psychiatric NEXT clinic*) OR ("mental health" NEXT hospital*) OR ("mental health" NEXT clinic*) OR ("mental health" facilit*) OR ("mental health" unit*) OR station?r*	**116.629**

	[mh inpatients] OR [mh "child psychiatry"] OR [mh "adolescent psychiatry"] OR [mh "emergency services, psychiatric "] OR [mh "hospitals, psychiatric"] OR [mh "mental health services"] OR [mh psychiatry]	
1 AND 2 AND 3		**949**
limit to (english or german)		**939**
MeSH= Medical Subject Headings<br>*: Truncation (variable number of characters) ?:<br>Truncation (exactly one character)<br>NEAR/n and NEXT: Proximity operators		

Article title: Efficacy of home treatment and inpatient treatment for children and adolescents in psychiatric crisis: A systematic review and meta-analysis
Journal: European Child & Adolescent Psychiatry
Authors: Karolina Foremnik, Gaby Sroczynski, Jan Stratil, Marjan Arvandi, Anja Neumann, Barbara Buchberger Medical Faculty,

University of Duisburg-Essen, Germany
Corresponding author (KF) E-Mail: karolina.foremnik@uni-due.de

Electronic grey literature

GoogleScholar (last search on 11/05/2024)

**Search Components**	**Search String**
**1 <u>Patient/ Population:</u> children/ adolescents**	child OR adolescent OR adolescence OR juvenile OR pediatric OR infant OR jugendlich OR kleinkind OR youth OR teenager OR young adult OR adolescent health OR child health
**In psychiatric crisis**	"psychiatric crisis" OR "mental crisis" OR "psychiatric emergency" OR "mental emergency" OR ("severe mental" AROUND(5) illness) OR ("severe mental" AROUND(5) disorder) OR ("severe psychiatric" AROUND(5) illness) OR ("severe psychiatric" AROUND(5) disorder) OR ("acute mental" AROUND(5) illness) OR ("acute mental" AROUND(5) disorder) OR ("acute psychiatric" AROUND(5) disorder) OR ("acute psychiatric" AROUND(5) illness) OR OR “suicidal ideation” OR “attempted suicide” OR “suicide attempt” OR suicide OR suicidal OR “self harm” OR "psychiatrische krise" OR "psychische krise" OR suizid OR selbstverletzung OR ("mental health" AROUND(5) problem) OR ("psychiatric" AROUND(5) disorder)
**2 <u>Intervention</u> home-based interventions**	hometreat OR "home treatment" OR "home therapy" OR "inpatient equivalent" OR "ward equivalent" OR stationsequivalent OR StB OR "aufsuchende behandlung" OR mst OR "multisystemic treatment" OR "multisystemic therapy" OR multisystemisch OR multisystemic OR "multi systemic" OR "home care" OR "intensive community treatment" OR "crisis resolution team" OR "family centered treatment" OR FCT OR "assertive community treatment" OR "assertive outreach" OR "mobile treatment" OR "alternative to hospitalisation" OR "alternative to hospitalization" OR "discharge support" OR "discharge service" OR "intensive support service" OR "psychiatrische krisenintervention" OR "case management" OR crisis intervention
**3 <u>Comparison/ Control</u> Inpatient Psychiatric Treatment**	"inpatient treatment" AROUND(5) psychiatric OR (psychiatric AROUND(5) hospitalization) OR (psychiatric AROUND(5) hospitalisation) OR inpatient OR psychiatric OR ("mental health" AROUND(5) hospitalization) OR ("mental health" AROUND(5) hospitalisation) OR "psychiatric inpatient care" OR "inpatient mental health care" OR (psychiatric AROUND(5) admission) OR "psychiatric ward" OR "psychiatric unit" OR "psychiatric facility" OR "psychiatric hospital" OR "psychiatric clinic" OR "mental health hospital" OR "mental health clinic" OR "mental health facility" OR "mental health unit" OR stationär
1 AND 2 AND 3	screening of the first 50 hits
AROUND (n): Proximity operators	

ERIC (last search on 11/05/2024)

**Search Components**	**Search String**

Article title: Efficacy of home treatment and inpatient treatment for children and adolescents in psychiatric crisis: A systematic review and meta-analysis
Journal: European Child & Adolescent Psychiatry
Authors: Karolina Foremnik, Gaby Sroczynski, Jan Stratil, Marjan Arvandi, Anja Neumann, Barbara Buchberger Medical Faculty,
University of Duisburg-Essen, Germany
Corresponding author (KF) E-Mail: karolina.foremnik@uni-due.de

**1 Patient/ Population: children/ adolescents**	children OR adolescence OR juvenile OR pediatric OR infant OR jugendlich OR kleinkind OR youth OR teenager OR “young adult” OR “adolescent health” OR “child health”
**In psychiatric crisis**	“Psychiatric crisis” OR “mental crisis” OR “psychiatric emergency” OR “mental emergency” OR "severe mental" OR "severe psychiatric" OR "acute mental" OR "acute mental" OR "acute psychiatric" OR suicide OR suicidal OR self-harm OR “psychiatrische krise” OR “psychische krise” OR suizid OR selbstverletzung OR “psychiatric disorder”
**2 Intervention home-based interventions**	hometreatment OR home treatment OR "home therapy" OR "inpatient equivalent" OR "ward equivalent" OR stationsäquivalent OR StäB OR “aufsuchende behandlung” OR mst OR multisystemic OR multisystemisch OR "home care" OR “intensive community treatment” OR “crisis resolution” OR “family centered treatment” OR FCT OR “assertive community treatment” OR "assertive outreach" OR “mobile treatment” OR "alternative to hospitalisation" OR "alternative to hospitalization" OR "discharge support" OR “discharge service” OR “intensive support service” OR “psychiatrische krisenintervention” OR "case management" OR crisis intervention
**3 Comparison/ Control Inpatient Psychiatric Treatment**	“inpatient treatment” OR hospitalisation OR hospitalization OR hospital OR inpatient OR psychiatry OR “psychiatric hospital” OR “psychiatric clinic” OR stationär*
1 AND 2 AND 3	screening of the first 50 hits
*: Truncation (variable number of characters)<br>NEAR/n: Proximity operators	

Registers
ClinicalTrials.gov (last search on 30/05/2024)

	Search Components	Search String
	**1 Patient/ Population: children/ adolescents**	children OR adolescence OR juvenile OR pediatric OR infant OR jugendlich OR kleinkind OR youth OR teenager OR “young adult” OR “adolescent health” OR “child health”
	**In psychiatric crisis**	"psychiatric crisis" OR "mental health crisis" OR

Article title: Efficacy of home treatment and inpatient treatment for children and adolescents in psychiatric crisis: A systematic review and meta-analysis
Journal: European Child & Adolescent Psychiatry
Authors: Karolina Foremnik, Gaby Sroczynski, Jan Stratil, Marjan Arvandi, Anja Neumann, Barbara Buchberger Medical Faculty, University of Duisburg-Essen, Germany
Corresponding author (KF) E-Mail: karolina.foremnik@uni-due.de

**Condition/ disease**		"psychiatric emergency" OR "mental health emergency" OR "severe mental illness" OR "severe mental disorder" OR "severe psychiatric illness" OR "severe psychiatric disorder" OR "acute mental illness" OR "acute mental disorder" OR "acute psychiatric disorder" OR "acute psychiatric illness" OR "suicidal ideation" OR "attempted suicide" OR suicide OR self-harm OR "psychiatrische krise" OR "psychische krise" OR suizid OR selbstverletzung OR "mental health problem" OR "psychiatric disorder"
**Intervention/ treatment**	**2 Intervention**<br>**home- based interventions**	hometreatment OR home treatment OR "home therapy" OR "inpatient equivalent" OR "ward equivalent" OR stationsäquivalent OR StäB OR "aufsuchende behandlung" OR mst OR multisystemic OR multisystemisch OR "home care" OR "intensive community treatment" OR "crisis resolution" OR "family centered treatment" OR FCT OR "assertive community treatment" OR "assertive outreach" OR "mobile treatment" OR "alternative to hospitalisation" OR "alternative to hospitalization" OR "discharge support" OR "discharge service" OR "intensive support service" OR "psychiatrische krisenintervention" OR "case management" OR crisis intervention
	**3 Comparison/ Control**<br>**Inpatient Psychiatric Treatment**	"inpatient treatment" OR hospitalisation OR hospitalization OR hospital OR inpatient OR psychiatry OR "psychiatric hospital" OR "psychiatric clinic" OR stationär
	1 AND 2 AND 3	**Search results: 223**

International Clinical Trials Registry Platform (ICTRP) (last search on 30/05/2024)

**Search Components**	**Search String**
**1 Patient/ Population: children/ adolescents**	Filter: "Search for clinical trials in children"
**In psychiatric crisis**	No keywords

Authors: Karolina Foremnik, Gaby Sroczynski, Jan Stratil, Marjan Arvandi, Anja Neumann, Barbara Buchberger Medical Faculty, University of Duisburg-Essen, Germany
Corresponding author (KF) E-Mail: karolina.foremnik@uni-due.de

**2 Intervention**<br>**home- based interventions**	hometreat* OR home treat* OR home therapy OR inpatient equivalent OR ward equivalent OR stationsaequivalent OR StaeB OR aufsuchende behandlung OR mst OR multisystemic OR multisystemisch OR home care OR intensive community treatment OR crisis resolution OR family centered treatment OR FCT OR assertive community treatment OR assertive outreach OR mobile treatment OR alternative to hospitalisation OR alternative to hospitalization OR discharge support OR discharge service* OR intensive support service* OR psychiatrische krisenintervention* OR case management OR crisis intervention AND home
**3 Comparison/ Control**<br>**Inpatient Psychiatric Treatment**	No keywords
1 AND 2	**Search results: 73**
*: Truncation (variable number of characters)	
