# Supplementary material for: Efficacy of home treatment and inpatient treatment for children and adolescents in psychiatric crisis: a systematic review and meta-analysis
Source: Eur Child Adolesc Psychiatry. 2026 Jun 1;35(7):2103–27. doi: 10.1007/s00787-026-03060-0 (PMC13427882; doi:10.1007/s00787-026-03060-0)
Supplement: Supplementary file 5 — Supplementary Material 5 [file 787_2026_3060_MOESM5_ESM.pdf]

Article title: Efficacy of home treatment and inpatient treatment for children and adolescents in psychiatric crisis:
A systematic review and meta-analysis

Journal: European Child & Adolescent Psychiatry
Authors: Karolina Foremnik, Gaby Sroczynski, Jan Stratil, Marjan Arvandi, Anja Neumann, Barbara Buchberger

Medical Faculty, University of Duisburg-Essen, Germany

Corresponding author (KF) E-Mail: karolina.foremnik@uni-due.de

Title	Study Design	Author	Year	Country	Status	Reason for exclusion
One-Year Follow-up of Multisystemic Therapy as an Alternative to the Hospitalization of Youths in Psychiatric Crisis	RCT	Henggeler et al.	2003	USA	Excluded for quantative synthesis	overlapping samples with more complete publications
Treatment outcome of an intensive psychiatric home treatment for children and adolescents: a non-randomized controlled pilot evaluation	non-randomized controlled pilot evaluation	Graf et al.	2023	Switzerland	Excluded for quantative synthesis	overlapping samples with more complete publications
Evaluation of Home Treatment for Children and Adolescents with Acute Psychiatric Disorders/ Kaess	Trial	Kaess	2020	Switzerland	Excluded after full-text assessment	(Ongoing) trials with no results
Treatment costs for youths receiving multisystemic therapy or hospitalization after a psychiatric crisis/ Sheidow et al	RCT	Sheidow et al.	2004	USA	Excluded for quantative synthesis	overlapping samples with more complete publications
Supported discharge vs In-patient Treatment Evaluation (SITE)/	RCT (Trial)	Ougrin et al.	2021	UK	Excluded after full-text assessment	(Ongoing) trials with no results
Impact of Mobile Crisis Services on Emergency Department Use Among Youths With Behavioral Health Service Needs/	non- RCT	Fendrich et al.	2019	USA	Excluded after full-text assessment	no/ wrong control intervention
Cost-effectiveness of intensive home treatment enhanced by inpatient treatment elements in child and adolescent psychiatry in Germany: a randomized trial.	RCT	Boege et al.	2015	Germany	Excluded for quantative synthesis	overlapping samples with more complete publications
Family Health and Development Project: Intensive In-home Child and Adolescent Psychiatric Service (IICAPS) vs. Home-based Child Treatment Coordination (Home-based CTC) for Seriously Emotionally Disturbed Children	RCT (Trial)	Woolston et al.	2012	USA	Excluded after full-text assessment	no/ wrong control intervention
Inpatient Care Utilization Following Mobile Crisis Response Encounters Among Racial/Ethnic Minoritized Youth	uncontrolled before and after study	Lui et al.	2024	USA	Excluded after full-text assessment	No or non-extractable outcome data
Crisis Intervention Program: An Alternative to Inpatient Psychiatric Treatment for Children	uncontrolled before and after study	Blumberg	2002	USA	Excluded after full-text assessment	wrong study design
Pilot Study of the Cost-Effectiveness of Youth F-ACT in the Netherlands	non- RCT	Hodgewind et al.	2021	Netherlands	Excluded after full-text assessment	no/ wrong control intervention
Non-suicidal self-injury and suicidal behaviour in children and adolescents accessing residential or intensive home-based mental health services.	longitudinal, observational (no CG)	Preyde et al.	2012	Canada	Excluded after full-text assessment	wrong study design
Long-term outcomes of children and youth accessing residential or intensive home-based treatment: Three year follow up.		Preyde et al.	2011	Canada	Excluded after full-text assessment	no/ wrong control intervention
Development and implementation of an experimental study of the effectiveness of intensive in-home crisis services for children and their families.	RCT (methodological study)	Evans et al.	1997	USA	Excluded after full-text assessment	no/ wrong control intervention
An experimental study of the effectiveness of intensive in-home crisis services for children and their families: Program outcomes/ Evans et al./ 2003	RCT	Evans et al.	2003	USA	Excluded after full-text assessment	no/ wrong control intervention
Outcomes for youth receiving intensive in-home therapy or residential care: A comparison using propensity scores.	non- RCT (with Propensity Score Matching	Barth et al.	2007	USA	Excluded after full-text assessment	wrong intervention
Intensive home treatment for adolescents in psychiatric crisis.	longitudinal observational (no CG)	Muskens et al.	2019	Netherlands	Excluded after full-text assessment	wrong study design
Home vs hospital care of children with behavior disorders. A controlled investigation	RCT	Winsberg et al.	1980	USA	Excluded after full-text assessment	wrong intervention
Treatment programs for youth with emotional and behavioral disorders: An outcome study of two alternate approaches.		Wilmshurst et al.	2002	USA	Excluded after full-text assessment	no/ wrong control intervention
Outcomes of children participating in mental health residential and intensive family services: Preliminary findings		Preyde et al.	2009	Canada	Excluded after full-text assessment	no/ wrong control intervention
Trajectories of symptom reduction and engagement during treatment for childhood behavior disorders: differences across settings	RCT	Lindhiem et al.	2010	USA	Excluded after full-text assessment	no/ wrong control intervention
Therapy evaluation in child and adolescent psychiatry: inpatient treatment, day care treatment and home treatment in comparison]	RCT	Remschmidt et al.	1988	Germany	Excluded after full-text assessment	No or non-extractable outcome data
The cost of treatment of psychiatric emergencies: a comparison of hospital and community services	RCT	Merson et al.	1996	UK	Excluded after full-text assessment	wrong population
Outcomes associated with clinical profiles of children in psychiatric crisis enrolled in intensive, in-home interventions.	RCT	Evans et al.	2001	USA	Excluded after full-text assessment	no/ wrong control intervention
Comparison of community based service with hospital based service for people with acute, severe psychiatric illness.	non- RCT	Dean et al.	1993	UK	Excluded after full-text assessment	wrong population
Randomised controlled trial of acute mental health care by a crisis resolution team: the north Islington crisis study.	RCT	Johnson et al.	2005	UK	Excluded after full-text assessment	wrong population
Multisystemic therapy as community-based treatment for youth with severe emotional disturbance.	Controlled before and after study	Painter et al,	2009	USA	Excluded after full-text assessment	no/ wrong control intervention
A controlled trial of home-based acute psychiatric services. I: Clinical and social outcome	RCT	Burns et al.	1993	UK	Excluded after full-text assessment	wrong population

Randomised comparison of the effectiveness and costs of community and hospital based mental health services for children with behavioural disorders	RCT	Harrington et al.	2000	UK	Excluded after full-text assessment	wrong population
Effectiveness of nonresidential specialty mental health services for children and adolescents in the "real world"	longitudinal observational	Angold et al.	2000	USA	Excluded after full-text assessment	no/ wrong control intervention
Specialized home treatment versus hospital-based outpatient treatment for first-episode psychosis: a randomized clinical trial	RCT	Dewa et al.	2009	Canada	Excluded after full-text assessment	wrong population
Community vs. clinic-based modular treatment of children with early-onset ODD or CD: a clinical trial with 3-year follow-up	RCT	Kolko et al.	2009	USA	Excluded after full-text assessment	no/ wrong control intervention
A comparative trial of home and hospital psychiatric care. One-year follow-up	RCT	Fenton et al.	1979	Canada	Excluded after full-text assessment	wrong population
Effectiveness of home treatment in children and adolescents with externalizing psychiatric disorders.	uncontrolled before and after	Lay et al.	2001	Germany	Excluded after full-text assessment	no/ wrong control intervention
Evaluation of wraparound services for severely emotionally disturbed youths	non- RCT	Mears et al.	2009	USA	Excluded after full-text assessment	wrong population
Heavy users of acute psychiatric beds: randomized controlled trial of enhanced community management in an outer London borough	RCT	Harrison et al.	2002	UK	Excluded after full-text assessment	wrong population
Costs and benefits of hospital and day treatment with community care of affective and schizophrenic disorders	RCT	Wiersma et al.	1995	Netherlands	Excluded after full-text assessment	wrong population
Home-based versus hospital-based care for people with serious mental illness.	RCT	Marks et al.	1994	USA	Excluded after full-text assessment	wrong population
In-home treatment of families with seriously disturbed adolescents in crisis.	uncontrolled before and after	Seelig et al.	1992	USA	Excluded after full-text assessment	wrong study design
A randomised multicentre trial of integrated versus standard treatment for patients with a first episode of psychotic illness	RCT	Petersen et al.	2005	Denmark	Excluded after full-text assessment	wrong population
Home-based versus hospital-based care for serious mental illness. Controlled cost-effectiveness study over four years/ Knapp/ 1998	RCT	Knapp et al.	1998	UK	Excluded after full-text assessment	wrong population
Home-based versus out-patient/in-patient care for people with serious mental illness: Phase II of a controlled study.	RCT	Audini er al.	1994	UK	Excluded after full-text assessment	wrong population
A controlled trial of home-based acute psychiatric services. II: treatment patterns and costs	RCT	Burns et al.	1993	UK	Excluded after full-text assessment	wrong population
Service use and costs of home-based versus hospital-based care for people with serious mental illness	RCT	Knapp et al.	1998	UK	Excluded after full-text assessment	wrong population
Multisystemic Therapy (MST) for youths with severe conduct disorders - economic evaluation of the implementation in a German-speaking environment	non- RCT	Rehberg et al.	2011	Switzerland	Excluded after full-text assessment	No or non-extractable outcome data
Evaluation of Intensive Community Care Services for young people with psychiatric emergencies: study protocol for a multi-centre parallel-group, single-blinded randomized controlled trial with an internal pilot phase.	RCT (study protocol)	Thaventhiran et al.	2024	UK	Excluded after full-text assessment	(Ongoing) trials with no results
Child, family, and system outcomes of intensive case management in New York State	uncontrolled before and after	Evans et al.	1996	USA	Excluded after full-text assessment	wrong intervention
Effectiveness of Intensive Alternative Family Treatment on Reducing Re-Admissions to Psychiatric Residential Treatment Facilities	non- RCT	Rose et al.	2021	USA	Excluded after full-text assessment	wrong intervention
A New Acute-At-Home Child and Adolescent Clinical Service: Evaluation of Impact	Controlled before and after study	Khalfan et al.	2022	Canada	Excluded after full-text assessment	No or non-extractable outcome data
Home-Based Treatment, Rates of Ambulatory Follow-Up, and Psychiatric Rehospitalization in a Medicaid Managed Care Population	uncontrolled before and after	Frederick et al.	2002	USA	Excluded after full-text assessment	no/ wrong control intervention
Systemic Crisis Intervention as a Response to Adolescent Crises: An Outcome Study	RCT	Gutstein et al.	1988	USA	Excluded after full-text assessment	wrong intervention
Occupied bed days a redundant currency? An evaluation of the first 10 years of an integrated model of care for mentally ill adolescents.	Controlled before and after study	Adrian et al.	2015	UK	Excluded after full-text assessment	no/ wrong control intervention
Intensive home treatment of adolescents in crisis: treat the parents along with 'psychiatric' adolescents	uncontrolled before and after	Muskens et al.	2015	Netherlands	Excluded after full-text assessment	wrong study design
An Efficacy Study of a Youth Mobile Crisis Intervention Program	non- RCT	Martin	2005	USA	Excluded after full-text assessment	no/ wrong control intervention
Mental Health Outcomes of Children and Youth Accessing Residential Programs or a Home-Based Alternative					Excluded after full-text assessment	no/ wrong control intervention
		Preyde et al.	2010	Canada		
A Home-Treatment System in Child and Adolescent Psychiatry	controlled before and after study	Erkohlati	2004	Finnland	Excluded after full-text assessment	wrong study design
Modelling the Clinical and Economic Impacts of Foundation-Funded versus Staff-Driven Quality Improvement Mental Health Strategies	controlled before and after study	McCaffrey/ Cawthorpe	2023	Canada	Excluded after full-text assessment	No or non-extractable outcome data
