# Supplementary material for: Efficacy of home treatment and inpatient treatment for children and adolescents in psychiatric crisis: a systematic review and meta-analysis
Source: Eur Child Adolesc Psychiatry. 2026 Jun 1;35(7):2103–27. doi: 10.1007/s00787-026-03060-0 (PMC13427882; doi:10.1007/s00787-026-03060-0)
Supplement: Supplementary file 6 — Supplementary Material 6 [file 787_2026_3060_MOESM6_ESM.pdf]

Author(s):
Question: Hometreatment compared to inpatient treatment (TAU) for children and adolescents in psychiatric crises
Setting:
Bibliography:

Certainty assessment							№ of patients		Effect		Certainty	Importance
**№ of studies**	**Study design**	**Risk of bias**	**Inconsistency**	**Indirectness**	**Imprecision**	**Other considerations**	**Hometreatment**	**inpatient treatment (TAU)**	**Relative (95% CI)**	**Absolute (95% CI)**		
**Psychopathology post- stand alone models (assessed with: various measures (e.g. GSI-BSI, HoNOSCA)**												
5	non-randomised studies	serious[a]	serious[b]	not serious	serious[c]	none	189	171	-	SMD **0.23 SD lower** (0.77 lower to 0.3 higher)	⊕◯◯◯ Very low[a,b,c]	CRITICAL
**Psychopathology follow up- stand alone models (follow-up: range 12 months to 48 months; assessed with: (various measures (e.g. GSI-BSI,HoNOSCA))**												
5	non-randomised studies	serious[a]	not serious[d]	not serious	serious[c]	none	199	187	-	SMD **0.05 SD higher** (0.18 lower to 0.27 higher)	⊕⊕◯◯ Low[a,c,d]	CRITICAL
**Psychosocial functioning post- stand alone models (assessed with: various measures (e.g. CBCL, GAF)**												
4	non-randomised studies	serious[a]	not serious[d]	not serious	serious[c]	none	162	124	-	SMD **0.27 SD lower** (0.8 lower to 0.26 higher)	⊕⊕◯◯ Low[a,c,d]	CRITICAL
**Psychosocial functioning follow up- stand alone models (follow-up: range 12 months to 48 months; assessed with: various measures (e.g. CBCL, GAF)**												
4	non-randomised studies	serious[a]	not serious[d]	not serious	serious[c]	none	121	109	-	SMD **0.31 SD higher** (0.04 lower to 0.66 higher)	⊕⊕◯◯ Low[a,c,d]	CRITICAL
**Psychopathology post- sequential (assessed with: SDQ, HoNOSCA)**												
2	randomised trials	not serious	serious[e]	not serious	serious[c]	none					⊕⊕◯◯ Low[c,e]	IMPORTANT
**Psychopathology follow-up- sequential (follow-up: range 6 months to 8.4 months; assessed with: SDQ, HoNOSCA)**												
2	randomised trials	not serious	not serious	not serious	serious[c]	none					⊕⊕⊕◯ Moderate[c]	IMPORTANT
**Psychosocial functioning post- sequential (assessed with: CGAS, CIS)**												
1	randomised trials	not serious	not serious	not serious	serious[c]	none					⊕⊕⊕◯ Moderate[c]	CRITICAL
**Psychosocial functioning follow-up- sequential (follow-up: range 6 months to 6 months; assessed with: CGAS)**												
1	randomised trials	not serious	not serious	not serious	serious[c]	none					⊕⊕⊕◯ Moderate[c]	CRITICAL
**Family functioning pre/post and follow up (follow-up: range 12 months to 12 months; assessed with: FACES-III, FFS)**												
3	non-randomised studies	serious[a]	serious[b]	not serious	serious[c]	none	Given the limited evidence base (k=3; two studies based on the same population: Huey et al. 2004 and Henggeler et al. 1999), study-level effects are presented narratively without pooling.<br>At post-treatment, results varied across domains and rater perspectives (Fig. 10). Statistically significant effects were observed for parent-rated cohesion (g = 0.52, 95% CI [0.15, 0.90]), self-rated adaptability (g = 0.41, 95% CI [0.04, 0.78]), and parent-rated control (g = 0.32, 95% CI [0.00, 0.63]), indicating better outcomes for the intervention. One significant effect favoring the comparator was observed for parent-rated general family functioning (g = -0.42, 95% CI [-0.83, -0.01]).<br>At follow-up, none of the effects across domains and raters were statistically significant				⊕◯◯◯ Very low[a,b,c]	IMPORTANT
**Readmission Intervention and Control group- stand alone (follow-up: range 3.5 months to 21 months)**												
3	non-randomised studies	serious[a]	not serious	not serious	serious[c]	none			**RR 1.41** (0.98 to 2.01)	**1 fewer per 1.000** (from 2 fewer to 1 fewer)	⊕⊕◯◯ Low[a,c]	IMPORTANT
**Readmission Intervention and Control group- hybrid (follow-up: range 6 months to 52 months)**												

2	randomised trials	not serious	not serious	not serious	very serious[c,f]	none			not estimable		⨁⨁◯◯ Low[c,f]	IMPORTANT

CI: confidence interval; **RR:** risk ratio; **SMD:** standardised mean difference

Explanations

a. Several studies were at high risk of bias in Domain 4 (measurement of outcomes) because outcome assessors (parents and teachers) were not blinded, which is particularly problematic for subjective outcomes. In addition, some studies allocated participants based on participant or parent preference, introducing confounding and selection bias.
b. Substantial heterogeneity was observed, which could partly be explained through sensitivity analyses. However, this led to a change in the direction of the effect, and only a small number of studies remained included./ The directions of effects were inconsistent, with some significant results favoring the intervention, others favoring the control group, and several showing no effect.
c. The confidence interval crosses the line of no effect and includes both a potential clinically relevant benefit and no effect
d. low to moderate heterogeneity
e. Narrative assessment suggests some inconsistency as effect estimates vary in direction
f. only 2 studies available
