# Supplementary material for: Efficacy of home treatment and inpatient treatment for children and adolescents in psychiatric crisis: a systematic review and meta-analysis
Source: Eur Child Adolesc Psychiatry. 2026 Jun 1;35(7):2103–27. doi: 10.1007/s00787-026-03060-0 (PMC13427882; doi:10.1007/s00787-026-03060-0)
Supplement: Supplementary file 7 — Supplementary Material 7 [file 787_2026_3060_MOESM7_ESM.pdf]

Article title: Efficacy of home treatment and inpatient treatment for children and adolescents in psychiatric crisis: A systematic review and meta-analysis
Journal: European Child & Adolescent Psychiatry
Authors: Karolina Foremnik, Gaby Sroczynski, Jan Stratil, Marjan Arvandi, Anja Neumann, Barbara Buchberger
Medical Faculty, University of Duisburg-Essen, Germany Corresponding author (KF) E-Mail: karolina.foremnik@uni-due.de

Risk of Bias in Non-Randomized Studies (ROBINS-I)

Risk of bias assessment Graf et al., 2023

Signalling questions	Description	Response options
**Bias due to confounding**		
1.1 Is there potential for confounding of the effect of intervention in this study?<br>**If N/PN to 1.1:** the study can be considered to be at low risk of bias due to confounding and no further signalling questions need be considered	**Probably yes:** There is potential for confounding of the effect of intervention, as the group assignment is not random but based on personal preferences.<br><br>Therefore, there was an uneven distribution of participants in the intervention groups (AT_HOME: N= 37; I-TAU: N=97), but no significant group differences for sex, age and baseline HoNOSCA score. Significant group differences occur for GAF score (psychosocial functioning), HoNOSCA-SR score (psychopathological burden) and distribution of principal ICD- diagnoses at baseline (*Patients with anxiety disorders and who feel subjectively healthier are likely to opt for home treatment, whereas those with depression tend to prefer the inpatient setting because of the more structured daily routine*). Also, differences in terms of the size of the average duration of treatment (AT_HOME: 83.73 vs. I-TAU: 100.76 days.	Y / PY / PN / N
**If Y/PY to 1.1**: determine whether there is a need to assess time-varying confounding:		
1.2. Was the analysis based on splitting participants' follow up time according to intervention received?<br>**If N/PN**, answer questions relating to baseline confounding (1.4 to 1.6)<br>**If Y/PY**, go to question 1.3.	**No:** Splitting participants' follow-up time by intervention received was not necessary, as there was no switching between interventions *(while it was considered that patients in the AT_HOME group might require brief hospitalization (up to 3 days) during the treatment period, for instance due to suicidal tendencies, this situation did not occur.)*	NA / Y / PY / PN / N / NI

Authors: Karolina Foremnik, Gaby Sroczynski, Jan Stratil, Marjan Arvandi, Anja Neumann, Barbara Buchberger
Medical Faculty, University of Duisburg-Essen, Germany Corresponding author (KF) E-Mail: karolina.foremnik@uni-due.de

1.3. Were intervention discontinuations or switches likely to be related to factors that are prognostic for the outcome?<br>**If N/PN**, answer questions relating to baseline confounding (1.4 to 1.6)<br>**If Y/PY**, answer questions relating to both baseline and time-varying confounding (1.7 and 1.8)		NA / Y / PY / PN / N / NI

Article title: Efficacy of home treatment and inpatient treatment for children and adolescents in psychiatric crisis: A systematic review and meta-analysis
Journal: European Child & Adolescent Psychiatry
Authors: Karolina Foremnik, Gaby Sroczynski, Jan Stratil, Marjan Arvandi, Anja Neumann, Barbara Buchberger
Medical Faculty, University of Duisburg-Essen, Germany Corresponding author (KF) E-Mail: karolina.foremnik@uni-due.de

**Questions relating to baseline confounding only**		
1.4. Did the authors use an appropriate analysis method that controlled for all the important confounding domains?	**Yes:** Baseline differences due to lack of randomisation and differences in treatment duration were accounted for by augmented inverse probability weights (AIPW).<br><br>Sensitivity power analyses were conducted to justify the unequal sample sizes post hoc (1 - β = 0.8).	NA / Y / PY / PN / N / NI
1.5. If **Y/PY to 1.4**: Were confounding domains that were controlled for measured validly and reliably by the variables available in this study?	**Yes**: The confounding domains controlled for were validly and reliably measured by the variables available in this study:<br>Psychosocial functioning was measured by GAF scores (measured only at admission and not longitudinally.) Self-rated and clinician-rated psychopathological burden was measured by HoNOSCA (SR) scores.	NA / Y / PY / PN / N / NI
1.6. Did the authors control for any post-intervention variables that could have been affected by the intervention?	**No information**	NA / Y / PY / PN / N / NI
**Questions relating to baseline and time-varying confounding**		
1.7. Did the authors use an appropriate analysis method that controlled for all the important confounding domains and for time-varying confounding?	**Yes:** Baseline differences due to lack of randomisation and differences in treatment duration were accounted for by augmented inverse probability weights (AIPW). Time-varying confounding is rather unlikely in this study, as the observation period is relatively short and there is no follow-up	NA / Y / PY / PN / N / NI
1.8. If **Y/PY to 1.7**: Were confounding domains that were controlled for measured validly and reliably by the variables available in this study?	**Yes.** See 1.5.	NA / Y / PY / PN / N / NI
**Risk of bias judgement**	**Moderate risk of bias:** There is potential for confounding due to choice-based allocation. While important confounders were addressed using AIPW, an overrepresentation of patients with anxiety disorders in the HT arm remains. This could influence both treatment choice and outcomes. However, the impact on the main results is likely limited	Low / Moderate / Serious / Critical / NI
Optional: What is the predicted direction of bias due to confounding?		Favours experimental / Favours comparator / Unpredictable

Article title: Efficacy of home treatment and inpatient treatment for children and adolescents in psychiatric crisis: A systematic review and meta-analysis
Journal: European Child & Adolescent Psychiatry
Authors: Karolina Foremnik, Gaby Sroczynski, Jan Stratil, Marjan Arvandi, Anja Neumann, Barbara Buchberger
Medical Faculty, University of Duisburg-Essen, Germany Corresponding author (KF) E-Mail: karolina.foremnik@uni-due.de

**Bias in selection of participants into the study**		
2.1. Was selection of participants into the study (or into the analysis) based on participant characteristics observed after the start of intervention?<br>**If N/PN to 2.1:** go to 2.4	**No:** Selection into the analysis was based on participant availability and consent at follow-up, not on characteristics observed after the start of the intervention.	Y / PY / PN / N / NI
2.2. **If Y/PY to 2.1**: Were the post-intervention variables that influenced selection likely to be associated with intervention?<br>2.3 **If Y/PY to 2.2**: Were the post-intervention variables that influenced selection likely to be influenced by the outcome or a cause of the outcome?		NA / Y / PY / PN / N / NI<br>NA / Y / PY / PN / N / NI
2.4. Do start of follow-up and start of intervention coincide for most participants?	**Yes:** The start of follow-up and the start of intervention coincided for most participants in the study, ensuring a clear temporal relationship between treatment exposure and outcome measurement.	Y / PY / PN / N / NI
2.5. **If Y/PY to 2.2 and 2.3, or N/PN to 2.4**: Were adjustment techniques used that are likely to correct for the presence of selection biases?		NA / Y / PY / PN / N / NI
**Risk of bias judgement**	**Low risk of bias:** All participants who would have been eligible for the target trial were included in the study and for each participant, start of follow up and start of intervention coincided.	Low / Moderate / Serious / Critical / NI
Optional: What is the predicted direction of bias due to selection of participants into the study?		Favours experimental / Favours comparator / Towards null /Away from null / Unpredictable

Article title: Efficacy of home treatment and inpatient treatment for children and adolescents in psychiatric crisis: A systematic review and meta-analysis
Journal: European Child & Adolescent Psychiatry
Authors: Karolina Foremnik, Gaby Sroczynski, Jan Stratil, Marjan Arvandi, Anja Neumann, Barbara Buchberger
Medical Faculty, University of Duisburg-Essen, Germany Corresponding author (KF) E-Mail: karolina.foremnik@uni-due.de

**Bias in classification of interventions**		
3.1 Were intervention groups clearly defined?	**Yes:** the interventions for both AT_Home and I-TAU are clearly defined in terms of type, setting, frequency, intensity, and timing of the intervention.	Y / PY / PN / N / NI
3.2 Was the information used to define intervention groups recorded at the start of the intervention?	**Yes:** The information used to define the two groups was recorded at the start of the intervention.	Y / PY / PN / N / NI
3.3 Could classification of intervention status have been affected by knowledge of the outcome or risk of the outcome?	**Probably yes:** The classification of intervention status was likely affected by knowledge of the risk of the outcome, as clinical and parental decisions regarding treatment group assignment could have been based on factors such as symptom severity and family context.	Y / PY / PN / N / NI
**Risk of bias judgement**	**Moderate risk of bias:** Although intervention status was clearly defined and based on pre-intervention data, the self-selected group assignment may have been influenced by knowledge of the risk of the outcome.	Low / Moderate / Serious / Critical / NI
Optional: What is the predicted direction of bias due to classification of interventions?		Favours experimental / Favours comparator / Towards null /Away from null / Unpredictable

Article title: Efficacy of home treatment and inpatient treatment for children and adolescents in psychiatric crisis: A systematic review and meta-analysis
Journal: European Child & Adolescent Psychiatry
Authors: Karolina Foremnik, Gaby Sroczynski, Jan Stratil, Marjan Arvandi, Anja Neumann, Barbara Buchberger
Medical Faculty, University of Duisburg-Essen, Germany Corresponding author (KF) E-Mail: karolina.foremnik@uni-due.de

**Bias due to deviations from intended interventions**		
**If your aim for this study is to assess the effect of assignment to intervention, answer questions 4.1 and 4.2**		
4.1. Were there deviations from the intended intervention beyond what would be expected in usual practice?	**No:** ´The participants remained in their allocated treatment group until discharge, with an option to switch to the inpatient setting for a maximum of 3 days if there was a risk of suicide. If a longer stay was required, the participant would be considered a drop-out from the AT_HOME program; however, this did not occur, and such scenarios are also common in usual practice. Furthermore, an intention-to-treat principles was applied.	Y / PY / PN / N / NI
4.2. **If Y/PY to 4.1**: Were these deviations from intended intervention unbalanced between groups *and* likely to have affected the outcome?		NA / Y / PY / PN / N / NI
**If your aim for this study is to assess the effect of starting and adhering to intervention, answer questions 4.3 to 4.6**		
4.3. Were important co-interventions balanced across intervention groups?		Y / PY / PN / N / NI
4.4. Was the intervention implemented successfully for most participants?		Y / PY / PN / N / NI
4.5. Did study participants adhere to the assigned intervention regimen?		Y / PY / PN / N / NI
4.6. **If N/PN to 4.3, 4.4 or 4.5**: Was an appropriate analysis used to estimate the effect of starting and adhering to the intervention?		NA / Y / PY / PN / N / NI
**Risk of bias judgement**	**Low risk of bias:** Any deviations from intended intervention reflected usual practice.	Low / Moderate / Serious / Critical / NI
Optional: What is the predicted direction of bias due to deviations from the intended interventions?		Favours experimental / Favours comparator / Towards null /Away from null / Unpredictable

Article title: Efficacy of home treatment and inpatient treatment for children and adolescents in psychiatric crisis: A systematic review and meta-analysis

Journal: European Child & Adolescent Psychiatry

Authors: Karolina Foremnik, Gaby Sroczynski, Jan Stratil, Marjan Arvandi, Anja Neumann, Barbara Buchberger
Medical Faculty, University of Duisburg-Essen, Germany Corresponding author (KF) E-Mail: karolina.foremnik@uni-due.de

**Bias due to missing data**		
5.1 Were outcome data available for all, or nearly all, participants?	**Yes:** Outcome data for the clinician-rated HoNOSCA score were available for nearly all patients, with only one dropout in the AT_HOME group due to an aggressive act against a treatment team member. An intention-to-treat (ITT) approach was implemented from the outset, ensuring that all assigned participants were included in the analysis.<br><br>Data on treatment satisfaction were missing; however, as this was not considered an outcome measure in our systematic review, it does not impact the assessment (response rate between 50% and 81%).	Y / PY / PN / N / NI
5.2 Were participants excluded due to missing data on intervention status?	**No:** The intervention status was clearly defined from the start to the end of the intervention, ensuring that no participants were excluded due to missing data.	Y / PY / PN / N / NI
5.3 Were participants excluded due to missing data on other variables needed for the analysis?	**Probably no:** There is no evidence that participants were excluded due to missing data that should be controlled as confounders in the study.	Y / PY / PN / N / NI
5.4 **If PN/N to 5.1, or Y/PY to 5.2 or 5.3**: Are the proportion of participants and reasons for missing data similar across interventions?	.	NA / Y / PY / PN / N / NI
5.5 **If PN/N to 5.1, or Y/PY to 5.2 or 5.3**: Is there evidence that results were robust to the presence of missing data?	.	NA / Y / PY / PN / N / NI
**Risk of bias judgement**	**Low risk of bias:** Data were reasonably complete. Only one dropout in the AT_HOME group, but intention-to-treat (ITT) approach was implemented from the outset.	Low / Moderate / Serious / Critical / NI
Optional: What is the predicted direction of bias due to missing data?		Favours experimental / Favours comparator / Towards null /Away from null / Unpredictable

Article title: Efficacy of home treatment and inpatient treatment for children and adolescents in psychiatric crisis: A systematic review and meta-analysis

Journal: European Child & Adolescent Psychiatry

Authors: Karolina Foremnik, Gaby Sroczynski, Jan Stratil, Marjan Arvandi, Anja Neumann, Barbara Buchberger
Medical Faculty, University of Duisburg-Essen, Germany Corresponding author (KF) E-Mail: karolina.foremnik@uni-due.de

**Bias in measurement of outcomes**		
6.1 Could the outcome measure have been influenced by knowledge of the intervention received?	**Probably yes:** Positive expectations towards preferred treatment may have influenced both the choice of treatment and its outcomes (this could introduce comparable bias in both groups).<br><br>Hospitalization may be associated with greater disruption of daily life than home treatment. This may explain why self-rated psychopathological distress was higher in the hospital group than in the AT_Home group.	Y / PY / PN / N / NI
6.2 Were outcome assessors aware of the intervention received by study participants?	**Yes:** HoNOSCA and GAF were not rated by independent researchers but by clinical staff who were not blinded to treatment condition. But therapists rated the treatment outcome for their own patients, so the bias should be similar in both groups.	Y / PY / PN / N / NI
6.3 Were the methods of outcome assessment comparable across intervention groups?	**Yes:** The assessment for both intervention groups was conducted at two time points (baseline and postline), using the same standardized and objective measurement methods (HoNOSCA, HoNOSCA-SR and GAF).	Y / PY / PN / N / NI
6.4 Were any systematic errors in measurement of the outcome related to intervention received?	**Probably no:** Clinicians in both groups received periodic training to ensure the reliability of HoNOSCA assessments. Therapists rated treatment outcomes for their own patients, which could introduce comparable bias in both groups, but no systematic bias between both groups. Additionally, using both clinician-rated and self-rated HoNOSCA assessments provides a more comprehensive picture of the psychopathological burden.	Y / PY / PN / N / NI
**Risk of bias judgement**	**Moderate risk of bias:** One outcome measure was subjective (self-rated HoNOSCA-SR) and therefore vulnerable to influence by knowledge of the intervention received by study participants) and the outcome was assessed by assessors aware of the intervention received by study participants.	Low / Moderate / Serious / Critical / NI

Authors: Karolina Foremnik, Gaby Sroczynski, Jan Stratil, Marjan Arvandi, Anja Neumann, Barbara Buchberger
Medical Faculty, University of Duisburg-Essen, Germany Corresponding author (KF) E-Mail: karolina.foremnik@uni-due.de

Optional: What is the predicted direction of bias due to measurement of outcomes?		Favours experimental / Favours comparator / Towards null /Away from null / Unpredictable

Authors: Karolina Foremnik, Gaby Sroczynski, Jan Stratil, Marjan Arvandi, Anja Neumann, Barbara Buchberger
Medical Faculty, University of Duisburg-Essen, Germany Corresponding author (KF) E-Mail: karolina.foremnik@uni-due.de

**Bias in selection of the reported result**		
Is the reported effect estimate likely to be selected, on the basis of the results, from...	**Probably no:** No preregistered protocol or statistical analysis plan is available. However, psychopathological distress as an outcome domain was measured and analyzed for both treatment groups (AT_HOME and I-TAU) using several measures (clinician- and self-rated). However, no information is provided as to why the GAF score (for psychosocial functioning) was measured only at baseline and not at postline.	
7.1. ... multiple outcome *measurements* within the outcome domain?		Y / PY / PN / N / NI
7.2 ... multiple *analyses* of the intervention-outcome relationship?	**Probably No:** No preregistered protocol or statistical analysis plan is available. But there are no indications that different methods of analysis or multiple calculations of results were used and only selectively reported.	Y / PY / PN / N / NI
7.3 ... different *subgroups*?	**No:** The results are based on the entire sample of participants in the two groups (AT_HOME and I-TAU), without subgroups being analyzed separately. There is therefore no risk that the results have only been presented for certain subgroups or that certain groups have been excluded from the results analysis.	Y / PY / PN / N / NI
**Risk of bias judgement**	**Moderate risk of bias:** There is no protocol or analysis plan, but the outcome measurements are clearly defined and consistent and there is no indication of selection of the reported analysis from among multiple analyses and there is no indication of selection of the cohort or subgroups for analysis and reporting on the basis of the results.	Low / Moderate / Serious / Critical / NI
Optional: What is the predicted direction of bias due to selection of the reported result?		Favours experimental / Favours comparator / Towards null /Away from null / Unpredictable

Authors: Karolina Foremnik, Gaby Sroczynski, Jan Stratil, Marjan Arvandi, Anja Neumann, Barbara Buchberger
Medical Faculty, University of Duisburg-Essen, Germany Corresponding author (KF) E-Mail: karolina.foremnik@uni-due.de

**Overall bias**		
**Risk of bias judgement**	**Moderate risk of bias**	Low / Moderate / Serious / Critical / NI
Optional: What is the overall predicted direction of bias for this outcome?		Favours experimental / Favours comparator / Towards null /Away from null / Unpredictable

This work is licensed under a Creative Commons Attribution-NonCommercial-NoDerivatives 4.0 International License

Article title: Efficacy of home treatment and inpatient treatment for children and adolescents in psychiatric crisis: A systematic review and meta-analysis
Journal: European Child & Adolescent Psychiatry
Authors: Karolina Foremnik, Gaby Sroczynski, Jan Stratil, Marjan Arvandi, Anja Neumann, Barbara Buchberger
Medical Faculty, University of Duisburg-Essen, Germany Corresponding author (KF) E-Mail: karolina.foremnik@uni-due.de

Risk of Bias in Non-Randomized Studies (ROBINS-I)

Risk of bias assessment Graf et al., 2025

Signalling questions	Description	Response options
**Bias due to confounding**		
1.1 Is there potential for confounding of the effect of intervention in this study?<br>**If N/PN to 1.1:** the study can be considered to be at low risk of bias due to confounding and no further signalling questions need be considered	**Probably yes:** There is potential for confounding of the effect of intervention, as the group assignment is not random but based on personal preferences (choice-based allocation).<br><br>Therefore, there was an uneven distribution of participants in the intervention groups (AT_HOME: N= 27; I-TAU: N=48). Primary diagnoses differed, with more affective disorders in the I-TAU group and more anxiety disorders in the HT group.	Y / PY / PN / N
**If Y/PY to 1.1**: determine whether there is a need to assess time-varying confounding:		
1.2. Was the analysis based on splitting participants' follow up time according to intervention received?<br>**If N/PN**, answer questions relating to baseline confounding (1.4 to 1.6)<br>**If Y/PY**, go to question 1.3.	**No:** Splitting participants' follow-up time by intervention received was not necessary, as there was no switching between interventions *(while it was considered that patients in the AT_HOME group might require brief hospitalization (up to 3 days) during the treatment period, for instance due to suicidal tendencies, this situation did not occur.)*	NA / Y / PY / PN / N / NI
1.3. Were intervention discontinuations or switches likely to be related to factors that are prognostic for the outcome?<br>**If N/PN**, answer questions relating to baseline confounding (1.4 to 1.6)		NA / Y / PY / PN / N / NI

**If Y/PY**, answer questions relating to both baseline and time-varying confounding (1.7 and 1.8)		

Authors: Karolina Foremnik, Gaby Sroczynski, Jan Stratil, Marjan Arvandi, Anja Neumann, Barbara Buchberger
Medical Faculty, University of Duisburg-Essen, Germany Corresponding author (KF) E-Mail: karolina.foremnik@uni-due.de

**Questions relating to baseline confounding only**		
1.4. Did the authors use an appropriate analysis method that controlled for all the important confounding domains?	**Yes:** Baseline differences due to lack of randomisation and differences in treatment duration were accounted for by inverse probability weighting (IPW).	NA / Y / PY / PN / N / NI
1.5. **If Y/PY to 1.4**: Were confounding domains that were controlled for measured validly and reliably by the variables available in this study?	**Probably no**: Most confounding domains controlled for were validly and reliably measured by the variables available in this study (e.g., sex, age, treatment duration).<br>The only limitation concerns post-discharge service use, which was assessed via self-report and is therefore subject to recall bias.	NA / Y / PY / PN / N / NI
1.6. Did the authors control for any post-intervention variables that could have been affected by the intervention?	**No information**	NA / Y / PY / PN / N / NI
**Questions relating to baseline and time-varying confounding**		
1.7. Did the authors use an appropriate analysis method that controlled for all the important confounding domains and for time-varying confounding?	**Yes:** Baseline differences due to lack of randomisation were accounted for by IPW and mixed models. Time-varying confounding was not relevant to the study design, as treatment assignment was fixed, and follow-up time was not split based on exposure changes.	NA / Y / PY / PN / N / NI
1.8. **If Y/PY to 1.7**: Were confounding domains that were controlled for measured validly and reliably by the variables available in this study?	**Probably no.** See 1.5.	NA / Y / PY / PN / N / NI

Authors: Karolina Foremnik, Gaby Sroczynski, Jan Stratil, Marjan Arvandi, Anja Neumann, Barbara Buchberger
Medical Faculty, University of Duisburg-Essen, Germany Corresponding author (KF) E-Mail: karolina.foremnik@uni-due.de

**Risk of bias judgement**	**Moderate risk of bias:** There is potential for confounding due to the non-randomized, choice-based allocation of participants. While key baseline confounders such as age, sex, treatment duration, and post-discharge service use were addressed using inverse probability weighting (IPW), some important domains—particularly diagnostic differences (e.g., overrepresentation of anxiety disorders in the HT arm)—were not fully accounted for. Additionally, the use of self-reported data to assess post-discharge treatment introduces some risk of measurement bias. Nevertheless, the impact of these limitations on the main outcome estimates is likely limited due to adjustment for major covariates and consistent findings in sensitivity analyses.	Low / Moderate / Serious / Critical / NI
Optional: What is the predicted direction of bias due to confounding?		Favours experimental / Favours comparator / Unpredictable

Authors: Karolina Foremnik, Gaby Sroczynski, Jan Stratil, Marjan Arvandi, Anja Neumann, Barbara Buchberger
Medical Faculty, University of Duisburg-Essen, Germany Corresponding author (KF) E-Mail: karolina.foremnik@uni-due.de

**Bias in selection of participants into the study**		
2.1. Was selection of participants into the study (or into the analysis) based on participant characteristics observed after the start of intervention?<br>**If N/PN to 2.1:** go to 2.4<br><br>2.2. **If Y/PY to 2.1**: Were the post-intervention variables that influenced selection likely to be associated with intervention?<br>2.3 **If Y/PY to 2.2**: Were the post-intervention variables that influenced selection likely to be influenced by the outcome or a cause of the outcome?	**No:** Selection into the analysis was based on participant availability and consent at follow-up, not on characteristics observed after the start of the intervention.	Y / PY / PN / N / NI<br><br>NA / Y / PY / PN / N / NI<br><br>NA / Y / PY / PN / N / NI
2.4. Do start of follow-up and start of intervention coincide for most participants?	**Yes:** The start of follow-up and the start of intervention coincided for most participants in the study, ensuring a clear temporal relationship between treatment exposure and outcome measurement.	Y / PY / PN / N / NI
2.5. **If Y/PY to 2.2 and 2.3, or N/PN to 2.4**: Were adjustment techniques used that are likely to correct for the presence of selection biases?		NA / Y / PY / PN / N / NI
**Risk of bias judgement**	**Low risk of bias:** All participants who would have been eligible for the target trial were included in the study and for each participant, start of follow up and start of intervention coincided.	Low / Moderate / Serious / Critical / NI
Optional: What is the predicted direction of bias due to selection of participants into the study?		Favours experimental / Favours comparator / Towards null /Away from null / Unpredictable

Article title: Efficacy of home treatment and inpatient treatment for children and adolescents in psychiatric crisis: A systematic review and meta-analysis

Journal: European Child & Adolescent Psychiatry

Authors: Karolina Foremnik, Gaby Sroczynski, Jan Stratil, Marjan Arvandi, Anja Neumann, Barbara Buchberger
Medical Faculty, University of Duisburg-Essen, Germany Corresponding author (KF) E-Mail: karolina.foremnik@uni-due.de

**Bias in classification of interventions**		
3.1 Were intervention groups clearly defined?	**Yes:** the interventions for both AT_Home and I-TAU are clearly defined in terms of type, setting, frequency, intensity, and timing of the intervention.	Y / PY / PN / N / NI
3.2 Was the information used to define intervention groups recorded at the start of the intervention?	**Yes:** The information used to define the two groups was recorded at the start of the intervention.	Y / PY / PN / N / NI
3.3 Could classification of intervention status have been affected by knowledge of the outcome or risk of the outcome?	**Probably yes:** The classification of intervention status was likely affected by knowledge of the risk of the outcome, as clinical and parental decisions regarding treatment group assignment could have been based on factors such as symptom severity and family context.	Y / PY / PN / N / NI
**Risk of bias judgement**	**Moderate risk of bias:** Although intervention status was clearly defined and based on pre-intervention data, the self-selected group assignment may have been influenced by knowledge of the risk of the outcome.	Low / Moderate / Serious / Critical / NI
Optional: What is the predicted direction of bias due to classification of interventions?		Favours experimental / Favours comparator / Towards null /Away from null / Unpredictable

Article title: Efficacy of home treatment and inpatient treatment for children and adolescents in psychiatric crisis: A systematic review and meta-analysis
Journal: European Child & Adolescent Psychiatry
Authors: Karolina Foremnik, Gaby Sroczynski, Jan Stratil, Marjan Arvandi, Anja Neumann, Barbara Buchberger
Medical Faculty, University of Duisburg-Essen, Germany Corresponding author (KF) E-Mail: karolina.foremnik@uni-due.de

**Bias due to deviations from intended interventions**		
**If your aim for this study is to assess the effect of assignment to intervention, answer questions 4.1 and 4.2**		
4.1. Were there deviations from the intended intervention beyond what would be expected in usual practice?	**No:** ´The participants remained in their allocated treatment group until discharge, with an option to switch to the inpatient setting for a maximum of 3 days if there was a risk of suicide. If a longer stay was required, the participant would be considered a drop-out from the AT_HOME program; however, this did not occur, and such scenarios are also common in usual practice. Furthermore, an intention-to-treat principles was applied.	Y / PY / PN / N / NI
4.2. **If Y/PY to 4.1**: Were these deviations from intended intervention unbalanced between groups *and* likely to have affected the outcome?		NA / Y / PY / PN / N / NI
**If your aim for this study is to assess the effect of starting and adhering to intervention, answer questions 4.3 to 4.6**		
4.3. Were important co-interventions balanced across intervention groups?		Y / PY / PN / N / NI
4.4. Was the intervention implemented successfully for most participants?		Y / PY / PN / N / NI
4.5. Did study participants adhere to the assigned intervention regimen?		Y / PY / PN / N / NI
4.6. **If N/PN to 4.3, 4.4 or 4.5**: Was an appropriate analysis used to estimate the effect of starting and adhering to the intervention?		NA / Y / PY / PN / N / NI
**Risk of bias judgement**	**Low risk of bias:** Any deviations from intended intervention reflected usual practice.	Low / Moderate / Serious / Critical / NI
Optional: What is the predicted direction of bias due to deviations from the intended interventions?		Favours experimental / Favours comparator / Towards null /Away from null / Unpredictable

Article title: Efficacy of home treatment and inpatient treatment for children and adolescents in psychiatric crisis: A systematic review and meta-analysis
Journal: European Child & Adolescent Psychiatry
Authors: Karolina Foremnik, Gaby Sroczynski, Jan Stratil, Marjan Arvandi, Anja Neumann, Barbara Buchberger
Medical Faculty, University of Duisburg-Essen, Germany Corresponding author (KF) E-Mail: karolina.foremnik@uni-due.de

**Bias due to missing data**		
5.1 Were outcome data available for all, or nearly all, participants?	**No:** 80% of eligible HT patients consented to participate in the follow up, only 53% of eligible I-TAU patients did so, potentially introducing non-response bias.	Y / PY / PN / N / NI
5.2 Were participants excluded due to missing data on intervention status?	**No:** The intervention status was clearly defined from the start to the end of the intervention, ensuring that no participants were excluded due to missing data.	Y / PY / PN / N / NI
5.3 Were participants excluded due to missing data on other variables needed for the analysis?	**Probably no:** There is no evidence that participants were excluded due to missing data that should be controlled as confounders in the study.	Y / PY / PN / N / NI
5.4 **If PN/N to 5.1, or Y/PY to 5.2 or 5.3**: Are the proportion of participants and reasons for missing data similar across interventions?	**No:** See. 5.1.	NA / Y / PY / PN / N / NI
5.5 **If PN/N to 5.1, or Y/PY to 5.2 or 5.3**: Is there evidence that results were robust to the presence of missing data?	**Probably yes:** But handling of missing data was limited (e.g., only simple imputation for HoNOSCA, complete-case analysis for GAF)	NA / Y / PY / PN / N / NI
**Risk of bias judgement**	**Moderate risk of bias:** Proportions of and reasons for missing participants differ slightly across intervention groups and the analysis is unlikely to have removed the risk of bias arising from the missing data.	Low / Moderate / Serious / Critical / NI
Optional: What is the predicted direction of bias due to missing data?		Favours experimental / Favours comparator / Towards null /Away from null / Unpredictable

Article title: Efficacy of home treatment and inpatient treatment for children and adolescents in psychiatric crisis: A systematic review and meta-analysis
Journal: European Child & Adolescent Psychiatry
Authors: Karolina Foremnik, Gaby Sroczynski, Jan Stratil, Marjan Arvandi, Anja Neumann, Barbara Buchberger
Medical Faculty, University of Duisburg-Essen, Germany Corresponding author (KF) E-Mail: karolina.foremnik@uni-due.de

**Bias in measurement of outcomes**		
6.1 Could the outcome measure have been influenced by knowledge of the intervention received?	**Probably yes**: Positive expectations towards preferred treatment may have influenced both the choice of treatment and its outcomes (main outcome ratings were not blinded). One outcome measure was subjective (self-rated HoNOSCA-SR) and therefore vulnerable to influence by knowledge of the intervention received by study participants.	Y / PY / PN / N / NI
6.2 Were outcome assessors aware of the intervention received by study participants?	**Yes:** Outcome assessors were aware of the intervention received at discharge, and possibly at follow-up, though this is not clearly reported. Follow-up assessments were recorded and rerated by a second, blinded rater.	Y / PY / PN / N / NI
6.3 Were the methods of outcome assessment comparable across intervention groups?	**Yes:** The assessment for both intervention groups was conducted at two time points (baseline, postline and follow up), using the same standardized and objective measurement methods (HoNOSCA, HoNOSCA-SR and GAF).	Y / PY / PN / N / NI
6.4 Were any systematic errors in measurement of the outcome related to intervention received?	**Probably no:** Clinicians in both groups received periodic training to ensure the reliability assessments. Additionally, using both clinician-rated and self-rated HoNOSCA assessments provides a more comprehensive picture of the psychopathological burden.	Y / PY / PN / N / NI
**Risk of bias judgement**	**Moderate risk of bias:** One of the outcome measures (self-rated HoNOSCA-SR) was subjective and therefore potentially vulnerable to influence by participants' knowledge of the intervention received. Additionally, outcomes were primarily assessed by raters who were aware of participants' group allocation. However, follow-up assessments were recorded and rerated by a second, blinded rater.	Low / Moderate / Serious / Critical / NI
Optional: What is the predicted direction of bias due to measurement of outcomes?		Favours experimental / Favours comparator / Towards null /Away from null / Unpredictable

Article title: Efficacy of home treatment and inpatient treatment for children and adolescents in psychiatric crisis: A systematic review and meta-analysis

Journal: European Child & Adolescent Psychiatry

Authors: Karolina Foremnik, Gaby Sroczynski, Jan Stratil, Marjan Arvandi, Anja Neumann, Barbara Buchberger
Medical Faculty, University of Duisburg-Essen, Germany Corresponding author (KF) E-Mail: karolina.foremnik@uni-due.de

**Bias in selection of the reported result**		
Is the reported effect estimate likely to be selected, on the basis of the results, from...	**Probably no:** No preregistered protocol or statistical analysis plan is available. There is no evidence of selective reporting from multiple outcome measurements within the same outcome domain. The effect estimates appear to reflect predefined, consistently applied, and comprehensively reported outcome measures.	
7.1. ... multiple outcome *measurements* within the outcome domain?		Y / PY / PN / N / NI
7.2 ... multiple *analyses* of the intervention-outcome relationship?	**Probably No:** No preregistered protocol or statistical analysis plan is available. But there are no indications that different methods of analysis or multiple calculations of results were used and only selectively reported.	Y / PY / PN / N / NI
7.3 ... different *subgroups*?	**No:** The results are based on the entire sample of participants in the two groups (AT_HOME and I-TAU), without subgroups being analyzed separately. There is therefore no risk that the results have only been presented for certain subgroups or that certain groups have been excluded from the results analysis.	Y / PY / PN / N / NI
**Risk of bias judgement**	**Moderate risk of bias:** There is no protocol or analysis plan, but the outcome measurements are clearly defined and consistent and there is no indication of selection of the reported analysis from among multiple analyses and there is no indication of selection of the cohort or subgroups for analysis and reporting on the basis of the results.	Low / Moderate / Serious / Critical / NI
Optional: What is the predicted direction of bias due to selection of the reported result?		Favours experimental / Favours comparator / Towards null /Away from null / Unpredictable

Article title: Efficacy of home treatment and inpatient treatment for children and adolescents in psychiatric crisis: A systematic review and meta-analysis
Journal: European Child & Adolescent Psychiatry
Authors: Karolina Foremnik, Gaby Sroczynski, Jan Stratil, Marjan Arvandi, Anja Neumann, Barbara Buchberger
Medical Faculty, University of Duisburg-Essen, Germany Corresponding author (KF) E-Mail: karolina.foremnik@uni-due.de

Overall bias		
**Risk of bias judgement**	**Moderate risk of bias**	Low / Moderate / Serious / Critical / NI
Optional: What is the overall predicted direction of bias for this outcome?		Favours experimental / Favours comparator / Towards null /Away from null / Unpredictable

This work is licensed under a Creative Commons Attribution-NonCommercial-NoDerivatives 4.0 International License

Article title: Efficacy of home treatment and inpatient treatment for children and adolescents in psychiatric crisis: A systematic review and meta-analysis
Journal: European Child & Adolescent Psychiatry
Authors: Karolina Foremnik, Gaby Sroczynski, Jan Stratil, Marjan Arvandi, Anja Neumann, Barbara Buchberger
Medical Faculty, University of Duisburg-Essen, Germany Corresponding author (KF) E-Mail: karolina.foremnik@uni-due.de

Risk of Bias in Non-Randomized Studies (ROBINS-I)

Risk of bias assessment Schmidt et al., 2006

Signalling questions	Description	Response options
**Bias due to confounding**		
1.1 Is there potential for confounding of the effect of intervention in this study?<br>**If N/PN to 1.1:** the study can be considered to be at low risk of bias due to confounding and no further signalling questions need be considered	**Probably yes:** No randomized group assignment (treatment setting was self-selected). The inpatient group showed significantly higher symptom severity (MEI total score), more lower-class families, and fewer prior inpatient stays compared to the home treatment group.	Y / PY / PN / N
**If Y/PY to 1.1**: determine whether there is a need to assess time-varying confounding:		
1.2. Was the analysis based on splitting participants' follow up time according to intervention received?<br>**If N/PN**, answer questions relating to baseline confounding (1.4 to 1.6)<br>**If Y/PY**, go to question 1.3.	**No:** Splitting participants' follow-up time by intervention received was not necessary, as there was no switching between interventions. Each participant was followed in a single, fixed treatment group.	NA / Y / PY / PN / N / NI
1.3. Were intervention discontinuations or switches likely to be related to factors that are prognostic for the outcome?<br>**If N/PN**, answer questions relating to baseline confounding (1.4 to 1.6)<br>**If Y/PY**, answer questions relating to both baseline and time-varying confounding (1.7 and 1.8)		NA / Y / PY / PN / N / NI

Article title: Efficacy of home treatment and inpatient treatment for children and adolescents in psychiatric crisis: A systematic review and meta-analysis
Journal: European Child & Adolescent Psychiatry
Authors: Karolina Foremnik, Gaby Sroczynski, Jan Stratil, Marjan Arvandi, Anja Neumann, Barbara Buchberger
Medical Faculty, University of Duisburg-Essen, Germany Corresponding author (KF) E-Mail: karolina.foremnik@uni-due.de

**Questions relating to baseline confounding only**		
1.4. Did the authors use an appropriate analysis method that controlled for all the important confounding domains?	**Probably no:** The authors reported relevant baseline differences between groups, but did not apply statistical methods to adjust for these confounders.	NA / Y / PY / PN / N / NI
1.5. **If Y/PY to 1.4**: Were confounding domains that were controlled for measured validly and reliably by the variables available in this study?		NA / Y / PY / PN / N / NI
1.6. Did the authors control for any post-intervention variables that could have been affected by the intervention?	**No:** The authors did not control for any post-intervention variables that could have been influenced by the intervention.	NA / Y / PY / PN / N / NI
**Questions relating to baseline and time-varying confounding**		
1.7. Did the authors use an appropriate analysis method that controlled for all the important confounding domains and for time-varying confounding?	**Probably no:** The authors did not use an analysis method that adequately controlled for all important confounding domains, and time-varying confounding was not relevant in this study.	NA / Y / PY / PN / N / NI
1.8. **If Y/PY to 1.7**: Were confounding domains that were controlled for measured validly and reliably by the variables available in this study?		NA / Y / PY / PN / N / NI
**Risk of bias judgement**	**Serious risk of bias: At least one known important domain was not appropriately measured, or not controlled for:** The study used a non-randomized design with clear baseline differences between groups (e.g., symptom severity), and did not statistically adjust for these.	Low / Moderate / Serious / Critical / NI
Optional: What is the predicted direction of bias due to confounding?		Favours experimental / Favours comparator / Unpredictable

Article title: Efficacy of home treatment and inpatient treatment for children and adolescents in psychiatric crisis: A systematic review and meta-analysis
Journal: European Child & Adolescent Psychiatry
Authors: Karolina Foremnik, Gaby Sroczynski, Jan Stratil, Marjan Arvandi, Anja Neumann, Barbara Buchberger
Medical Faculty, University of Duisburg-Essen, Germany Corresponding author (KF) E-Mail: karolina.foremnik@uni-due.de

**Bias in selection of participants into the study**		
2.1. Was selection of participants into the study (or into the analysis) based on participant characteristics observed after the start of intervention?<br>**If N/PN to 2.1:** go to 2.4	**No:** Group assignment occurred at treatment start, and participants remained in their assigned group until the end of treatment. There is no indication that anyone was included or excluded after the start of the intervention based on post-intervention characteristics (e.g., treatment response).	Y / PY / PN / N / NI
2.2. **If Y/PY to 2.1**: Were the post-intervention variables that influenced selection likely to be associated with intervention?<br>2.3 **If Y/PY to 2.2**: Were the post-intervention variables that influenced selection likely to be influenced by the outcome or a cause of the outcome?		NA / Y / PY / PN / N / NI<br>NA / Y / PY / PN / N / NI
2.4. Do start of follow-up and start of intervention coincide for most participants?	**Yes:** The start of follow-up and the start of intervention coincided for most participants in the study, ensuring a clear temporal relationship between treatment exposure and outcome measurement.	Y / PY / PN / N / NI
2.5. **If Y/PY to 2.2 and 2.3, or N/PN to 2.4**: Were adjustment techniques used that are likely to correct for the presence of selection biases?		NA / Y / PY / PN / N / NI
**Risk of bias judgement**	**Low risk of bias:** All participants who would have been eligible for the target trial were included in the study and for each participant, start of follow up and start of intervention coincided.	Low / Moderate / Serious / Critical / NI
Optional: What is the predicted direction of bias due to selection of participants into the study?		Favours experimental / Favours comparator / Towards null /Away from null / Unpredictable

Article title: Efficacy of home treatment and inpatient treatment for children and adolescents in psychiatric crisis: A systematic review and meta-analysis
Journal: European Child & Adolescent Psychiatry
Authors: Karolina Foremnik, Gaby Sroczynski, Jan Stratil, Marjan Arvandi, Anja Neumann, Barbara Buchberger
Medical Faculty, University of Duisburg-Essen, Germany Corresponding author (KF) E-Mail: karolina.foremnik@uni-due.de

**Bias in classification of interventions**		
3.1 Were intervention groups clearly defined?	**Yes:** the interventions for both "Home treatment program" and "inpatient treatment" are clearly defined in terms of type, setting, frequency, intensity, and timing of the intervention.	Y / PY / PN / N / NI
3.2 Was the information used to define intervention groups recorded at the start of the intervention?	**Yes:** The information used to define the two groups was recorded at the start of the intervention.	Y / PY / PN / N / NI
3.3 Could classification of intervention status have been affected by knowledge of the outcome or risk of the outcome?	**Probably yes:** The classification of intervention status was likely affected by knowledge of the risk of the outcome, as clinical and parental decisions regarding treatment group assignment could have been based on factors such as symptom severity and family context.	Y / PY / PN / N / NI
**Risk of bias judgement**	**Moderate risk of bias:** Although intervention status was clearly defined and based on pre-intervention data, the self-selected group assignment may have been influenced by knowledge of the risk of the outcome.	Low / Moderate / Serious / Critical / NI
Optional: What is the predicted direction of bias due to classification of interventions?		Favours experimental / Favours comparator / Towards null /Away from null / Unpredictable

Article title: Efficacy of home treatment and inpatient treatment for children and adolescents in psychiatric crisis: A systematic review and meta-analysis

Journal: European Child & Adolescent Psychiatry

Authors: Karolina Foremnik, Gaby Sroczynski, Jan Stratil, Marjan Arvandi, Anja Neumann, Barbara Buchberger
Medical Faculty, University of Duisburg-Essen, Germany Corresponding author (KF) E-Mail: karolina.foremnik@uni-due.de

**Bias due to deviations from intended interventions**		
**If your aim for this study is to assess the effect of assignment to intervention, answer questions 4.1 and 4.2**		
4.1. Were there deviations from the intended intervention beyond what would be expected in usual practice?	**No:** The participants remained in their allocated treatment group until discharge and there were no deviations from the intended interventions.	Y / PY / PN / N / NI
4.2. **If Y/PY to 4.1**: Were these deviations from intended intervention unbalanced between groups *and* likely to have affected the outcome?		NA / Y / PY / PN / N / NI
**If your aim for this study is to assess the effect of starting and adhering to intervention, answer questions 4.3 to 4.6**		
4.3. Were important co-interventions balanced across intervention groups?		Y / PY / PN / N / NI
4.4. Was the intervention implemented successfully for most participants?		Y / PY / PN / N / NI
4.5. Did study participants adhere to the assigned intervention regimen?		Y / PY / PN / N / NI
4.6. **If N/PN to 4.3, 4.4 or 4.5**: Was an appropriate analysis used to estimate the effect of starting and adhering to the intervention?		NA / Y / PY / PN / N / NI
**Risk of bias judgement**	**Low risk of bias:** Any deviations from intended intervention reflected usual practice.	Low / Moderate / Serious / Critical / NI
Optional: What is the predicted direction of bias due to deviations from the intended interventions?		Favours experimental / Favours comparator / Towards null /Away from null / Unpredictable

Article title: Efficacy of home treatment and inpatient treatment for children and adolescents in psychiatric crisis: A systematic review and meta-analysis
Journal: European Child & Adolescent Psychiatry
Authors: Karolina Foremnik, Gaby Sroczynski, Jan Stratil, Marjan Arvandi, Anja Neumann, Barbara Buchberger
Medical Faculty, University of Duisburg-Essen, Germany Corresponding author (KF) E-Mail: karolina.foremnik@uni-due.de

**Bias due to missing data**		
5.1 Were outcome data available for all, or nearly all, participants?	**Probably yes:** Outcome data were available for nearly all participants in both groups. In the home treatment group, 59 of 70 patients who completed the program were reassessed at follow-up (84%), and in the inpatient comparison group, 30 of 35 participants (86%) were followed up at t3. The proportions were high and similar across groups.	Y / PY / PN / N / NI
5.2 Were participants excluded due to missing data on intervention status?	**No:** The intervention status was clearly defined from the start to the end of the intervention, ensuring that no participants were excluded due to missing data.	Y / PY / PN / N / NI
5.3 Were participants excluded due to missing data on other variables needed for the analysis?	**Probably no:** There is no evidence that participants were excluded due to missing data that should be controlled as confounders in the study.	Y / PY / PN / N / NI
5.4 **If PN/N to 5.1, or Y/PY to 5.2 or 5.3**: Are the proportion of participants and reasons for missing data similar across interventions?		NA / Y / PY / PN / N / NI
5.5 **If PN/N to 5.1, or Y/PY to 5.2 or 5.3**: Is there evidence that results were robust to the presence of missing data?		NA / Y / PY / PN / N / NI
**Risk of bias judgement**	**Low risk of bias:** Proportions of and reasons for missing participants were similar across intervention groups.	Low / Moderate / Serious / Critical / NI
Optional: What is the predicted direction of bias due to missing data?		Favours experimental / Favours comparator / Towards null /Away from null / Unpredictable

Article title: Efficacy of home treatment and inpatient treatment for children and adolescents in psychiatric crisis: A systematic review and meta-analysis
Journal: European Child & Adolescent Psychiatry
Authors: Karolina Foremnik, Gaby Sroczynski, Jan Stratil, Marjan Arvandi, Anja Neumann, Barbara Buchberger
Medical Faculty, University of Duisburg-Essen, Germany Corresponding author (KF) E-Mail: karolina.foremnik@uni-due.de

**Bias in measurement of outcomes**		
6.1 Could the outcome measure have been influenced by knowledge of the intervention received?	**Probably yes**: Positive expectations towards preferred treatment may have influenced both the choice of treatment and its outcomes (main outcome ratings were not blinded).	Y / PY / PN / N / NI
6.2 Were outcome assessors aware of the intervention received by study participants?	**Probably yes:** While a blinded evaluation of global treatment effects was included, the main outcome measures (e.g. MEI) were assessed by non-blinded clinicians.	Y / PY / PN / N / NI
6.3 Were the methods of outcome assessment comparable across intervention groups?	**Yes:** The assessment for both intervention groups was conducted at the same time points using the same standardized and objective measurement methods.	Y / PY / PN / N / NI
6.4 Were any systematic errors in measurement of the outcome related to intervention received?	**Probably yes:** Patients in the home treatment group who completed the follow-up had somewhat more favorable baseline characteristics than those who dropped out. This may have produced a slight overestimation of the home treatment effects up to follow up.	Y / PY / PN / N / NI
**Risk of bias judgement**	**Moderate risk of bias:** The methods of outcome assessment were comparable across intervention groups and the outcome measure is probably only minimally influenced by knowledge of the intervention received by study participants and any error in measuring the outcome is probably only minimally related to intervention status.	Low / Moderate / Serious / Critical / NI
Optional: What is the predicted direction of bias due to measurement of outcomes?		Favours experimental / Favours comparator / Towards null /Away from null / Unpredictable

Article title: Efficacy of home treatment and inpatient treatment for children and adolescents in psychiatric crisis: A systematic review and meta-analysis
Journal: European Child & Adolescent Psychiatry
Authors: Karolina Foremnik, Gaby Sroczynski, Jan Stratil, Marjan Arvandi, Anja Neumann, Barbara Buchberger
Medical Faculty, University of Duisburg-Essen, Germany Corresponding author (KF) E-Mail: karolina.foremnik@uni-due.de

**Bias in selection of the reported result**		
Is the reported effect estimate likely to be selected, on the basis of the results, from...	**Probably no:** No preregistered protocol or statistical analysis plan is available. There is no evidence of selective reporting from multiple outcome measurements within the same outcome domain. The effect estimates appear to reflect predefined, consistently applied, and comprehensively reported outcome measures.	
7.1. ... multiple outcome *measurements* within the outcome domain?		Y / PY / PN / N / NI
7.2 ... multiple *analyses* of the intervention-outcome relationship?	**Probably No:** No preregistered protocol or statistical analysis plan is available. But there are no indications that different methods of analysis or multiple calculations of results were used and only selectively reported.	Y / PY / PN / N / NI
7.3 ... different *subgroups*?	**No:** The results are based on the entire sample of participants in the two groups, without subgroups being analyzed separately. There is therefore no risk that the results have only been presented for certain subgroups or that certain groups have been excluded from the results analysis.	Y / PY / PN / N / NI
**Risk of bias judgement**	**Moderate risk of bias:** There is no protocol or analysis plan, but the outcome measurements are clearly defined and consistent and there is no indication of selection of the reported analysis from among multiple analyses and there is no indication of selection of the cohort or subgroups for analysis and reporting on the basis of the results.	Low / Moderate / Serious / Critical / NI
Optional: What is the predicted direction of bias due to selection of the reported result?		Favours experimental / Favours comparator / Towards null /Away from null / Unpredictable

Authors: Karolina Foremnik, Gaby Sroczynski, Jan Stratil, Marjan Arvandi, Anja Neumann, Barbara Buchberger
Medical Faculty, University of Duisburg-Essen, Germany Corresponding author (KF) E-Mail: karolina.foremnik@uni-due.de

**Overall bias**		
**Risk of bias judgement**	**Serious risk of bias:**<br>At least one known important domain was not appropriately measured, or not controlled for: The study used a non-randomized design with clear baseline differences between groups (e.g., symptom severity), and did not statistically adjust for these.<br>.	Low / Moderate / Serious / Critical / NI
Optional: What is the overall predicted direction of bias for this outcome?		Favours experimental / Favours comparator / Towards null /Away from null / Unpredictable

This work is licensed under a Creative Commons Attribution-NonCommercial-NoDerivatives 4.0 International License
