# Supplementary material for: Efficacy of home treatment and inpatient treatment for children and adolescents in psychiatric crisis: a systematic review and meta-analysis
Source: Eur Child Adolesc Psychiatry. 2026 Jun 1;35(7):2103–27. doi: 10.1007/s00787-026-03060-0 (PMC13427882; doi:10.1007/s00787-026-03060-0)
Supplement: Supplementary file 8 — Supplementary Material 8 [file 787_2026_3060_MOESM8_ESM.pdf]

Article title: Efficacy of home treatment and inpatient treatment for children and adolescents in psychiatric crisis: A systematic review and meta-analysis
Journal: European Child & Adolescent Psychiatry
Authors: Karolina Foremnik, Gaby Sroczynski, Jan Stratil, Marjan Arvandi, Anja Neumann, Barbara Buchberger
Medical Faculty, University of Duisburg-Essen, Germany
Corresponding author (KF)
E-Mail: karolina.foremnik@uni-due.de

Detailed presentation of study and intervention characteristics and main individual study findings, as a supplement to Table 1 (Characteristics of included studies)

Tab.1: Overview of Study and Intervention Characteristics

Author/ Year	Study type	Country	No. (n)	Age (years)	Clinical population type	Intervention (intervention type, setting, duration, frequency, team composition, treatment modes)	Comparison intervention (intervention type, duration, team composition, treatment modes)
**Standalone Home Treatment Programs / Multisystemic Therapy (MST) for psychiatric emergencies**							
Henggeler et al., 1999 [35]<br>Schoenwald et al., 2000 [37]<br>Huey et al., 2004 [39]<br>Henggeler et al., 2003 [36]<br>Sheidow et al., 2004 [38]	RCT	USA	113 (IG: 57; CG: 56	10-17 yrs. (M=13)	Youth in psychiatric crisis (e.g., suicidality, psychosis, homicidal ideation, or other risk to self/others)	• Multisystemic Therapy (manualized clinical process and supervisory protocol)<br>• Home- and community-based treatment; 4 months; daily visits initially, later 3×/w; 24/7 crisis support; small caseload (3 families per therapist)<br>• Multidisciplinary team: master's-level clinicians; psychiatrist supervision; crisis caseworkers<br>• Systematic assessment of the multiple determinants of key problems (individual, family, peer, school, and community levels).<br>• Family-centered interventions; systematic assessment; mobilization of family resources; hospitalization if needed, but MST team retains responsibility	• Hospital-based inpatient psychiatric unit<br>• Short term hospitalization (1–2 weeks); followed by outpatient care<br>• Multidisciplinary team (psychiatrist, psychiatry residents, social worker, special education teacher, nursing staff)<br>• Crisis stabilization, psychiatric evaluation, pharmacological treatment behavioral milieu therapy, discharge planning

Article title: Efficacy of home treatment and inpatient treatment for children and adolescents in psychiatric crisis: A systematic review and meta-analysis

Journal: European Child & Adolescent Psychiatry

Authors: Karolina Foremnik, Gaby Sroczynski, Jan Stratil, Marjan Arvandi, Anja Neumann, Barbara Buchberger
Medical Faculty, University of Duisburg-Essen, Germany
Corresponding author (KF)
E-Mail: karolina.foremnik@uni-due.de

**Standalone Home Treatment Programs**							
Graf et al., 2023 [43]	CBA (preference-based group allocation), pilot evaluation	Switzerland	133 (IG: 37; CG: 96)	6- 17 yrs. (M = 13.71 yrs., SD = 2.93)	Adolescents with acute mental disorders (affective, anxiety, behavioral); severe risk cases (e.g., suicidality, child welfare) excluded	• AT_HOME program (inpatient-equivalent form of outreach treatment)<br>• Home-based treatment (including school/ workplace visits); avg. duration 84 days; at least 1×/d (60–120 min); 24/7 crisis phone availability<br>• Team: medical doctors, clinical psychologists, social educators, and specialist nurses<br>• Multimodal specialized therapies (e.g., skills training, resource activation), somatic examinations (e.g., blood sampling, ECG), intensive family involvement/ daily family meetings<br>• Possibility of short-term hospitalization in case of acute suicidality (≤ 3 days), while the AT_HOME team remains responsible for treatment<br>• Treatment focus: symptom reduction and improvement of patient and system functioning	• Inpatient hospitalization (clinic and clinic school)<br>• No temporal limitation (avg. 101 days)<br>• Team: MDs, psychologists, educators, nurses; symptom/ function-focused care<br>• Multimodal specialized therapies (e.g., skills training, resource activation, etc.), individual and group therapy, weekly family meetings.
Graf et al., 2025 [44]			*75* (IG: 27; CG: 48)	6-17 yrs. (M = 15.92 yrs., SD = 2.87)			

Mattejat et al., 2001 [13]	RCT	Germany	Mannheim: 27 (IG: 12; CG: 15/ Marburg: 41(IG: 23; CG: 18)	M=11,75 yrs. (SD= n/a)	Severe psychiatric disorders unresponsive to outpatient care; broad diagnoses incl. conduct, emotional, eating, ADHD, neurotic disorders	Home treatment<br>• Home-based;121 days avg.; 25% clinic incl. group therapy; situational contacts (e.g., grocery shopping); daily to weekly visits based on clinical need<br>• Team: 3 therapists; supervision by senior clinicians & director<br>• Behavioral therapy tailored to diagnosis and family needs	• Inpatient treatment<br>• Avg. 91 days<br>• Standard inpatient care: primarily behaviorally oriented treatment adapted to individual patient needs and treatment intensity.
Schmidt et al., 2006 [5]	CBA (preference-based group allocation)	Switzerland	105 (IG: 70; CG:35)	6-17 yrs. (IG: M= 10.9 yrs., SD= 3.0; CG: M= 11.3 yrs., SD= 3.1)	Children/adolescents with SGKJ ≤ 5 (inpatient-level need); excludes acute suicidality, psychosis, autism, rare disorders (<4% prevalence)	• Home treatment<br>• Home-based; max. 3 months; 2×/w initially, later 1×/w + daily phone support (in crisis)<br>• Team: Psychiatric nurses, medical students; supervised by child psychiatrists<br>• Cognitive-behavioral therapy and parent training; contingency management and social skills training, child-centered and family-focused, tailored to individual difficulties	• Hospital-based child and adolescent psychiatric unit<br>• No predefined limit; mean ≈101 days<br>• Standard inpatient care (therapeutic activities, clinic school attendance)
**Sequential Models (shortened inpatient stay followed by home treatment)**							
Ougrin et al., 2018[17]	RCT	UK	106 (IG: 53;	12-17 yrs. (IG: M= 16.23	Adolescents presenting with psychiatric emergencies; no	• SDS/ ICCS	• Hospital-based inpatient psychiatric treatment, followed

Authors: Karolina Foremnik, Gaby Sroczynski, Jan Stratil, Marjan Arvandi, Anja Neumann, Barbara Buchberger
Medical Faculty, University of Duisburg-Essen, Germany
Corresponding author (KF)
E-Mail: karolina.foremnik@uni-due.de

Ougrin et al., 2021 [40]			CG: 53)	years, SD= 1.54; CG: M= 16.34 years, SD= 1.7)	exclusion based solely on risk level (if risk to self or others was deemed manageable).	• Community-based crisis intervention (mean total 116.3 d) following early discharge from inpatient care (median 34 d)<br>• daily contact possible; available 8–20 h + 24/7 on-call.<br>• Multidisciplinary Team: 1 consultant psychiatrist, 1 administrator, 2–4 CAMHS nurses, 2–4 clinical support workers<br>• Individualized care plans incl. psych. care, school reintegration, intensive case management, flexible community-based treatment	by standard outpatient CAMHS care (with or without day care)<br>• median 50 days<br>• inpatient therapeutic program, psychiatric care, psychosocial interventions, hospital school attendance
Boege et al., 2014 [49]<br>Boege et al., 2015 [50]<br>Boege et al., 2015a [16]<br>Boege et al., 2021 [14]	RCT	Germany	92 (IG: 51; CG: 41)	5-17 yrs.	Psychiatric disorders requiring inpatient care; broad spectrum incl. affective, anxiety, behavioral, eating & psychotic disorders	BeZuHG/ HoT-BITS:<br>• Early inpatient discharge (avg. 48 days) + 12 weeks Hot-BITs hometreatment; 3×/week sessions; 5×/week crisis support available (10 h/day); 24/7 on-call; monthly outpatient follow-up<br>• Team: nurses, psychologist, child/adolescent psychiatrist<br>• Individual & family therapy, psychoeducation, pharmacotherapy; tailored home-based plans	• Hospital-based inpatient psychiatric treatment<br>• Avg. 70 d; outpatient follow-up (1×/month)<br>• Individual therapy, family therapy pharmacotherapy, group therapy, occupational therapy, music therapy, hippotherapy, and/or physical therapy.

Abbreviations: RCT = Randomized Controlled Trial; IG = Intervention Group; CG = Control Group; MST = Multisystemic Therapy; SD = Standard Deviation; MA= Master's Degree; CBA= Controlled Before-After Study; Yrs= years; M= Mean; avg = average; MD= Medical Doctor; SGKJ= Skala zur Gesamtbeurteilung von Kindern und Jugendlichen

Authors: Karolina Foremnik, Gaby Sroczynski, Jan Stratil, Marjan Arvandi, Anja Neumann, Barbara Buchberger
Medical Faculty, University of Duisburg-Essen, Germany
Corresponding author (KF)
E-Mail: karolina.foremnik@uni-due.de

/ Global Assessment Scale for Children and Adolescents; FP Program= Family Preservation Program; 5DR Program= 5-Day Residential Program; BA= Bachelor of Arts; CBT= Cognitive Behavioral Therapy; AAH= Acute At Home; OT= Occupational Therapy; IHT= Intensive Home Treatment; SDS= Supported Discharge Service; ICCS= Intensive Community Care Service; CAMHS= Child and Adolescent Mental Health Services; BeZuHG= Behandelt zu Hause gesund werden; HoT-BITS= Hometreatment brings inpatient-treatment outside

Tab.2: Summary of Outcomes, Risk of Bias and Main Findings Across Included Studies

**Author/ Year**	**Assessment Timepoints**	**Relevant outcome measures**	**Main findings**	**Used in quantitative synthesis***	**Risk of bias**
**Standalone Home Treatment Programs/ Multisystemic Therapy (MST) for psychiatric emergencies**					
Henggeler et al., 1999 [35]	Discharge: MST (~4 months post recruitment)/ Inpatient treatment: (1–2 weeks post recruitment)	• Psychopathology<br>GSI-BSI, CBCL externalizing/ internalizing (parent and teacher rated)<br>• Youth (social) functioning<br>FFS -Self-esteem, CBCL Social, School attendance, arrest.<br>• Family functioning	MST > Hospital. in reducing externalizing symptoms (parent and teacher rated) and improving family functioning (parent-rated family cohesion and youth-rated family adaptability) and school attendance; Hospital > MST in improving self-esteem.	Yes	High (RoB2)

Authors: Karolina Foremnik, Gaby Sroczynski, Jan Stratil, Marjan Arvandi, Anja Neumann, Barbara Buchberger
Medical Faculty, University of Duisburg-Essen, Germany
Corresponding author (KF)
E-Mail: karolina.foremnik@uni-due.de

		FACES-III -family cohesion and adaptability, GSI-BSI (caregiver)			
Schoenwald et al., 2000 [37]	Discharge: MST (~4 months post recruitment)/ Inpatient treatment: (1–2 weeks post recruitment)	• Proportion of youth hospitalized.<br>• Mean days hospitalized.<br>• Mean days per hospitalized youth.<br>Mean length of stay per episode for hospitalized youth	MST significantly reduced hospitalization rates (44% vs. 100%) and total days hospitalized (M = 2.39 vs. 8.82); 57% avoided hospitalization. MST reduced total out-of-home placement days by 57% and placement changes by 36%, no increased use of other placements. No significant group differences in days per episode or per hospitalized youth.	Yes	Some concerns (RoB2)
Huey et al., 2004 [39]	12 months post-treatment	• <u>Family functioning</u>: Parental Control: FFS<br>• <u>Psychopathology:</u> Depressive affect: BSI and CBCL, Hopelessness Scale for Children/ Attempted suicide: CBCL and YRBS/ Suicidal ideation: BSI and YRBS	• MST > Hosp. in reducing youth-reported suicide attempts at 1-year follow-up, but no group differences for youth-rated depressive affect, hopelessness, or suicidal ideation. Caregiver-reported suicide attempt patterns varied by age, gender, and ethnicity.<br>• Temporary effect on caregiver-rated parental control (FFS), returning to baseline at 1 year.	Yes	High (RoB2)
Henggeler et al., 2003 [36]	• Discharge: MST (~4 months post recruitment)/ Inpatient treatment: (1–2 weeks post recruitment)	• Psychopathology: GSI- BSI; CBCL<br>• Youth (social) functioning: FFS (self- esteem); School attendance<br>Family functioning: FACES-III	MST > Hosp. in reducing symptoms and out-of-home placements and improving school attendance and family structure.<br>Group differences dissipated by 12–16 months postrecruitment.	No- Data missing for SMD	High (RoB2)

Authors: Karolina Foremnik, Gaby Sroczynski, Jan Stratil, Marjan Arvandi, Anja Neumann, Barbara Buchberger
Medical Faculty, University of Duisburg-Essen, Germany
Corresponding author (KF)
E-Mail: karolina.foremnik@uni-due.de

	• 6 months post-treatment<br>• 12 months post-treatment				
Sheidow et al., 2004 [38][	• Discharge: MST (~4 months post recruitment)/ Inpatient treatment: (1–2 weeks post recruitment)<br>• 12 months post-treatment	Cost-effectiveness ratio<br>• cost: Medicaid spending ($)<br>• effectiveness: CBCL (externalizing and internalizing score); GSI	MST > Hospital in short-term cost-effectiveness → lower total Medicaid costs (net savings: $1,617/youth) and better outcomes for externalizing, internalizing symptoms, and global distress. MST = Hospital at 12-month follow-up → no significant cost or outcome differences; long-term cost-effectiveness equivalent.	No- only narratively	High (RoB2)
**Standalone Home Treatment Programs**					
Graf et al., 2023 [43]	Discharge	Psychopathology: HoNOSCA; HoNOSCA-SR	Home treatment = Hospital in symptom reduction (HoNOSCA, HoNOSCA-SR); No significant differences between groups after adjustment (AIPW)	No- pilot study; superseded by full trial	Moderate (ROBINS-I)
Graf et al., 2025 [44]	18-24 months post-treatment	• Psychopathology: HoNOSCA; HoNOSCA-SR<br>• Youth (social) functioning: GAF<br>• Readmission rates	Home treatment > Hospital in clinician-rated psychopathology (HoNOSCA) and psychosocial functioning (GAF); No group differences in self-rated psychopathology (HoNOSCA-SR), readmission rates, or service use	Yes	Moderate (ROBINS-I)

Article title: Efficacy of home treatment and inpatient treatment for children and adolescents in psychiatric crisis: A systematic review and meta-analysis
Journal: European Child & Adolescent Psychiatry
Authors: Karolina Foremnik, Gaby Sroczynski, Jan Stratil, Marjan Arvandi, Anja Neumann, Barbara Buchberger
Medical Faculty, University of Duisburg-Essen, Germany
Corresponding author (KF)
E-Mail: karolina.foremnik@uni-due.de

Mattejat et al., 2001 [13]	Discharge: M = 3.7 years post-treatment (range: 2.1 – 5.2 years)	• Psychopathology: Number of marked symptoms (MSS)<br>• Youth (social) functioning: Rating of psychosocial competency: adaption at school or work	Home treatment = Hospital; No significant group differences: both groups improved significantly; effects stable at 3.7-year follow up.	Yes	Some concerns (RoB2)
Schmidt et al., 2006 [5]	• Discharge<br>• 1-year post-treatment	• Psychopathology: MEI/ blind ratings of symptom improvement (−2 to +4)<br>• Functioning: SGKJ; 5D-Scale (Marcus et al., 1993: family, school performance, peers, interests, autonomy)	Post-treatment: inpatient slightly more effective (MEI, SGKJ, school performance); follow-up: no group differences – both groups equally effective (MEI, SGKJ, 5D-Scale); HT showed greater stability, with continued improvement post-treatment, while inpatient effects remained stable.	Yes	Serious (ROBINS-I)
**Sequential Models (shortened inpatient stay followed by home treatment)**					
Ougrin et al., 2018 [17]	6 months after randomization	• Psychopathology: SDQ; Self-harm questionnaire (multiple ≥5 episodes of self-harm)<br>• Youth (social) functioning: CGAS; School attendance/ reintegration; Number of days not in education, employment, or training<br>• Cost-effectiveness ratio<br>• Mean total inpatient days	• SDS > Hospital in reducing inpatient bed-days (–34.1 days; *p*= .01), increasing school reintegration (81% vs. 51%; OR = 4.14), and reducing repeated self-harm episodes (24% vs. 42%; OR = 0.18); significantly fewer days not in employment, education, or training (median 49 vs. 96 days; p=0.04).<br>• No significant differences in symptom reduction (SDQ), functioning (CGAS), or patient satisfaction.<br>• SDS showed higher probability of cost-effectiveness vs. usual care (60% at £20,000–30,000 per QALY; ≥58% based on CGAS, rising to 90% at £5,000).<br>ICER of £183,750/QALY exceeds NICE	Yes	Some concerns (RoB2)

			threshold, suggesting SDS is more cost-saving than more effective		
Ougrin et al., 2021 [40]		• <u>Psychopathology:</u> SHQ; CGI; HoNOSCA<br>• <u>Youth (social) functioning</u>: CIS<br>• Presentations to emergency departments with self-harm<br>• Total presentations to emergency departments<br>• Readmissions to inpatient psychiatric units<br>• Occupied bed-days whilst readmitted<br>• Hospital use in patients admitted to private versus NHS inpatient units	• ICCS > Hospital: significantly fewer multiple self-harm episodes (≥5; OR = 0.18, 95% CI: 0.05–0.64);<br>patients in private inpatient care had on average 118.4 fewer inpatient days in the ICCS group (95% CI: 28.2–208.6).<br>• Other assessed outcomes (e.g., any self-harm, CGI, HoNOSCA, CIS) showed no significant group differences.	Yes	Some concerns (RoB2)
Boege et al., 2014 [15]	Discharge	• <u>Psychopathology</u>: HoNOSCA: CIS; SDQ<br>• <u>Youth (social) functioning</u>: CGAS<br>• Length of inpatient stay.	• No significant group differences in outcomes: CIS, SDQ, HoNOSCA and CGAS)<br>• BeZuHG group showed significantly shorter inpatient stay (47.65 vs. 69.41 days; p = .001)	Yes	Some concerns (RoB2)
Boege et al., 2015 [50]	• Discharge<br>• 8,4 months post-treatment	• <u>Psychopathology</u>: HoNOSCA; CIS; SDQ<br>• <u>Youth (social) functioning</u>: CGAS	No group-level differences reported; within the home treatment group, significantly greater improvements in boys (CIS p = .009, SDQ p < .001), adolescents (SDQ p = .017), and youth with externalizing disorders (SDQ p = .005).	Yes	Some concerns (RoB2)
Boege et al., 2015a [16]	• Discharge (T2)	• <u>Youth (social) functioning:</u> CGAS<br>• Cost-effectiveness ratio	Hot-BITs > TAU: no significant between-group difference in CGAS scores at T2/T3; however, Hot-	No- only narratively	Some concerns (RoB2)

Authors: Karolina Foremnik, Gaby Sroczynski, Jan Stratil, Marjan Arvandi, Anja Neumann, Barbara Buchberger
Medical Faculty, University of Duisburg-Essen, Germany
Corresponding author (KF)
E-Mail: karolina.foremnik@uni-due.de

	• 8 months post-treatment (T3)		BITs was significantly less costly (-€6,900 at T2; -€8,584 at T3).		
Boege et al., 2021 [14]	• Discharge<br>• 8,4 months post-treatment<br>• 4,3 years post-treatment	• Psychopathology: HoNOSCA A/B<br>• Youth (social) functioning: CGAS	No significant group differences at 4.3 years in CGAS, HoNOSCA A/B; BeZuHG showed higher parental satisfaction and lower need for follow-up care.	Yes	Some concerns (RoB2)

Abbreviations: RCT = Randomized Controlled Trial; IG = Intervention Group; CG = Control Group; MST = Multisystemic Therapy; SD = Standard Deviation; GSI-BSI= Global Severity Index- Brief Symptom Inventory; CBCL= The Child Behavior Checklist; FFS= Family Functioning Scale; FACES-III= Family Adaptability and Cohesion Evaluation Scales; YRBS= Youth Risk Behavior Survey; M= mean; RoB = Risk-of-bias tool for randomized trials ; SMD= Standardized Mean Difference; HoNOSCA= Health of the Nation Outcome Scales for Children and Adolescents; AIPW= Augmented Inverse Probability Weighting; ROBINS-I= Risk Of Bias In Non-randomized Studies – of Interventions; GAF= Global Assessment of Functioning; MSS= Marburg Symptom Scale; MEI= Mannheim Parent Interview; SGKJ= Global Assessment Scale for Children and Adolescents; 5D-Scale= Five-Dimensional Assessment of Social Functioning; SCIS= Standardized Client Information System; SSRS= The Social Skills Rating System; ; FP Program= Family Preservation Program; 5DR Program= 5-Day Residential Program; AAH= Acute At Home; BCFPI= Brief Child and Family Phone Interview; CAFAS= Child and Adolescent Functional Assessment Scale; RT= Residential treatment; IFS= Intensive-family service; SDQ= Strengths and Difficulties Questionnaire; CGAS= Children's Global Assessment Scale; SDS= Supported Discharge Service; OR= Odds Ratio; ICER= Incremental Cost-Effectiveness Ratio; CGI= Clinical Global Impression; QALY= Quality-Adjusted Life Year; NICE= National Institute for Health and Care Excellence; HoNOSCA= Health of the Nation Outcome Scales for Children and Adolescents; CIS= Children's Global Assessment Scale –

Authors: Karolina Foremnik, Gaby Sroczynski, Jan Stratil, Marjan Arvandi, Anja Neumann, Barbara Buchberger
Medical Faculty, University of Duisburg-Essen, Germany
Corresponding author (KF)
E-Mail: karolina.foremnik@uni-due.de

Impairment Scale; NHS= National Health Service; ICCS= Intensive Community Care Service; CI= Confidence Interval; BeZuHG= Behandelt zu Hause gesund werden; HoT-BITS= Hometreatment brings inpatient-treatment outside

*Some eligible studies were included in the review but excluded from the meta-analysis due to missing data, insufficient reporting (e.g., only adjusted results), or overlapping populations covered in later, more comprehensive publications. Details are also provided in Table 2, column: ‘used in quantitative syntheses.
