# Supplementary material for: Efficacy of home treatment and inpatient treatment for children and adolescents in psychiatric crisis: a systematic review and meta-analysis
Source: Eur Child Adolesc Psychiatry. 2026 Jun 1;35(7):2103–27. doi: 10.1007/s00787-026-03060-0 (PMC13427882; doi:10.1007/s00787-026-03060-0)
Supplement: Supplementary file 9 — Supplementary Material 9 [file 787_2026_3060_MOESM9_ESM.pdf]

Article title: Efficacy of home treatment and inpatient treatment for children and adolescents in psychiatric crisis: A systematic review and meta-analysis
Journal: European Child & Adolescent Psychiatry
Authors: Karolina Foremnik, Gaby Sroczynski, Jan Stratil, Marjan Arvandi, Anja Neumann, Barbara Buchberger
Medical Faculty, University of Duisburg-Essen, Germany
Corresponding author (KF)
E-Mail: karolina.foremnik@uni-due.de

Sensitivity analyses: Psychosocial functioning- stand alone

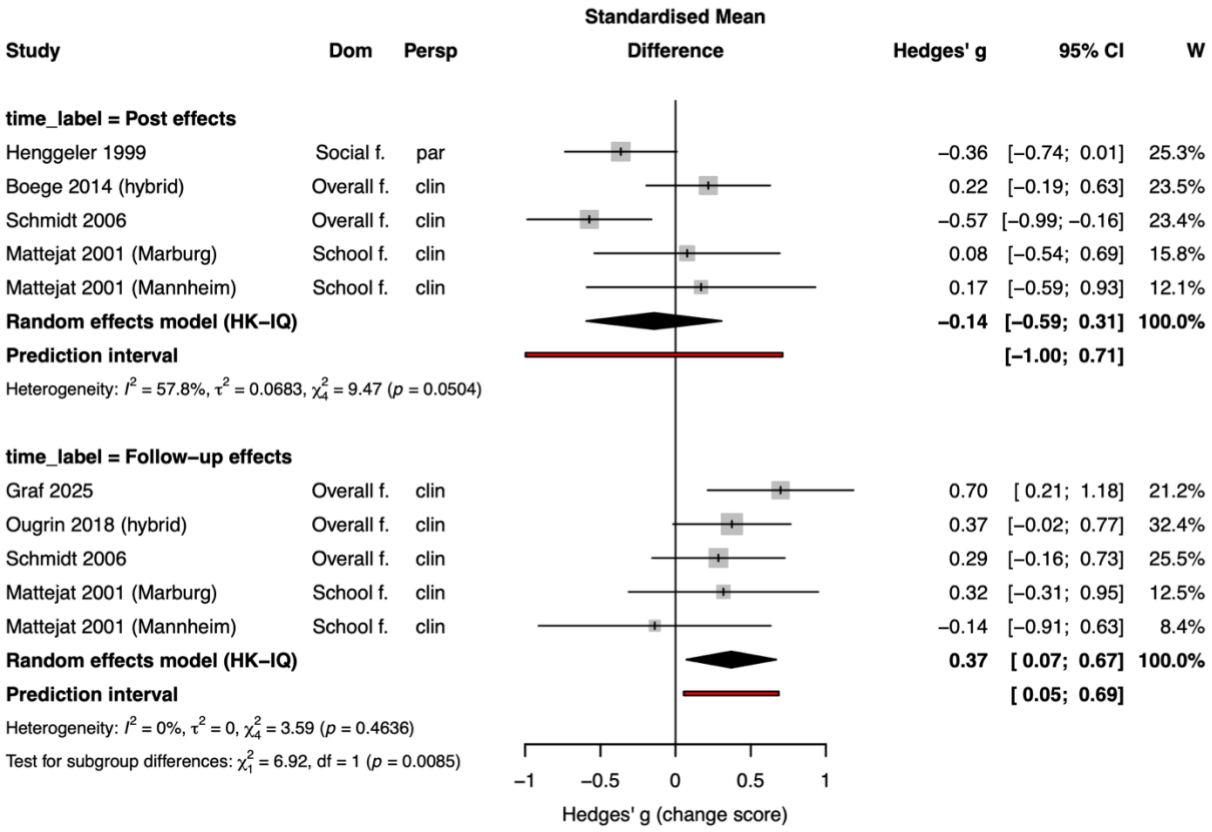

Fig.1: Sensitivity analysis for the outcome Psychosocial functioning (sequential HT models included)

Article title: Efficacy of home treatment and inpatient treatment for children and adolescents in psychiatric crisis: A systematic review and meta-analysis
Journal: European Child & Adolescent Psychiatry
Authors: Karolina Foremnik, Gaby Sroczynski, Jan Stratil, Marjan Arvandi, Anja Neumann, Barbara Buchberger
Medical Faculty, University of Duisburg-Essen, Germany
Corresponding author (KF)
E-Mail: karolina.foremnik@uni-due.de

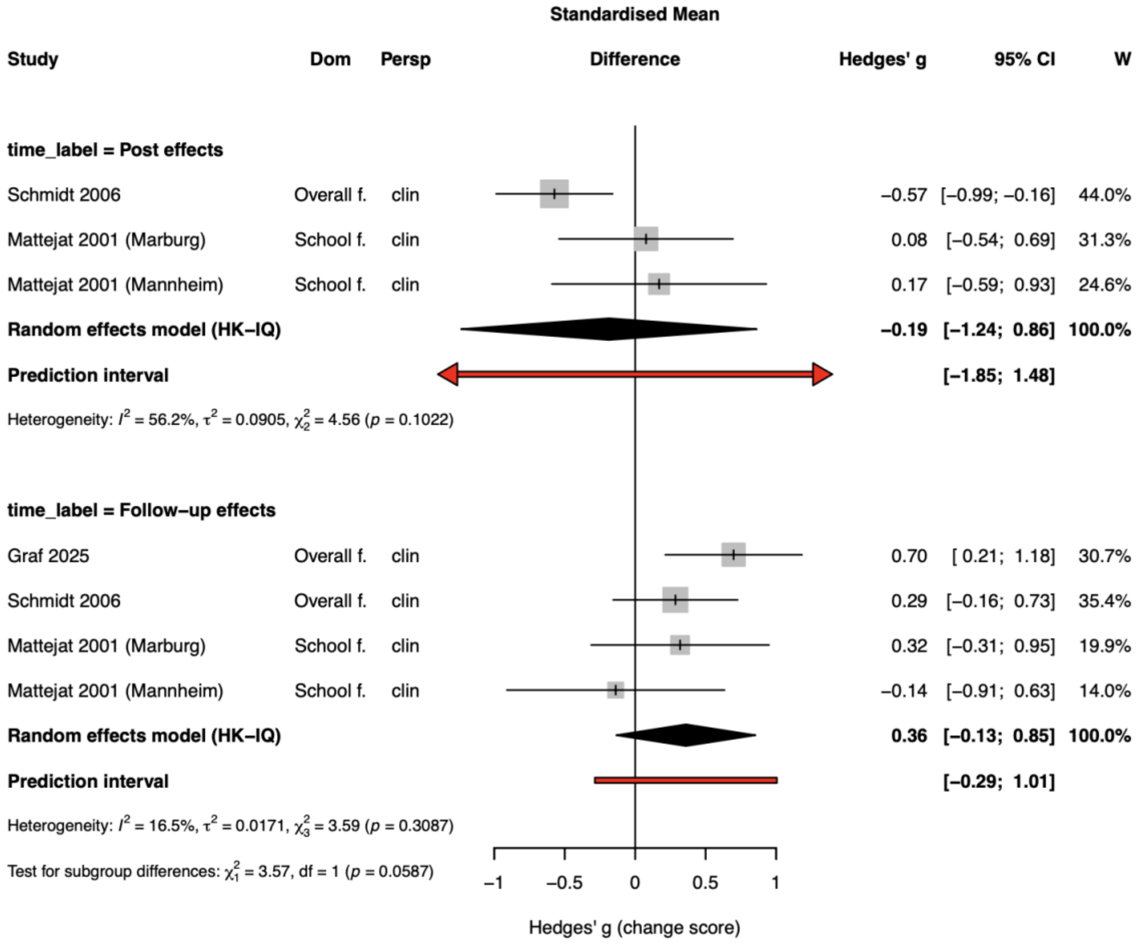

Fig.2: Sensitivity analysis for the outcome Psychosocial functioning (rater- harmonized)

Article title: Efficacy of home treatment and inpatient treatment for children and adolescents in psychiatric crisis: A systematic review and meta-analysis
Journal: European Child & Adolescent Psychiatry
Authors: Karolina Foremnik, Gaby Sroczynski, Jan Stratil, Marjan Arvandi, Anja Neumann, Barbara Buchberger
Medical Faculty, University of Duisburg-Essen, Germany
Corresponding author (KF)
E-Mail: karolina.foremnik@uni-due.de

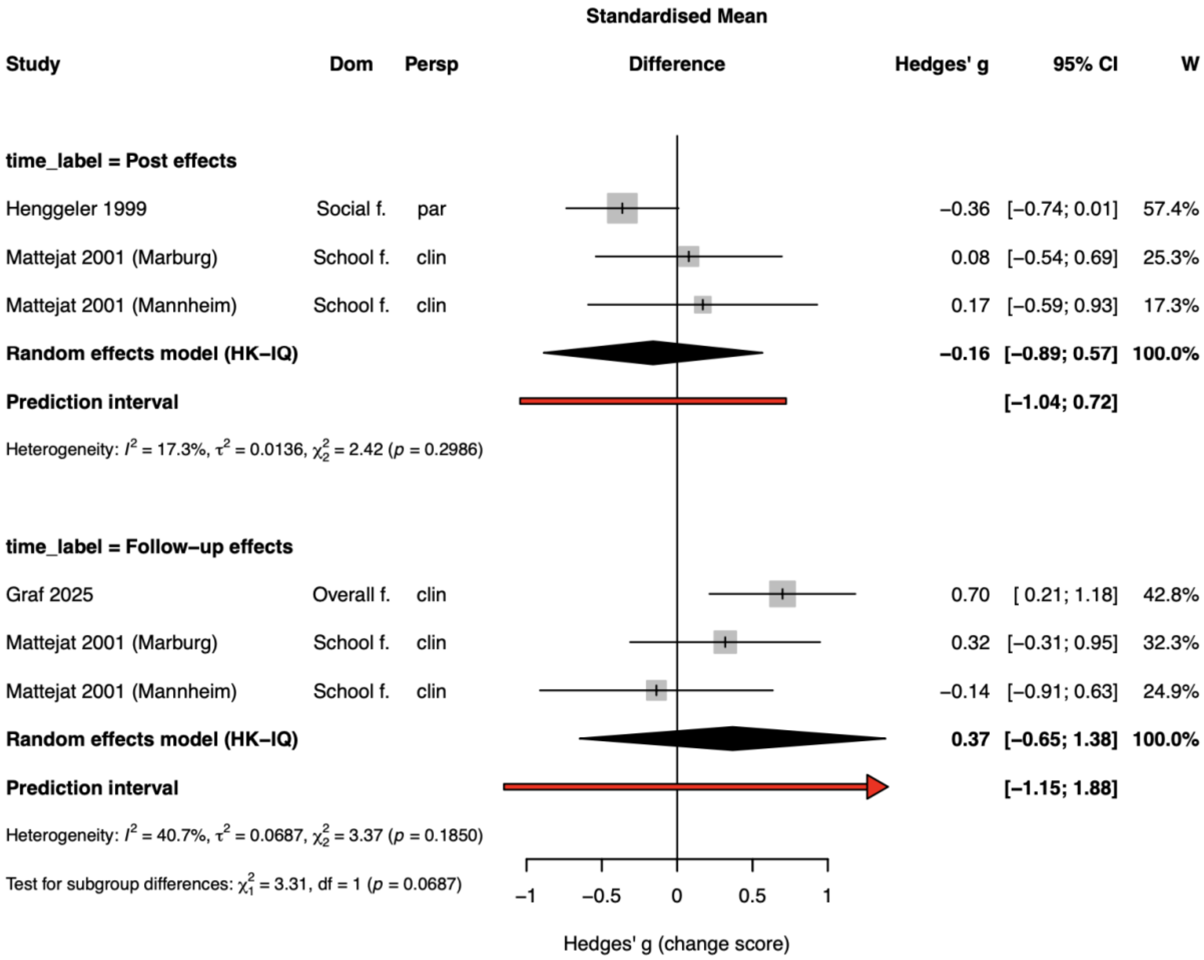

Fig.3: Sensitivity analysis for the outcome Psychosocial functioning (only RCTs)

Article title: Efficacy of home treatment and inpatient treatment for children and adolescents in psychiatric crisis: A systematic review and meta-analysis

Journal: European Child & Adolescent Psychiatry

Authors: Karolina Foremnik, Gaby Sroczynski, Jan Stratil, Marjan Arvandi, Anja Neumann, Barbara Buchberger
Medical Faculty, University of Duisburg-Essen, Germany
Corresponding author (KF)
E-Mail: karolina.foremnik@uni-due.de

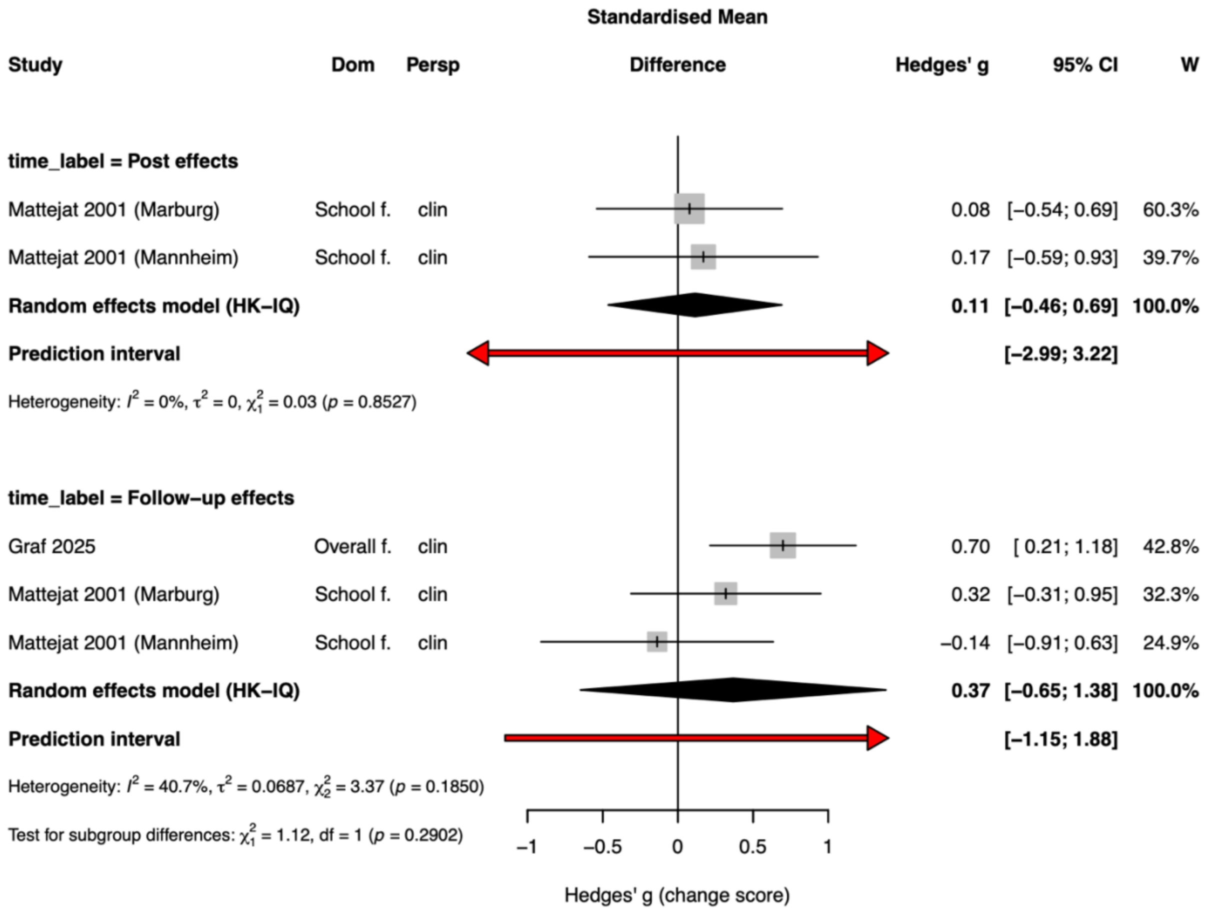

Fig.4: Sensitivity analysis for the outcome Psychosocial functioning (high RoB excluded)

Article title: Efficacy of home treatment and inpatient treatment for children and adolescents in psychiatric crisis: A systematic review and meta-analysis
Journal: European Child & Adolescent Psychiatry
Authors: Karolina Foremnik, Gaby Sroczynski, Jan Stratil, Marjan Arvandi, Anja Neumann, Barbara Buchberger
Medical Faculty, University of Duisburg-Essen, Germany
Corresponding author (KF)
E-Mail: karolina.foremnik@uni-due.de

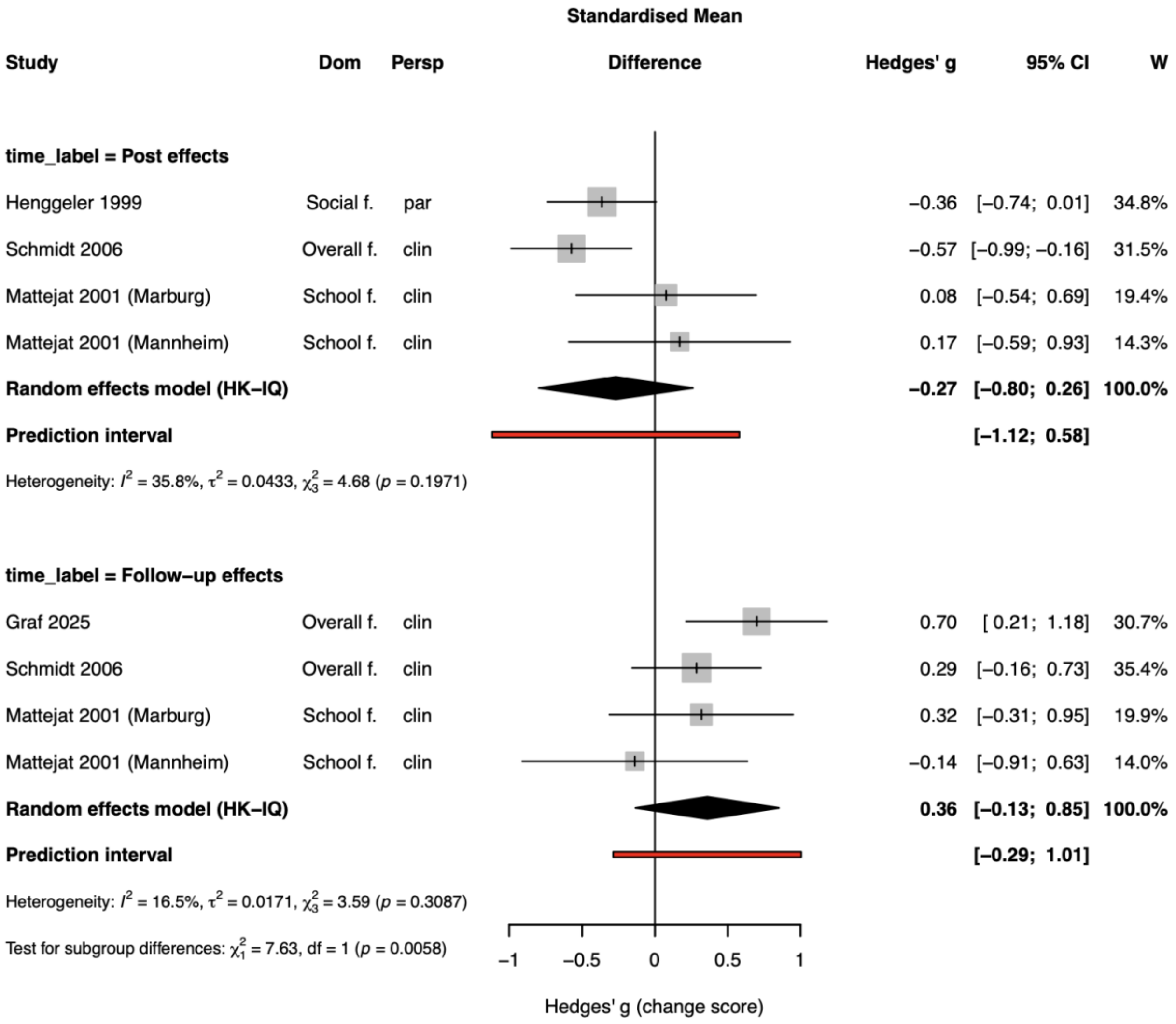

Fig.5: Sensitivity analysis for the outcome Psychosocial functioning (DerSimonian–Laird)

Article title: Efficacy of home treatment and inpatient treatment for children and adolescents in psychiatric crisis: A systematic review and meta-analysis
Journal: European Child & Adolescent Psychiatry
Authors: Karolina Foremnik, Gaby Sroczynski, Jan Stratil, Marjan Arvandi, Anja Neumann, Barbara Buchberger
Medical Faculty, University of Duisburg-Essen, Germany
Corresponding author (KF)
E-Mail: karolina.foremnik@uni-due.de

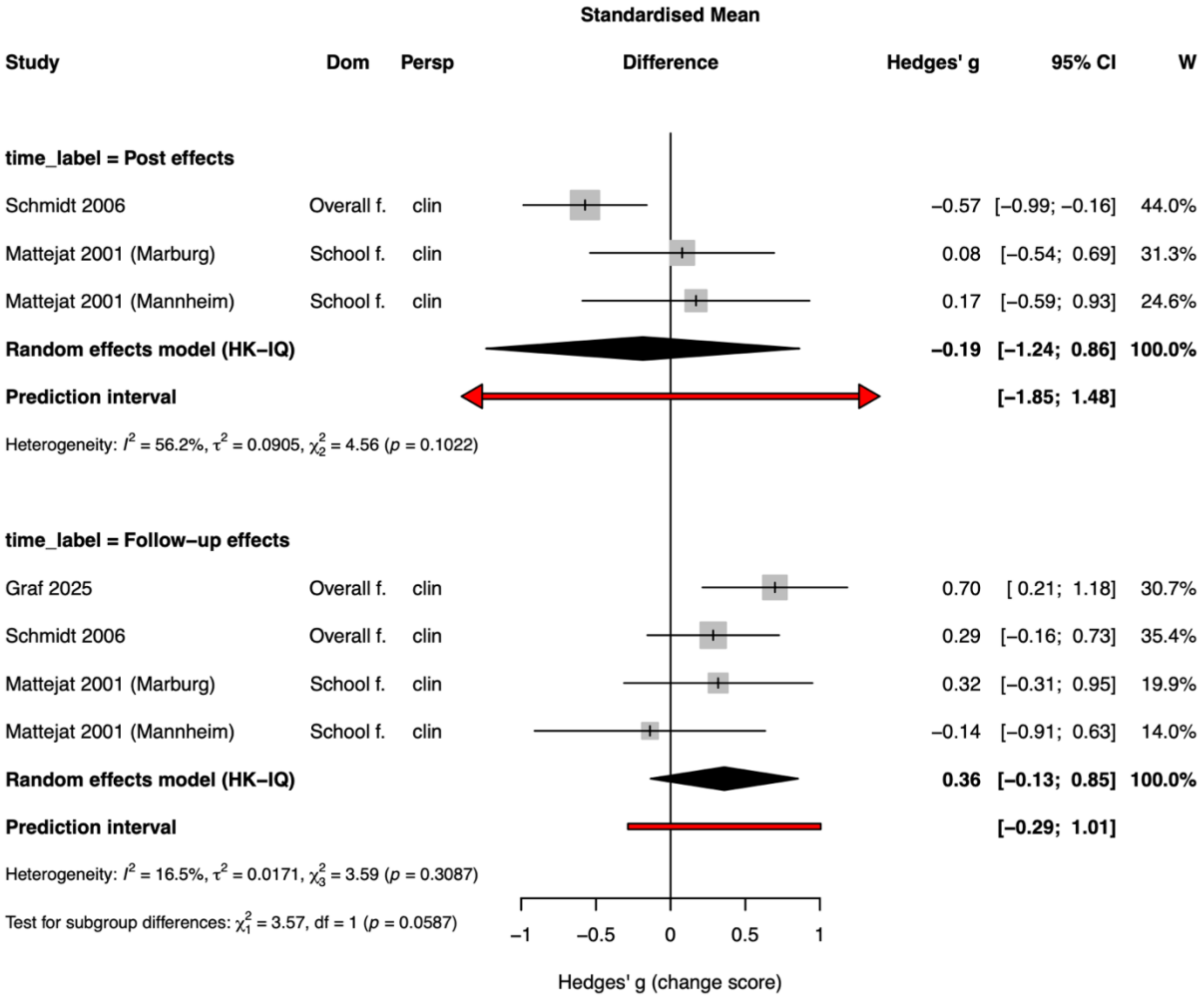

Fig.6: Sensitivity analysis for the outcome Psychosocial functioning (Psychiatric emergencies [under MST treatment] excluded)

Sensitivity analysis for the outcome Psychosocial functioning (domain-harmonized): ***Not conducted due to insufficient data***

Article title: Efficacy of home treatment and inpatient treatment for children and adolescents in psychiatric crisis: A systematic review and meta-analysis
Journal: European Child & Adolescent Psychiatry
Authors: Karolina Foremnik, Gaby Sroczynski, Jan Stratil, Marjan Arvandi, Anja Neumann, Barbara Buchberger
Medical Faculty, University of Duisburg-Essen, Germany
Corresponding author (KF)
E-Mail: karolina.foremnik@uni-due.de

Sensitivity analyses: Psychopathology- stand alone

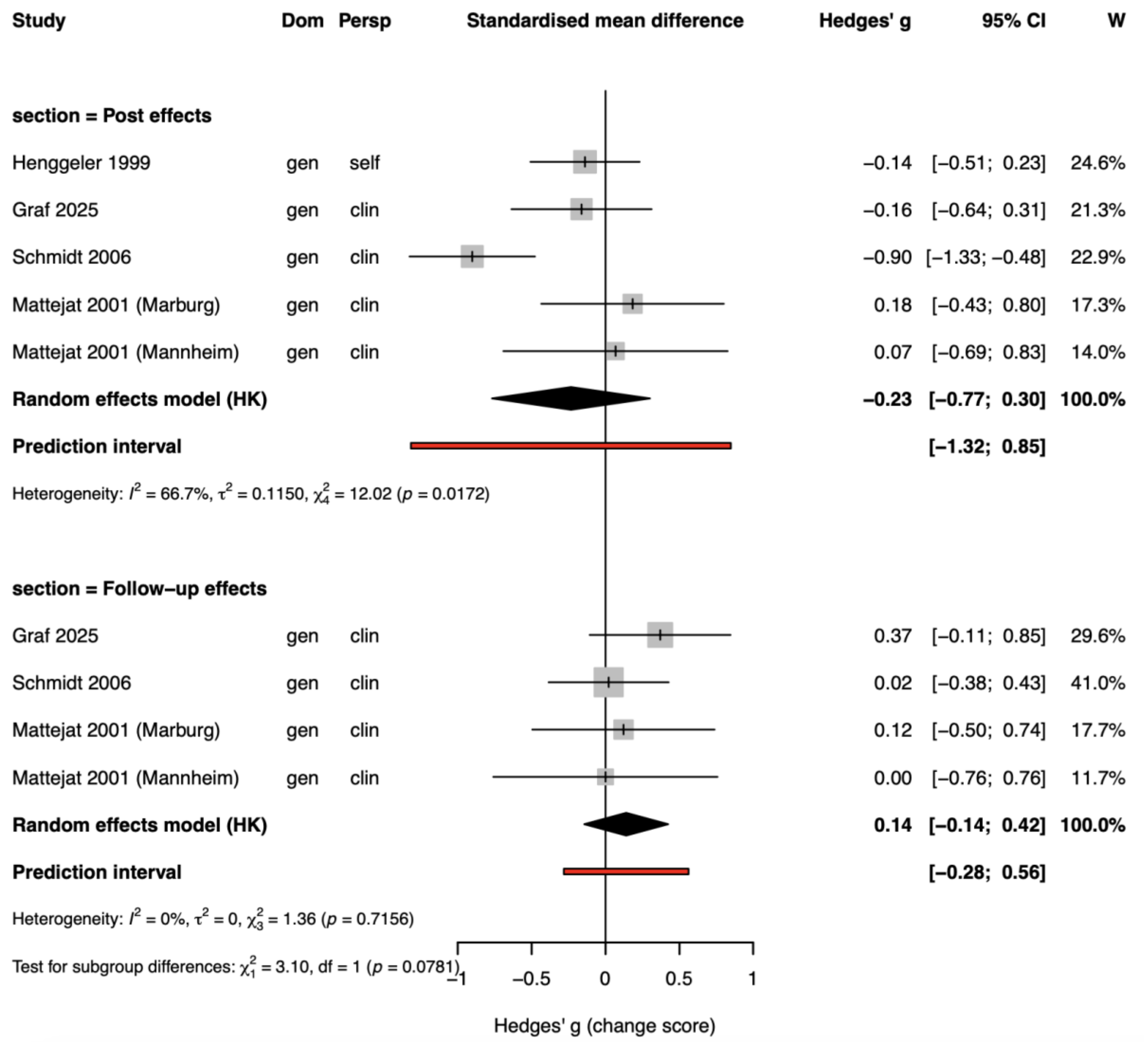

Fig.7: Sensitivity analysis for the outcome Psychopathology (domain-harmonized)

Article title: Efficacy of home treatment and inpatient treatment for children and adolescents in psychiatric crisis: A systematic review and meta-analysis
Journal: European Child & Adolescent Psychiatry
Authors: Karolina Foremnik, Gaby Sroczynski, Jan Stratil, Marjan Arvandi, Anja Neumann, Barbara Buchberger
Medical Faculty, University of Duisburg-Essen, Germany
Corresponding author (KF)
E-Mail: karolina.foremnik@uni-due.de

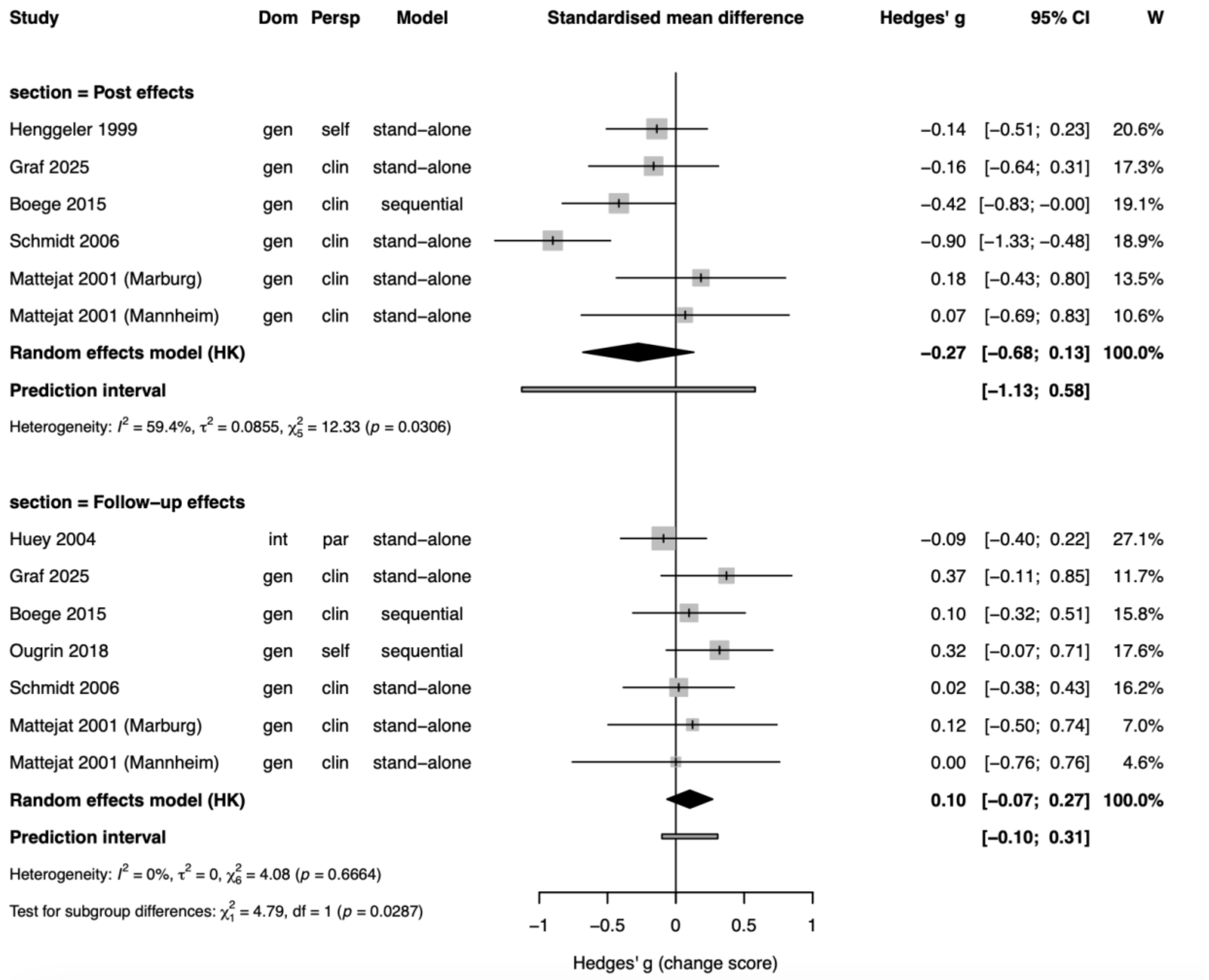

Fig.8: Sensitivity analysis for the outcome Psychopathology (sequential included)

Article title: Efficacy of home treatment and inpatient treatment for children and adolescents in psychiatric crisis: A systematic review and meta-analysis
Journal: European Child & Adolescent Psychiatry
Authors: Karolina Foremnik, Gaby Sroczynski, Jan Stratil, Marjan Arvandi, Anja Neumann, Barbara Buchberger
Medical Faculty, University of Duisburg-Essen, Germany
Corresponding author (KF)
E-Mail: karolina.foremnik@uni-due.de

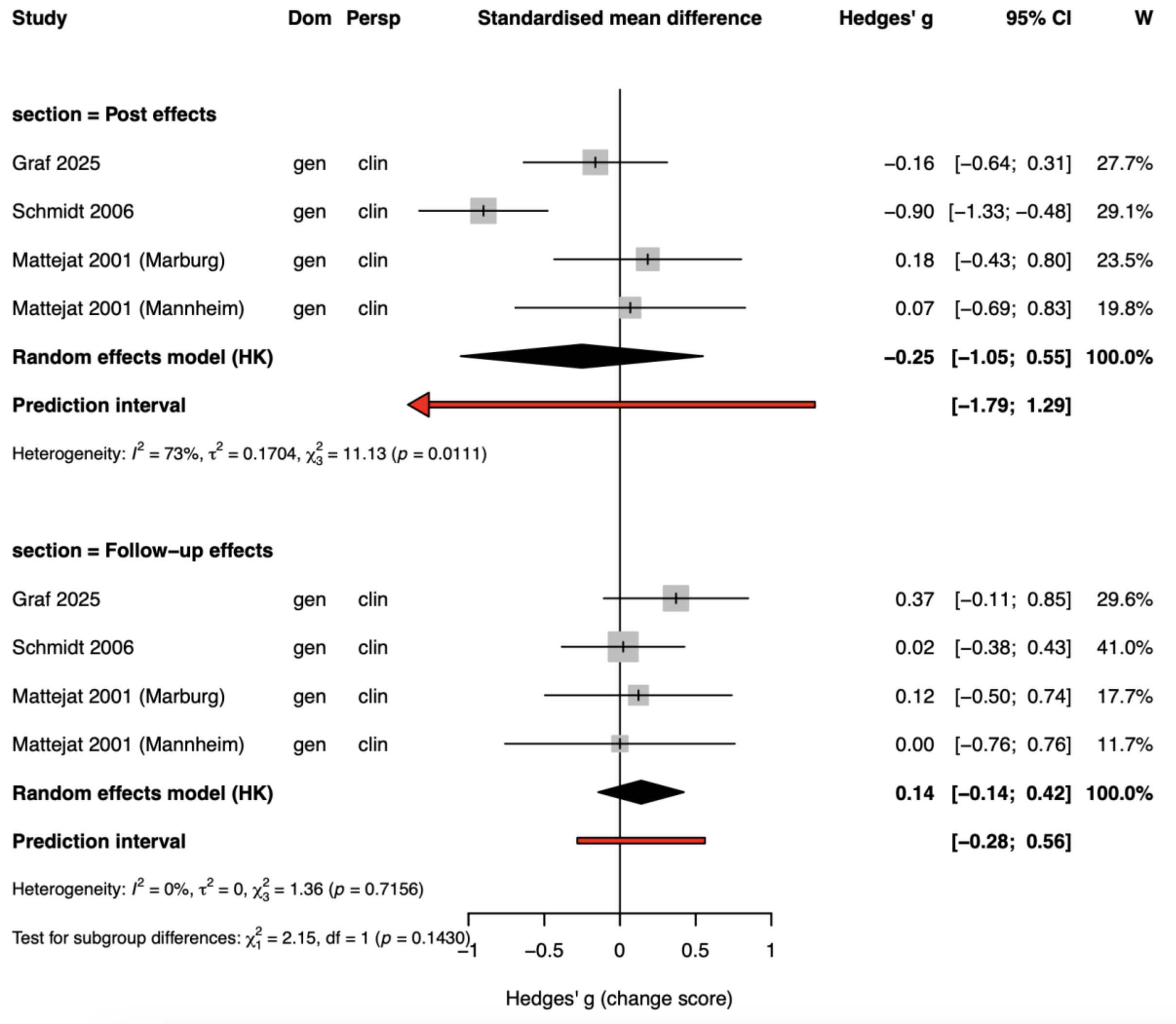

Fig.9: Sensitivity analysis for the outcome Psychopathology (rater- harmonized)

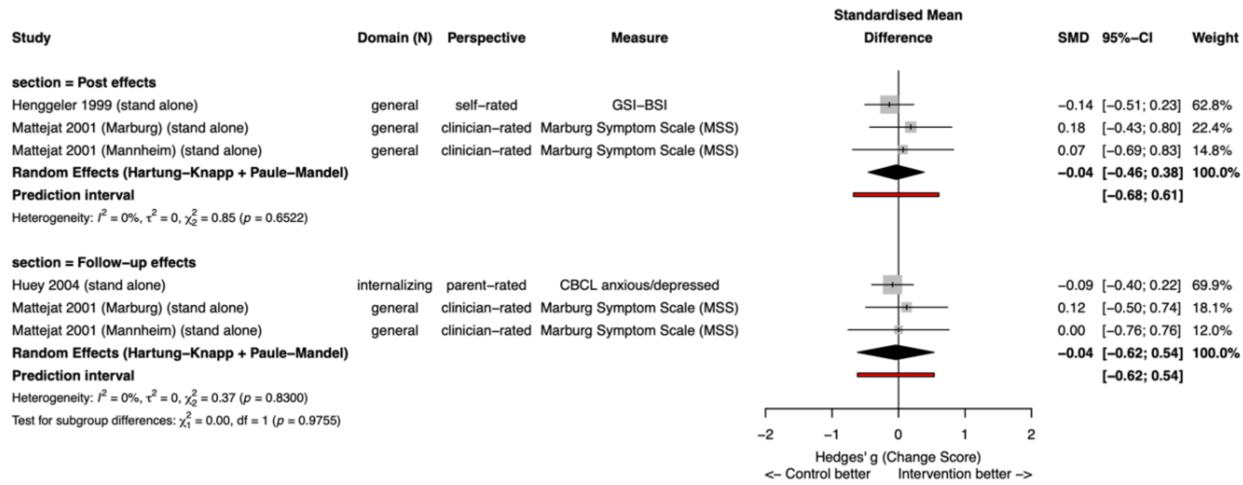

Fig.10: Sensitivity analysis for the outcome Psychopathology (only RCTs)

Article title: Efficacy of home treatment and inpatient treatment for children and adolescents in psychiatric crisis: A systematic review and meta-analysis
Journal: European Child & Adolescent Psychiatry
Authors: Karolina Foremnik, Gaby Sroczynski, Jan Stratil, Marjan Arvandi, Anja Neumann, Barbara Buchberger
Medical Faculty, University of Duisburg-Essen, Germany
Corresponding author (KF)
E-Mail: karolina.foremnik@uni-due.de

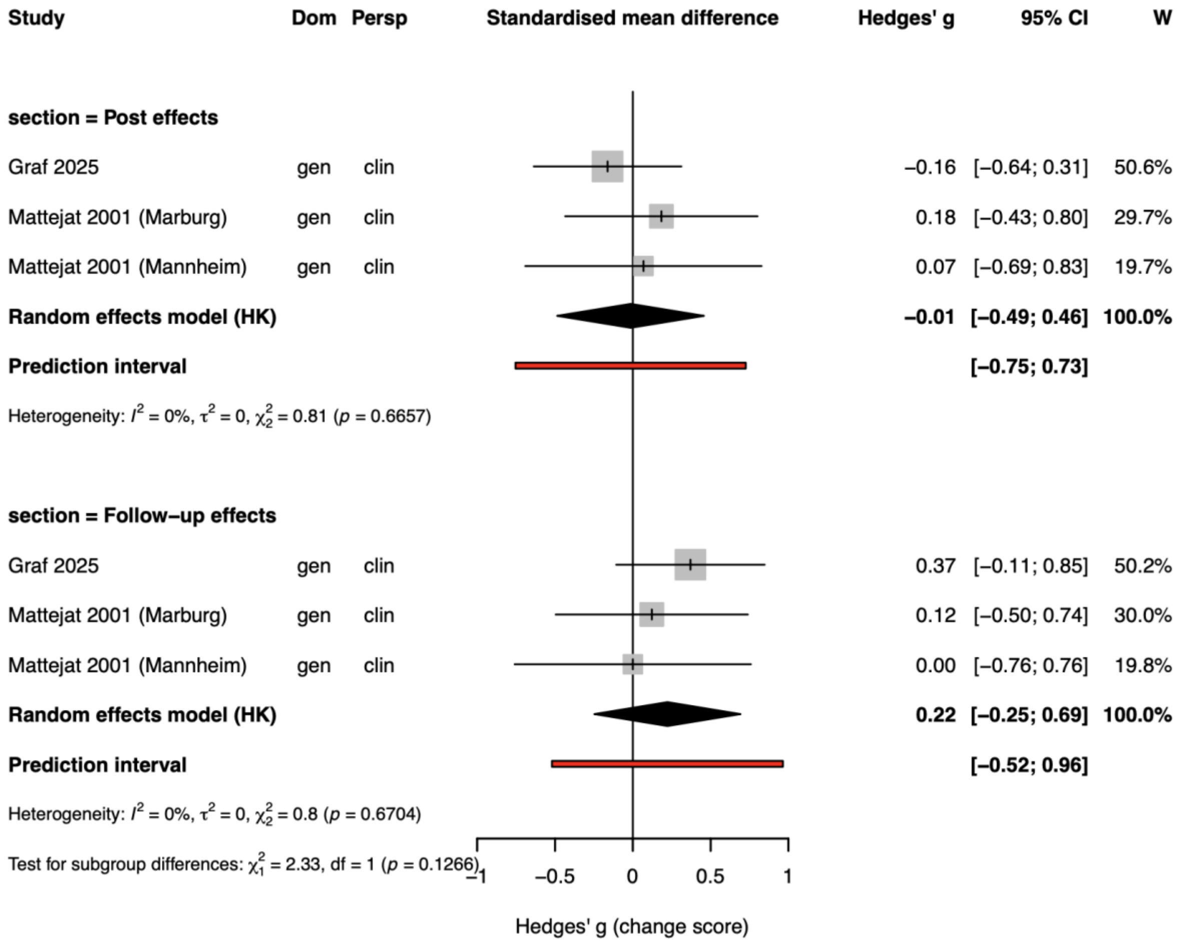

Fig.11: Sensitivity analysis for the outcome Psychopathology (high RoB excluded)

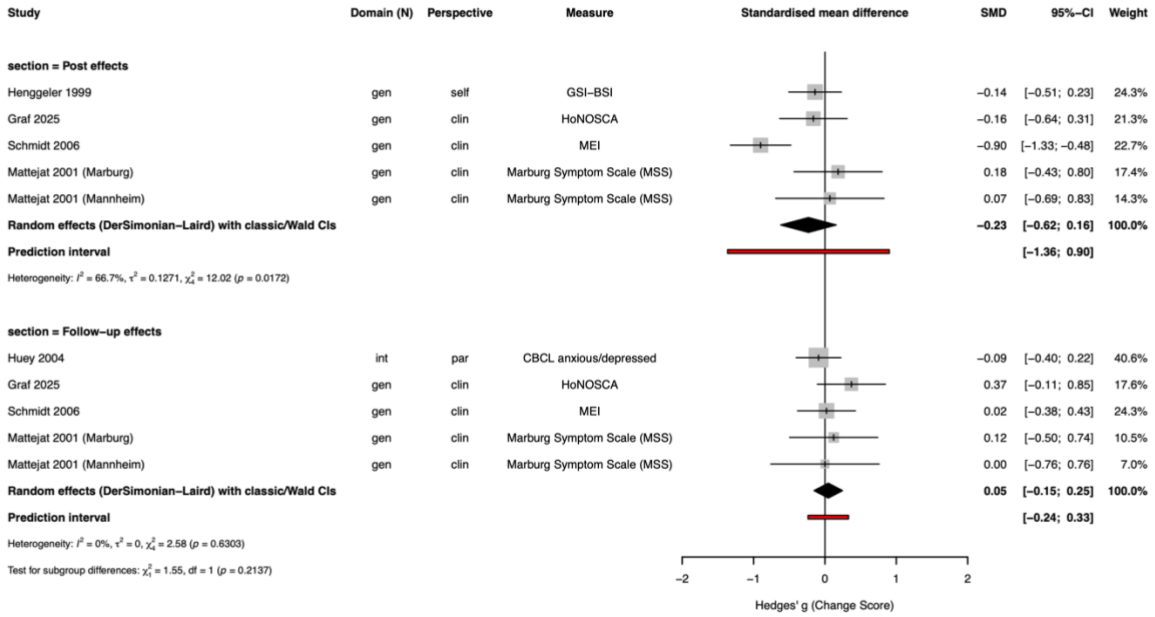

Fig.12: Sensitivity analysis for the outcome Psychopathology (DerSimonian–Laird)

Article title: Efficacy of home treatment and inpatient treatment for children and adolescents in psychiatric crisis: A systematic review and meta-analysis
Journal: European Child & Adolescent Psychiatry
Authors: Karolina Foremnik, Gaby Sroczynski, Jan Stratil, Marjan Arvandi, Anja Neumann, Barbara Buchberger
Medical Faculty, University of Duisburg-Essen, Germany
Corresponding author (KF)
E-Mail: karolina.foremnik@uni-due.de

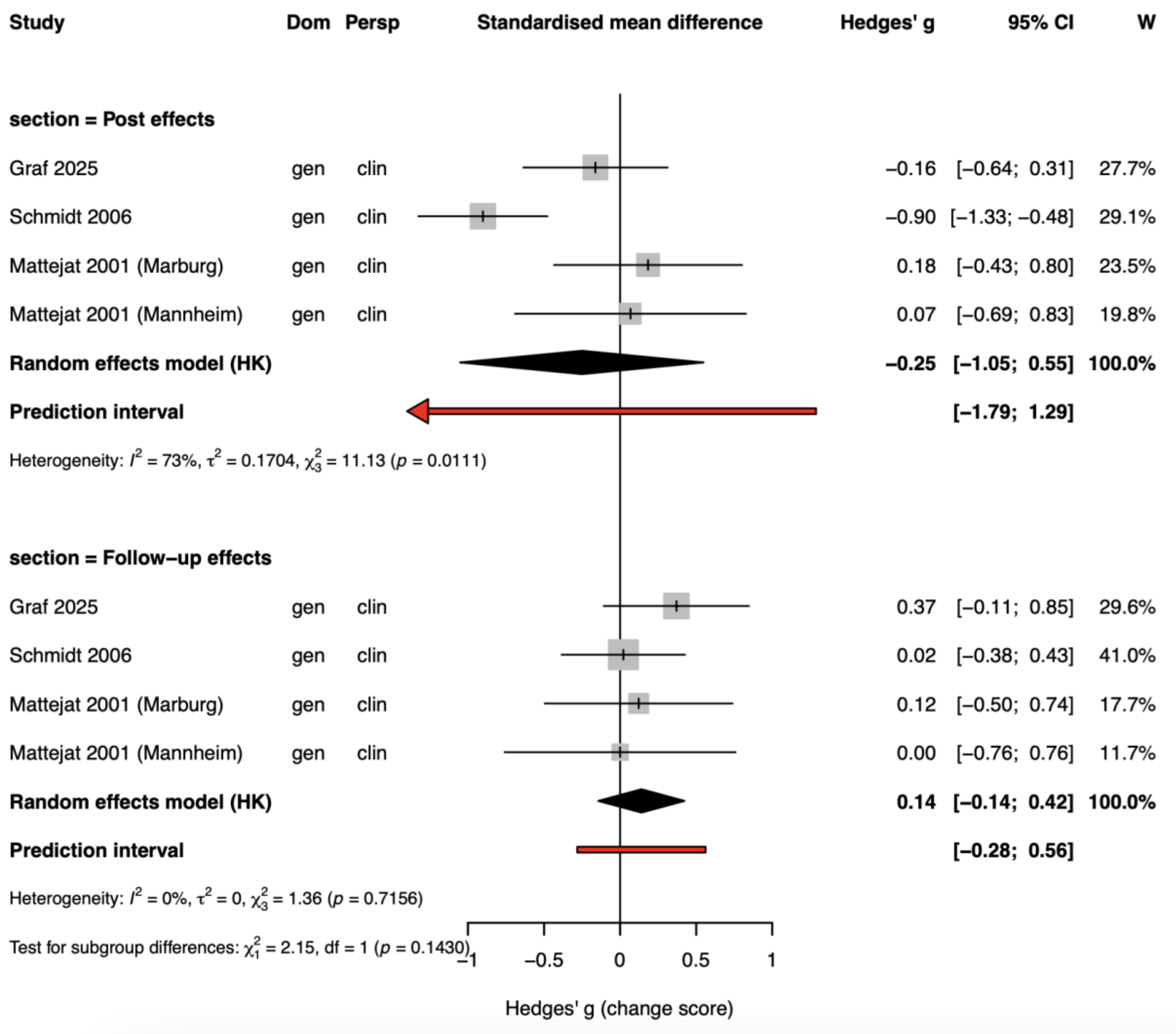

Fig.13: Sensitivity analysis for the outcome Psychopathology (Psychiatric emergencies (under MST treatment) excluded)

Article title: Efficacy of home treatment and inpatient treatment for children and adolescents in psychiatric crisis: A systematic review and meta-analysis
Journal: European Child & Adolescent Psychiatry
Authors: Karolina Foremnik, Gaby Sroczynski, Jan Stratil, Marjan Arvandi, Anja Neumann, Barbara Buchberger
Medical Faculty, University of Duisburg-Essen, Germany
Corresponding author (KF)
E-Mail: karolina.foremnik@uni-due.de

Sensitivity analysis: Readmissions

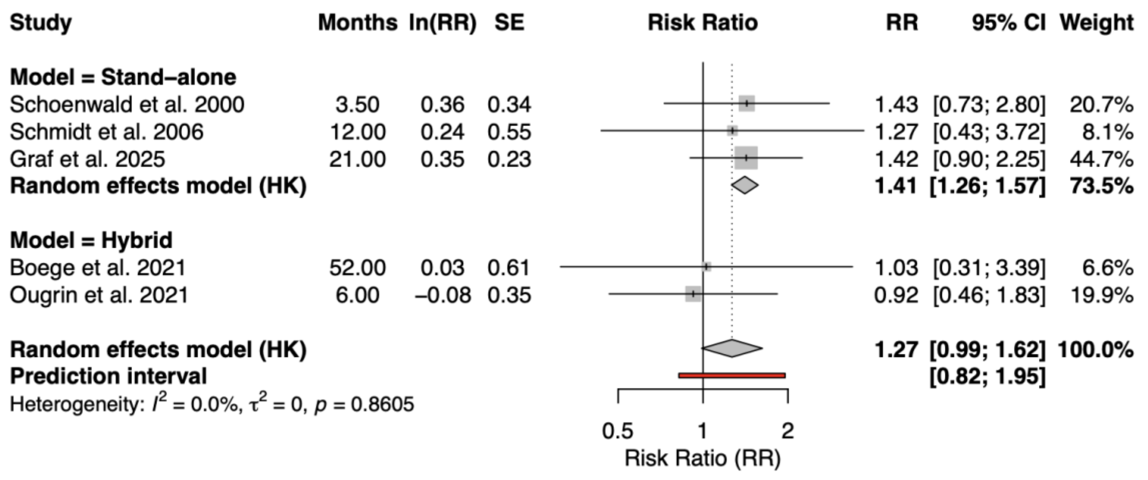

Fig.14: Sensitivity analysis for the outcome Readmissions (Hartung Knapp without Ad hoc variance correction)

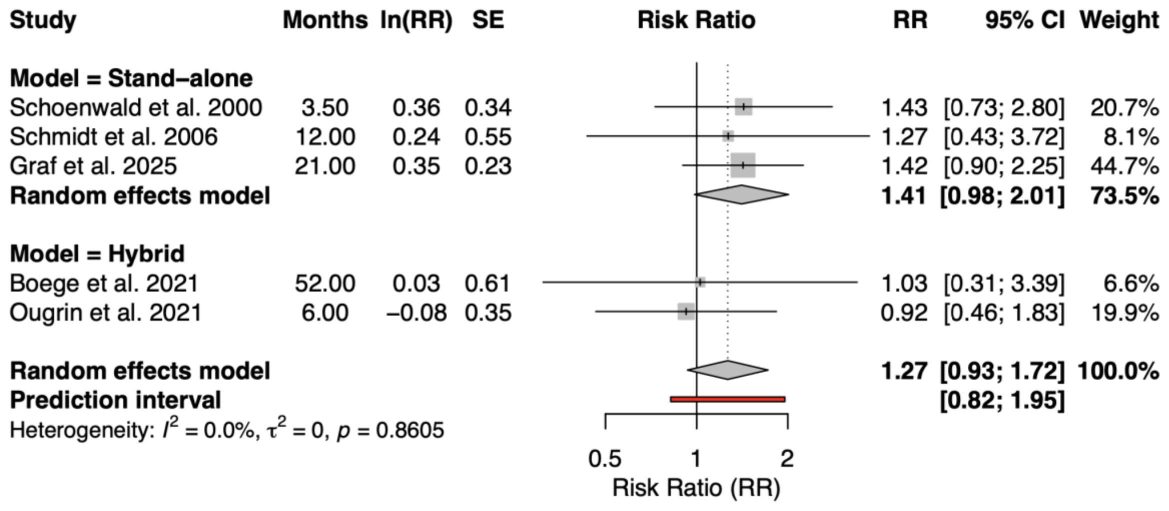

Fig.15: Sensitivity analysis for the outcome Readmissions (DerSimonean-Laird)

Article title: Efficacy of home treatment and inpatient treatment for children and adolescents in psychiatric crisis: A systematic review and meta-analysis
Journal: European Child & Adolescent Psychiatry
Authors: Karolina Foremnik, Gaby Sroczynski, Jan Stratil, Marjan Arvandi, Anja Neumann, Barbara Buchberger
Medical Faculty, University of Duisburg-Essen, Germany
Corresponding author (KF)
E-Mail: karolina.foremnik@uni-due.de

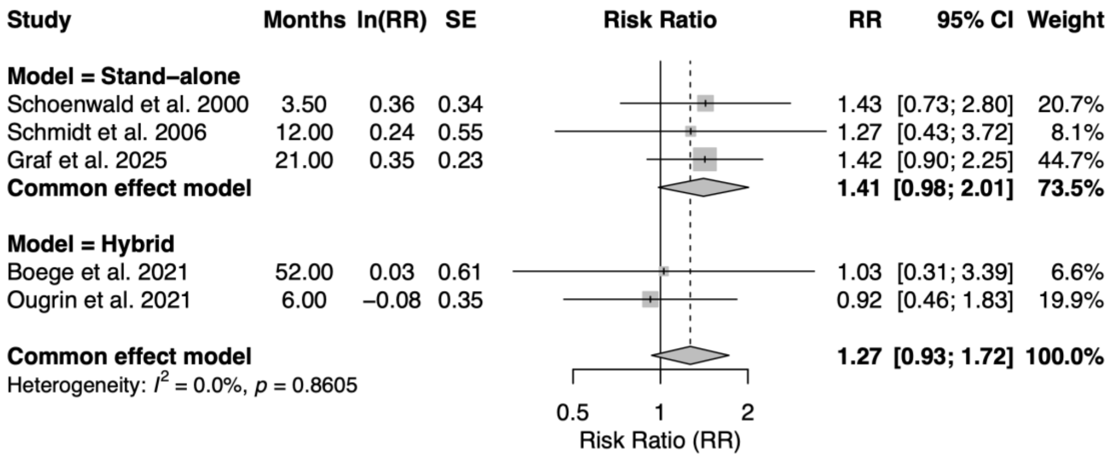

Fig.16: Sensitivity analysis for the outcome Readmissions (Common effects model)

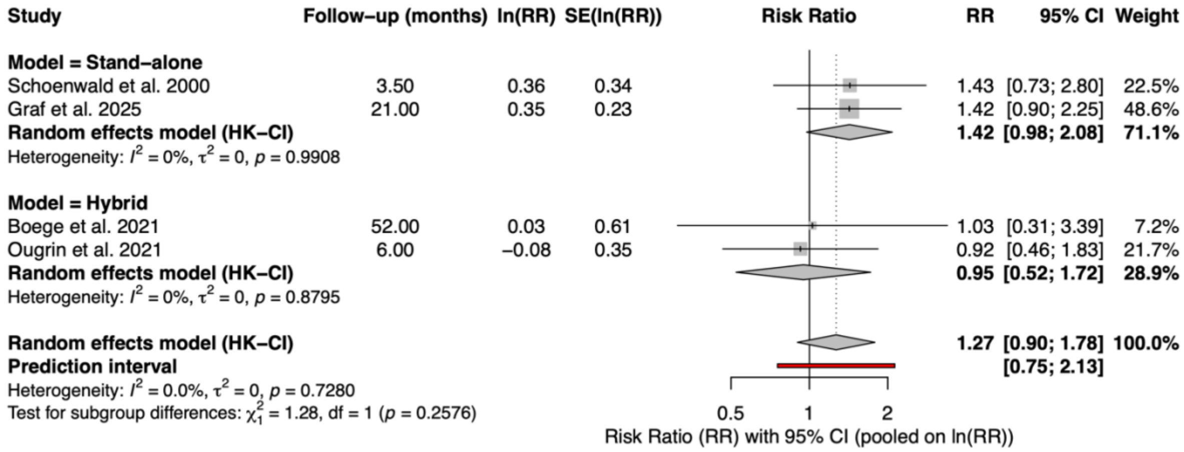

Fig.17: Sensitivity analysis for the outcome Readmissions (high RoB excluded); exploratory pooling of two studies

Authors: Karolina Foremnik, Gaby Sroczynski, Jan Stratil, Marjan Arvandi, Anja Neumann, Barbara Buchberger
Medical Faculty, University of Duisburg-Essen, Germany
Corresponding author (KF)
E-Mail: karolina.foremnik@uni-due.de

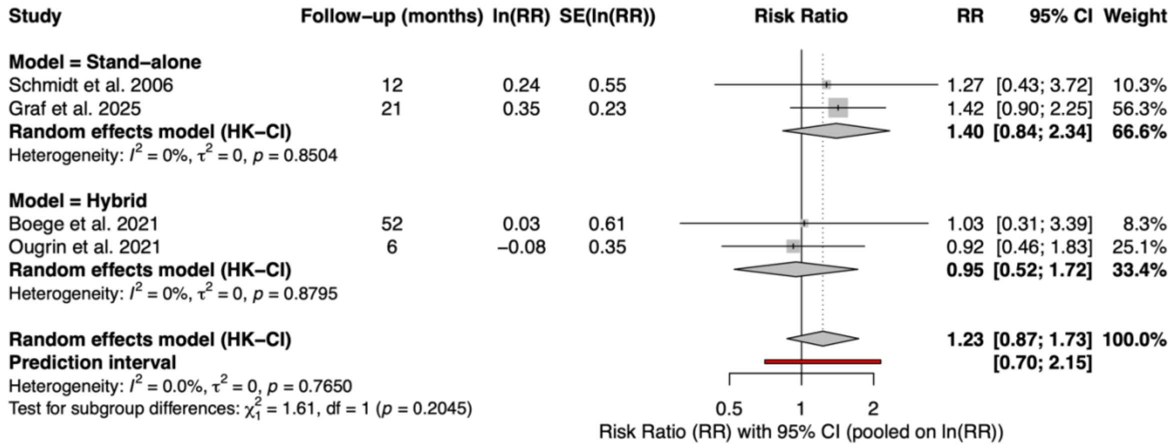

Fig.18: Sensitivity analysis for the outcome Readmissions (Psychiatric emergencies (under MST treatment) excluded); exploratory pooling of two studies

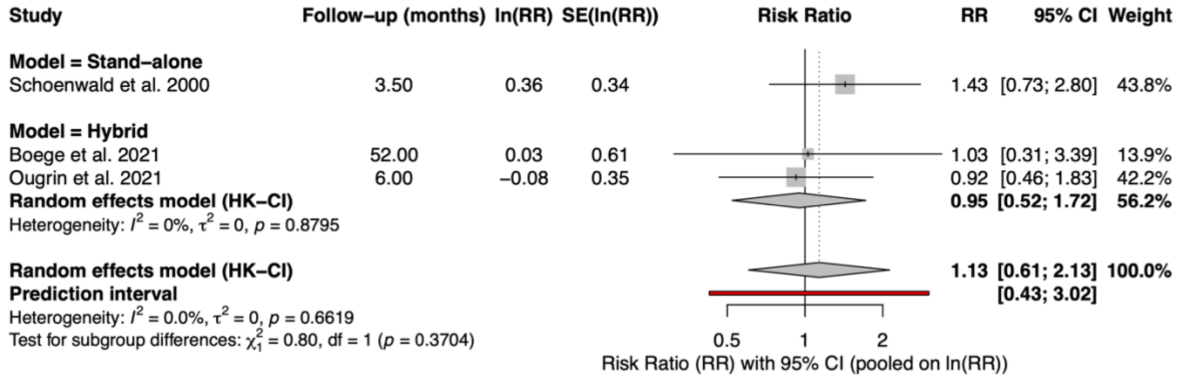

Fig.19: Sensitivity analysis for the outcome Readmissions (only RCTs); exploratory pooling of two studies

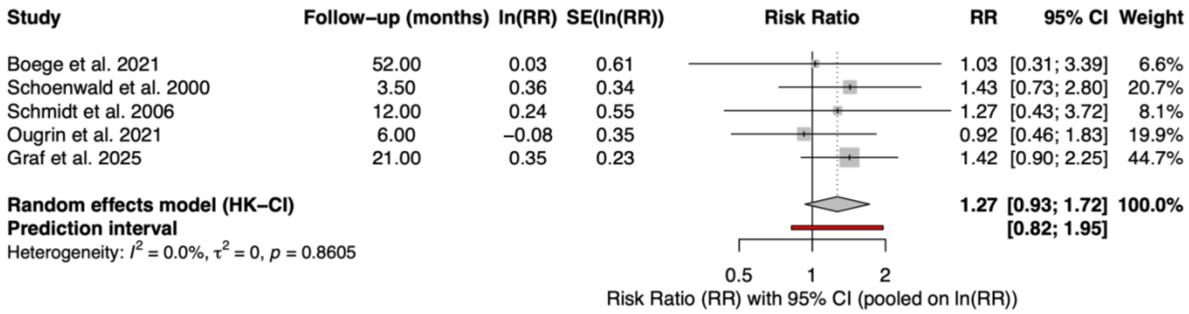

Fig.20: Sensitivity analysis for the outcome Readmissions (hybrid included)
