# Supplementary material for: Efficacy of home treatment and inpatient treatment for children and adolescents in psychiatric crisis: a systematic review and meta-analysis
Source: Eur Child Adolesc Psychiatry. 2026 Jun 1;35(7):2103–27. doi: 10.1007/s00787-026-03060-0 (PMC13427882; doi:10.1007/s00787-026-03060-0)
Supplement: Supplementary file 10 — Supplementary Material 10 [file 787_2026_3060_MOESM10_ESM.pdf]

Article title: **Efficacy of home treatment and inpatient treatment for children and adolescents in psychiatric crisis: A systematic review and meta-analysis**
Journal: European Child & Adolescent Psychiatry
Authors: Karolina Foremnik, Gaby Sroczynski, Jan Stratil, Marjan Arvandi, Anja Neumann, Barbara Buchberger
Medical Faculty, University of Duisburg-Essen, Germany
Corresponding author (KF)
E-Mail: karolina.foremnik@uni-due.de

Exploratory subgroup analyses Psychosocial functioning- stand alone

1) By domains: substance use, social functioning, school/work functioning and overall functioning

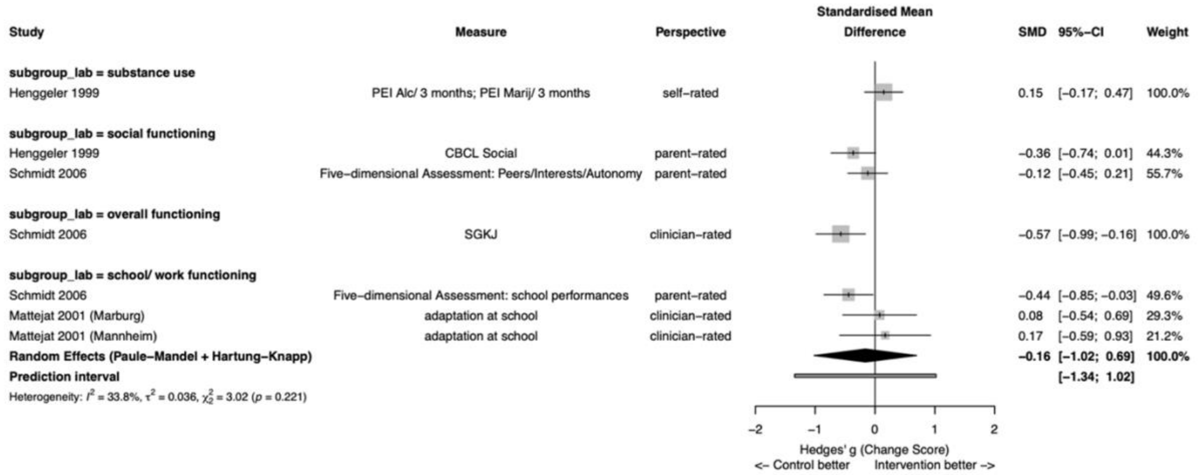

Fig.1: Forest plot of pre-post effect sizes (Hedges' g) for psychosocial functioning-stand alone: Subgroup analysis by domains

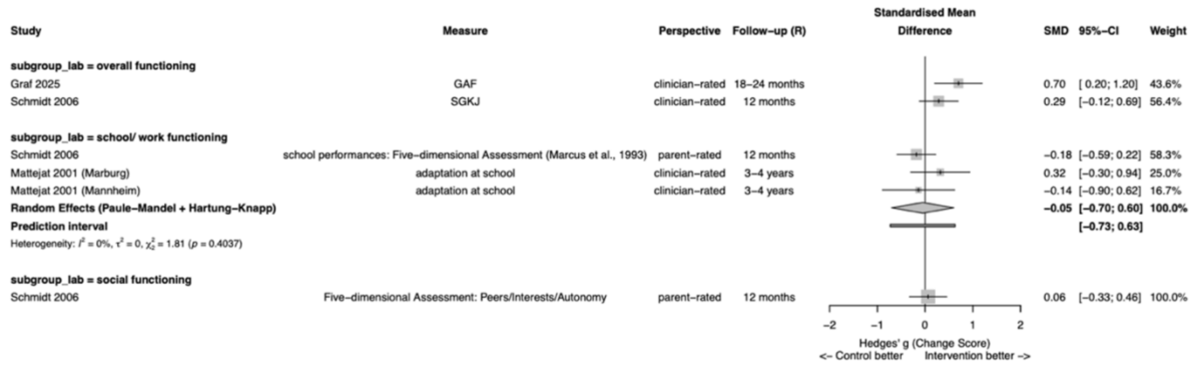

Fig. 2: Forest plot of follow-up effect sizes (Hedges' g) for psychosocial functioning- stand alone: Subgroup analysis by domains

Article title: Efficacy of home treatment and inpatient treatment for children and adolescents in psychiatric crisis: A systematic review and meta-analysis
Journal: European Child & Adolescent Psychiatry
Authors: Karolina Foremnik, Gaby Sroczynski, Jan Stratil, Marjan Arvandi, Anja Neumann, Barbara Buchberger
Medical Faculty, University of Duisburg-Essen, Germany
Corresponding author (KF)
E-Mail: karolina.foremnik@uni-due.de

2) By follow-up duration (> 12 months vs. ≤ 12 months)

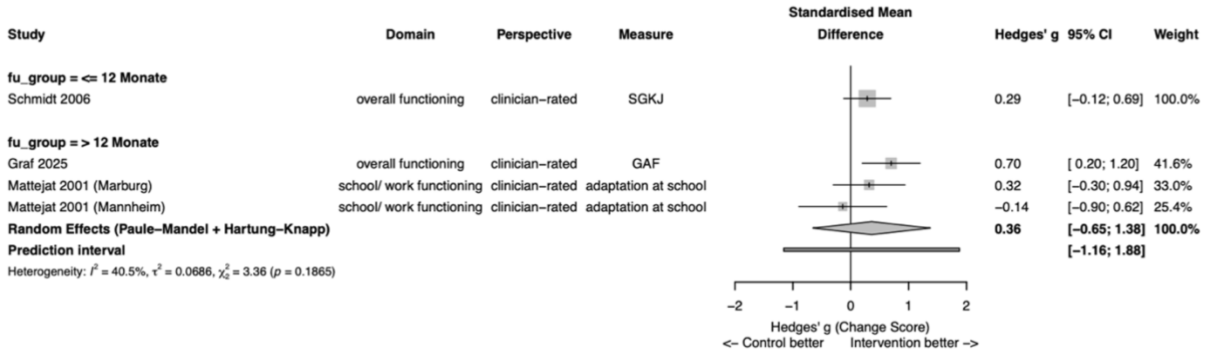

Fig.3: Forest plot of follow-up effect sizes (Hedges' g) for psychosocial functioning- stand alone: Subgroup analysis by follow-up duration

3) By rater perspective (clinician, parent, self-rated)

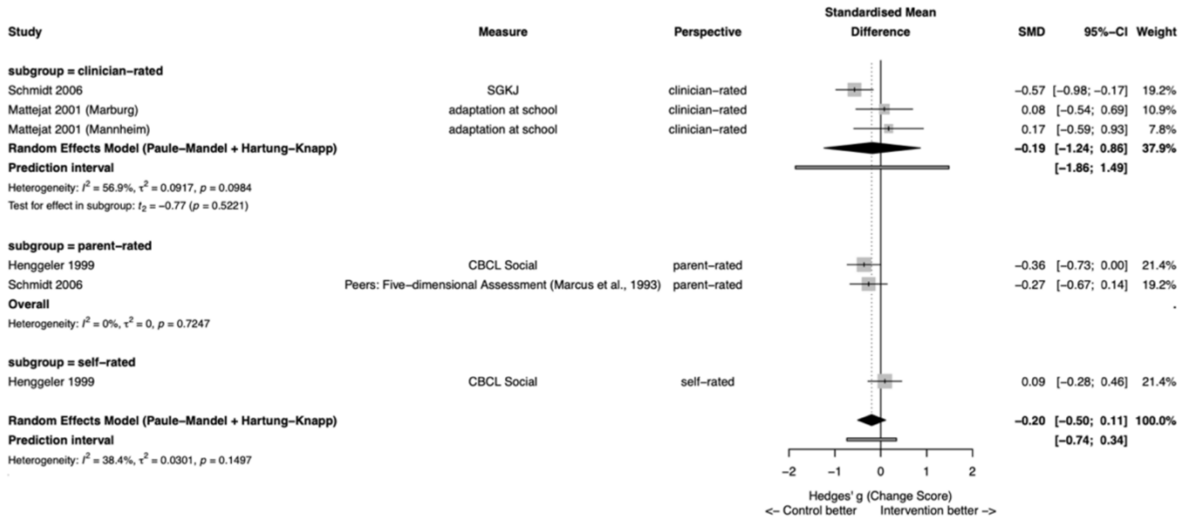

Fig.4: Forest plot of pre-post effect sizes (Hedges' g) for psychosocial functioning-stand alone: Subgroup analysis by rater perspectives

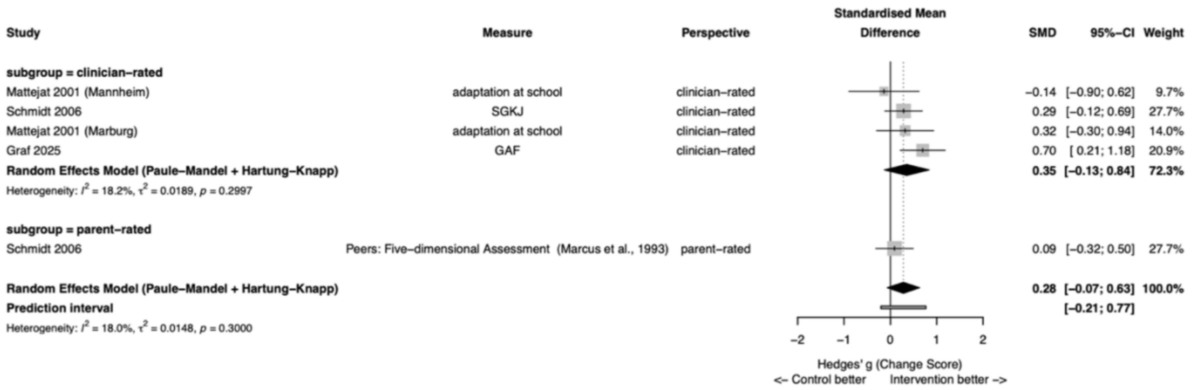

Article title: Efficacy of home treatment and inpatient treatment for children and adolescents in psychiatric crisis: A systematic review and meta-analysis
Journal: European Child & Adolescent Psychiatry
Authors: Karolina Foremnik, Gaby Sroczynski, Jan Stratil, Marjan Arvandi, Anja Neumann, Barbara Buchberger
Medical Faculty, University of Duisburg-Essen, Germany
Corresponding author (KF)
E-Mail: karolina.foremnik@uni-due.de

Fig.5: Forest plot of follow-up effect sizes (Hedges' g) for psychosocial functioning- stand alone: Subgroup analysis by rater perspectives

Article title: Efficacy of home treatment and inpatient treatment for children and adolescents in psychiatric crisis: A systematic review and meta-analysis
Journal: European Child & Adolescent Psychiatry
Authors: Karolina Foremnik, Gaby Sroczynski, Jan Stratil, Marjan Arvandi, Anja Neumann, Barbara Buchberger
Medical Faculty, University of Duisburg-Essen, Germany
Corresponding author (KF)
E-Mail: karolina.foremnik@uni-due.de

Exploratory subgroup analyses Psychopathology- stand alone

1) By domains: internalizing, externalizing and general

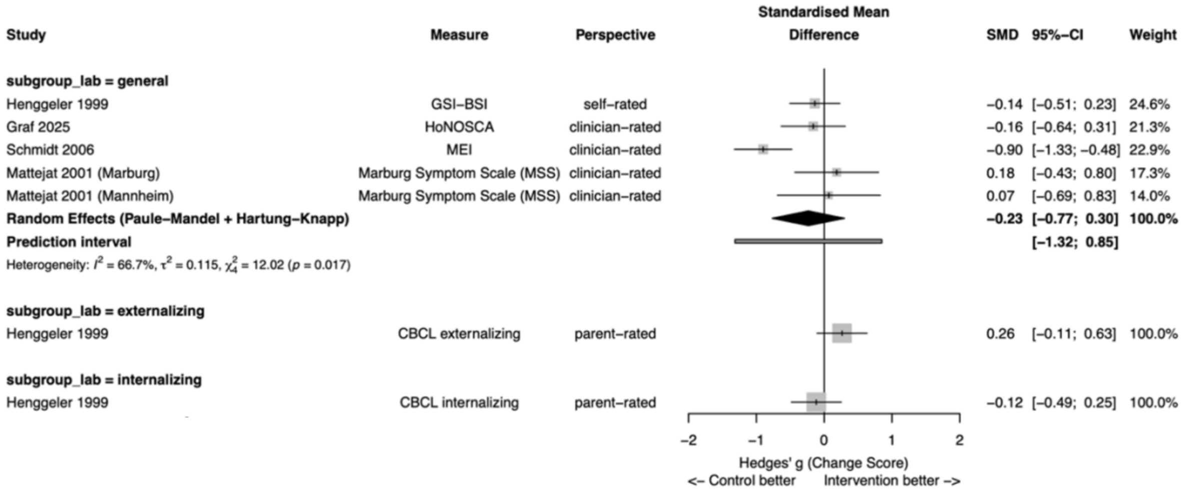

Fig.6: Forest plot of pre-post effect sizes (Hedges' g) for psychopathology-stand alone: Subgroup analysis by symptom domains.

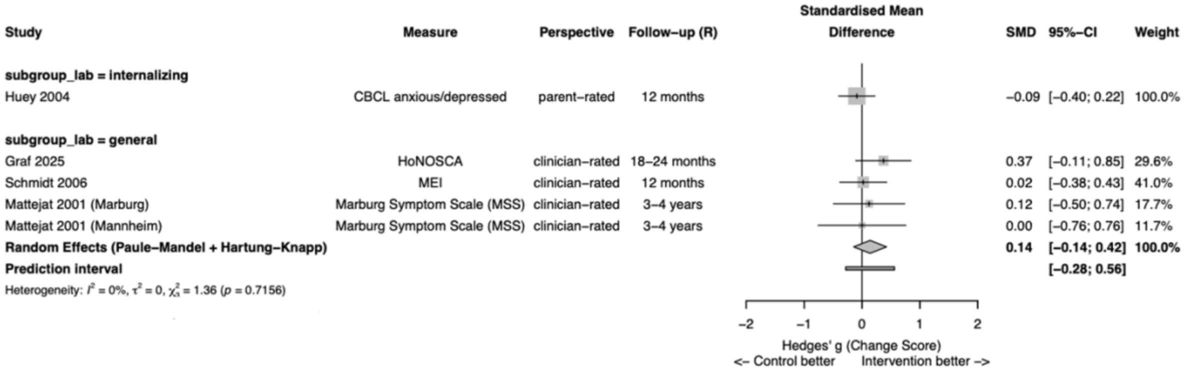

Fig.7: Forest plot of follow-up effect sizes (Hedges' g) for psychopathology- stand alone: Subgroup analysis by symptom domains

Article title: Efficacy of home treatment and inpatient treatment for children and adolescents in psychiatric crisis: A systematic review and meta-analysis
Journal: European Child & Adolescent Psychiatry
Authors: Karolina Foremnik, Gaby Sroczynski, Jan Stratil, Marjan Arvandi, Anja Neumann, Barbara Buchberger
Medical Faculty, University of Duisburg-Essen, Germany
Corresponding author (KF)
E-Mail: karolina.foremnik@uni-due.de

2) By follow-up duration (> 12 months vs. ≤ 12 months)

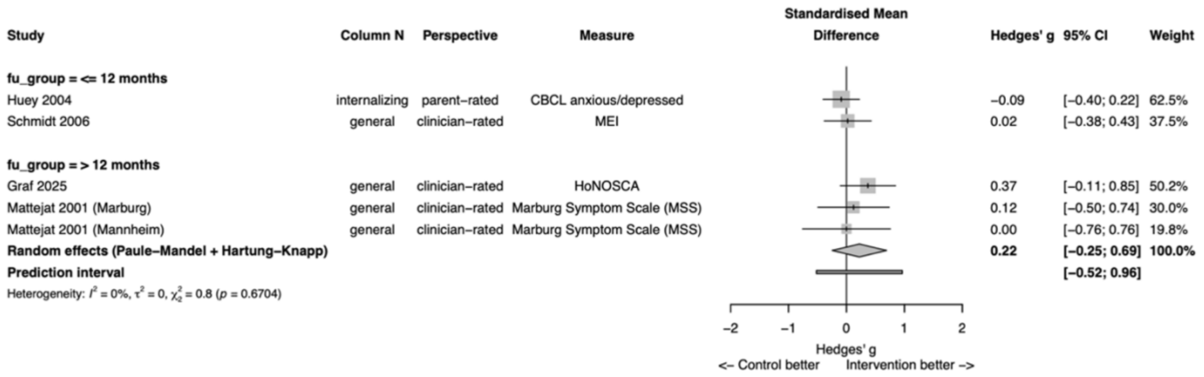

Fig.8: Forest plot of follow-up effect sizes (Hedges' g) for Psychopathology: Subgroup analysis by follow-up duration

3) By rater perspective (clinician, parent, self, teacher-rated)

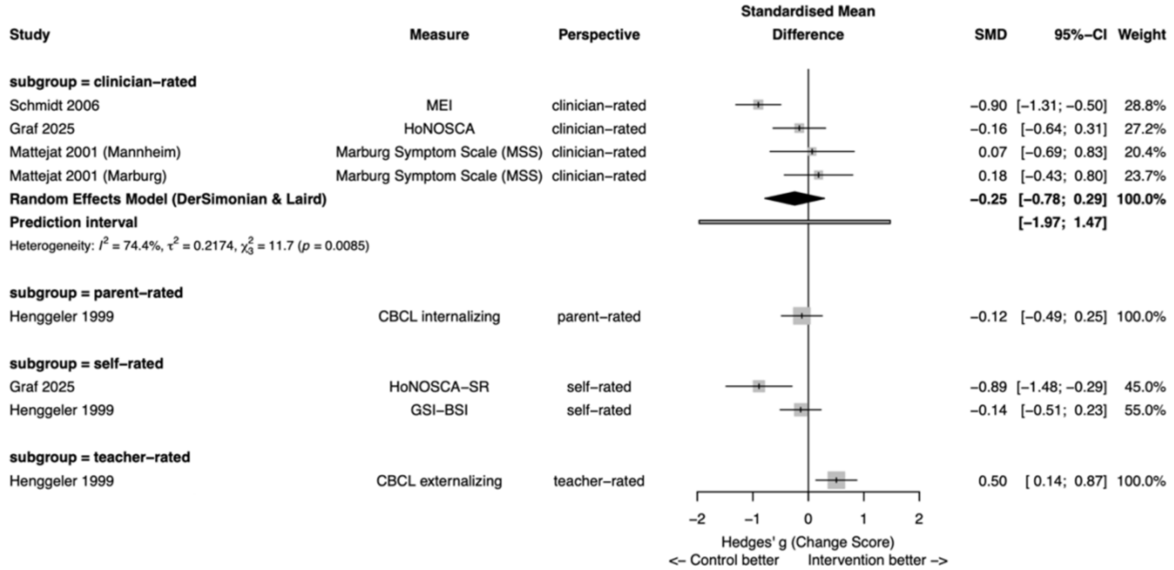

Fig.9: Forest plot of pre-post effect sizes (Hedges' g) for Psychopathology-stand alone: Subgroup analysis by rater perspectives

Article title: Efficacy of home treatment and inpatient treatment for children and adolescents in psychiatric crisis: A systematic review and meta-analysis
Journal: European Child & Adolescent Psychiatry
Authors: Karolina Foremnik, Gaby Sroczynski, Jan Stratil, Marjan Arvandi, Anja Neumann, Barbara Buchberger
Medical Faculty, University of Duisburg-Essen, Germany
Corresponding author (KF)
E-Mail: karolina.foremnik@uni-due.de

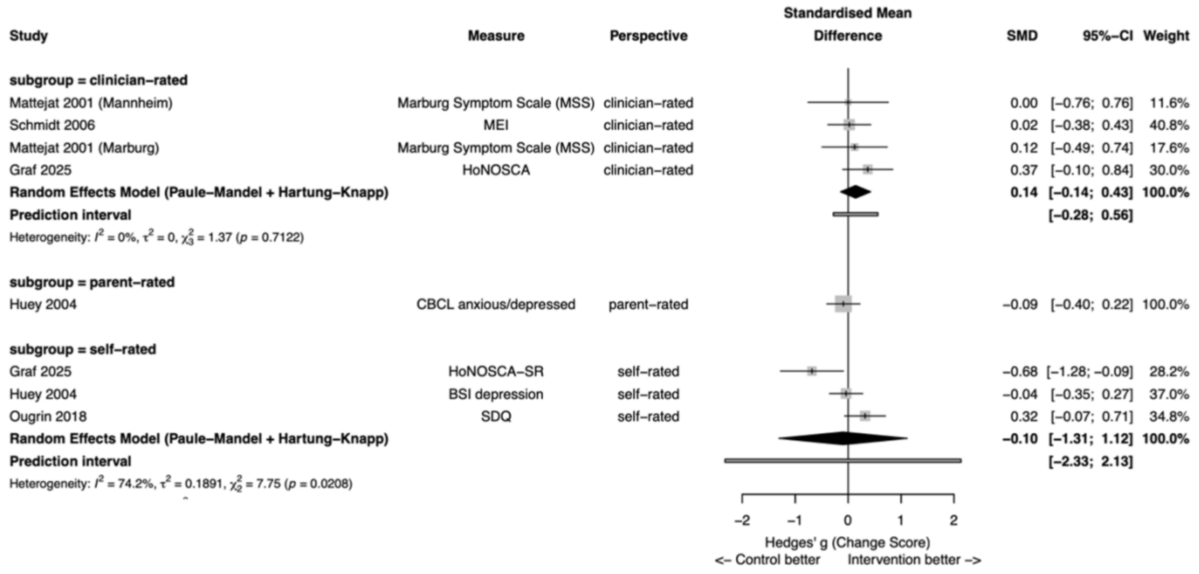

Fig.10: Forest plot of follow-up effect sizes (Hedges' g) for Psychopathology: Subgroup analysis by rater perspectives

Article title: Efficacy of home treatment and inpatient treatment for children and adolescents in psychiatric crisis: A systematic review and meta-analysis
Journal: European Child & Adolescent Psychiatry
Authors: Karolina Foremnik, Gaby Sroczynski, Jan Stratil, Marjan Arvandi, Anja Neumann, Barbara Buchberger
Medical Faculty, University of Duisburg-Essen, Germany
Corresponding author (KF)
E-Mail: karolina.foremnik@uni-due.de

Exploratory subgroup analyses Readmissions

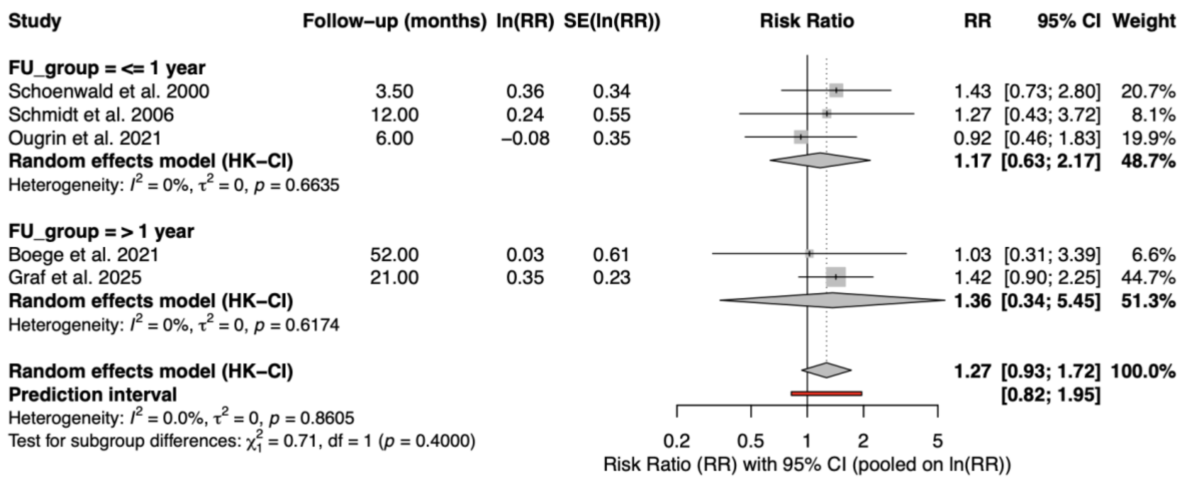

Fig.11: subgroup analysis for the outcome Readmissions based on follow-up duration (≤ 1 year vs. > 1 year); exploratory pooling of two studies
