# Supplementary material for: Efficacy of home treatment and inpatient treatment for children and adolescents in psychiatric crisis: a systematic review and meta-analysis
Source: Eur Child Adolesc Psychiatry. 2026 Jun 1;35(7):2103–27. doi: 10.1007/s00787-026-03060-0 (PMC13427882; doi:10.1007/s00787-026-03060-0)
Supplement: Supplementary file 12 — Supplementary Material 12 [file 787_2026_3060_MOESM12_ESM.pdf]

Article title: Efficacy of home treatment and inpatient treatment for children and adolescents in psychiatric crisis: A systematic review and meta-analysis
Journal: European Child & Adolescent Psychiatry
Authors: Karolina Foremnik, Gaby Sroczynski, Jan Stratil, Marjan Arvandi, Anja Neumann, Barbara Buchberger
Medical Faculty, University of Duisburg-Essen, Germany
Corresponding author (KF)
E-Mail: karolina.foremnik@uni-due.de

OVERALL EFFECTS

a) Overall Psychopathology- stand alone

```
# ================= Psychopathology — Post & Follow-up (ECAP, subgroup_symptoms, non-hybrid only, 8 pt, HK–PM) =================


suppressPackageStartupMessages({
  library(readxl)
  library(meta)
  library(dplyr)
  library(forcats)
  library(grid)
  library(rlang)
  library(stringr)
  library(tibble)
})


options(stringsAsFactors = FALSE)


# ---------------------------- Pfade ----------------------------------------
infile  <- "/Users/karolinaforemnik/Desktop/Promotion/A_Datenextraktion und synthes/Mappe16.xlsx"
insheet <- "meta data"
outfile <- "/Users/karolinaforemnik/Desktop/Promotion/A_Datenextraktion und synthes/Forest_PSY_Post_FU_ECAP_subgroupSymptoms_NONHYBRID_8pt_HKPM.pdf"


# ---------------------------- Helpers ---------------------------------------
numify <- function(x) {
  if (is.null(x)) return(NA_real_)
```

Article title: Efficacy of home treatment and inpatient treatment for children and adolescents in psychiatric crisis: A systematic review and meta-analysis
Journal: European Child & Adolescent Psychiatry
Authors: Karolina Foremnik, Gaby Sroczynski, Jan Stratil, Marjan Arvandi, Anja Neumann, Barbara Buchberger
Medical Faculty, University of Duisburg-Essen, Germany
Corresponding author (KF)
E-Mail: karolina.foremnik@uni-due.de

```
  x_chr <- as.character(x)

  x_chr <- gsub(",", ".", x_chr, fixed = TRUE)

  suppressWarnings(as.numeric(x_chr))

}



letter_to_index <- function(s) {

  s <- toupper(gsub("[^A-Z]", "", s))

  chars <- strsplit(s, "")[[1]]

  idx <- 0L

  for (ch in chars) idx <- idx * 26L + match(ch, LETTERS)

  idx

}



col_by_letter <- function(df, letter) {

  i <- letter_to_index(letter)

  if (!is.na(i) && i >= 1L && i <= ncol(df)) df[[i]] else rep(NA, nrow(df))

}



safe_chr_col <- function(df, candidates) {

  for (nm in candidates) if (nm %in% names(df)) return(as.character(df[[nm]]))

  rep(NA_character_, nrow(df))

}



# ---------------------------- Daten laden -----------------------------------

raw <- read_excel(infile, sheet = insheet)



# Model type (Spalte C)
```

Authors: Karolina Foremnik, Gaby Sroczynski, Jan Stratil, Marjan Arvandi, Anja Neumann, Barbara Buchberger
Medical Faculty, University of Duisburg-Essen, Germany
Corresponding author (KF)
E-Mail: karolina.foremnik@uni-due.de

```
modeltype_raw <- col_by_letter(raw, "C")

modeltype <- tolower(trimws(as.character(modeltype_raw)))

is_hybrid <- !is.na(modeltype) & modeltype == "hybrid"


# Studienlabel NUR aus Spalte A

lab_A <- suppressWarnings(as.character(col_by_letter(raw, "A")))

studylab <- ifelse(!is.na(lab_A) & nzchar(lab_A),

           lab_A,

           paste0("Study ", seq_len(nrow(raw))))


Outcome_raw <- col_by_letter(raw, "D")

Domain_raw  <- col_by_letter(raw, "N")

Time_raw    <- col_by_letter(raw, "P")

g_raw       <- col_by_letter(raw, "AJ")


N_IG_pre <- if ("N_IG (pre)" %in% names(raw))

  numify(raw[["N_IG (pre)"]]) else numify(col_by_letter(raw, "V"))

N_CG_pre <- if ("N_CG (pre)" %in% names(raw))

  numify(raw[["N_CG (pre)"]]) else numify(col_by_letter(raw, "W"))


measure_col     <- safe_chr_col(raw, c("Measure ", "Measure"))

perspective_col <- safe_chr_col(raw, c("Perspective", "perspective"))


subgroup_symptoms_raw <- safe_chr_col(

  raw,

  c("subgroup_symptoms", "Subgroup_symptoms", "subgroup symptoms", "Subgroup
symptoms")
```

Authors: Karolina Foremnik, Gaby Sroczynski, Jan Stratil, Marjan Arvandi, Anja Neumann, Barbara Buchberger
Medical Faculty, University of Duisburg-Essen, Germany
Corresponding author (KF)
E-Mail: karolina.foremnik@uni-due.de

```
)


# ---------------------------- Aufbereiten/Filtern ---------------------------
base_dat <- tibble(
 studyid       = studylab,
 Outcome       = as.character(Outcome_raw),
 DomainN       = as.character(Domain_raw),
 TimeRaw       = as.character(Time_raw),
 g             = numify(g_raw),
 n1            = N_IG_pre,
 n2            = N_CG_pre,
 measure       = measure_col,
 perspective   = perspective_col,
 modeltype     = modeltype,
 is_hybrid     = is_hybrid,
 subgroup_symptoms = subgroup_symptoms_raw
) %>%
 mutate(
  Outcome_l   = tolower(trimws(ifelse(is.na(Outcome), "", Outcome))),
  flag_outcome = grepl("psychopath", Outcome_l)
 ) %>%
 filter(flag_outcome) %>%
 mutate(DomainN_trim = trimws(ifelse(is.na(DomainN), "", DomainN))) %>%
 filter(nzchar(DomainN_trim)) %>%
 mutate(
  subgroup_symptoms_clean =
   trimws(ifelse(is.na(subgroup_symptoms), "", subgroup_symptoms))
```

Authors: Karolina Foremnik, Gaby Sroczynski, Jan Stratil, Marjan Arvandi, Anja Neumann, Barbara Buchberger
Medical Faculty, University of Duisburg-Essen, Germany
Corresponding author (KF)
E-Mail: karolina.foremnik@uni-due.de

```
) %>%
filter(nzchar(subgroup_symptoms_clean)) %>%
mutate(
  Time_l = tolower(trimws(ifelse(is.na(TimeRaw), "", TimeRaw))),
  section = case_when(
    grepl("^post$", Time_l) ~ "Post effects",
    grepl("follow", Time_l) | grepl("long\\s*term", Time_l) ~ "Follow-up effects",
    TRUE ~ NA_character_
  )
) %>%
filter(!is.na(section)) %>%
filter(is.finite(g), is.finite(n1), is.finite(n2), n1 > 1, n2 > 1, (n1 + n2 - 2) > 0) %>%
mutate(
  v_g  = (n1 + n2)/(n1*n2) + (g^2)/(2*(n1 + n2 - 2)),
  sei  = sqrt(v_g),
  perspective_clean = tolower(trimws(perspective)),
  perspective_clean = dplyr::recode(
    perspective_clean,
    "clinician-rated" = "clin",
    "self-rated"      = "self",
    "parent-rated"    = "par",
    .default          = ifelse(
      is.na(perspective_clean) | !nzchar(perspective_clean),
      "n/a",
      perspective_clean
    )
  ),
```

Authors: Karolina Foremnik, Gaby Sroczynski, Jan Stratil, Marjan Arvandi, Anja Neumann, Barbara Buchberger
Medical Faculty, University of Duisburg-Essen, Germany
Corresponding author (KF)
E-Mail: karolina.foremnik@uni-due.de

```
    domain_clean = tolower(DomainN_trim),
    domain_clean = dplyr::recode(
      domain_clean,
      "general"      = "gen",
      "internalizing" = "int",
      "externalizing" = "ext",
      .default       = domain_clean
    )
  ) %>%
  filter(is.finite(sei) & sei > 0)

if (nrow(base_dat) == 0) stop("Keine Daten nach Filter (inkl. subgroup_symptoms).")

# ---------------------------- nur non-hybrid --------------------------------
dat_main <- base_dat %>% filter(!is_hybrid)

section_order <- c("Post effects", "Follow-up effects")
if (nrow(dat_main) > 0)
  dat_main$section <- factor(dat_main$section, levels = section_order)

# ---------------------------- Meta (HK–PM) ---------------------------------
m_main <- NULL
if (nrow(dat_main) > 0) {
  m_main <- metagen(
    TE   = dat_main$g,
    seTE = dat_main$sei,
    studlab = dat_main$studyid,
```

Authors: Karolina Foremnik, Gaby Sroczynski, Jan Stratil, Marjan Arvandi, Anja Neumann, Barbara Buchberger
Medical Faculty, University of Duisburg-Essen, Germany
Corresponding author (KF)
E-Mail: karolina.foremnik@uni-due.de

```
    data   = dat_main,
    sm     = "SMD",
    method.tau     = "PM",
    method.random.ci = "HK",
    random  = TRUE,
    common  = FALSE,
    subgroup = dat_main$section,
    prediction.subgroup = TRUE,
    keepdata = TRUE
  )

  m_main$domain_clean      <- dat_main$domain_clean
  m_main$perspective_clean <- dat_main$perspective_clean

  # Über den Punktschätzern: jetzt SMD
  m_main$sm <- "Standardised mean difference"
}

# ----------------------------- Forest 174 × 234 mm ----------------------------
pdf(outfile, width = 174/25.4, height = 200/25.4)

par(mai = c(1.0, 1.2, 0.8, 0.2))

if (!is.null(m_main)) {
  forest(
    m_main,
    overall          = FALSE,
```

Authors: Karolina Foremnik, Gaby Sroczynski, Jan Stratil, Marjan Arvandi, Anja Neumann, Barbara Buchberger
Medical Faculty, University of Duisburg-Essen, Germany
Corresponding author (KF)
E-Mail: karolina.foremnik@uni-due.de

```
overall.hetstat    = FALSE,
prediction         = TRUE,
prediction.subgroup = TRUE,
print.byvar        = TRUE,
print.subgroup.labels = TRUE,
bylab              = "",

leftcols  = c("studlab","domain_clean","perspective_clean"),
leftlabs  = c("Study","Dom","Persp"),

# ⬅ mittlere Spaltenüberschrift jetzt Hedges' g
rightcols = c("effect","ci","w.random"),
rightlabs = c("Hedges' g","95% CI","W"),

print.I2   = TRUE,
print.tau2 = TRUE,
print.Q    = TRUE,

fontsize     = 8,
spacing      = 1.35,
squaresize   = 0.6,
col.diamond  = "black",
col.diamond.lines = "black",
colgap.forest = "1.0mm",

fs.hetstat       = 7,
fs.test.subgroup  = 7,
```

Authors: Karolina Foremnik, Gaby Sroczynski, Jan Stratil, Marjan Arvandi, Anja Neumann, Barbara Buchberger
Medical Faculty, University of Duisburg-Essen, Germany
Corresponding author (KF)
E-Mail: karolina.foremnik@uni-due.de

```
    xlim        = c(-1.4, 1.4),

    # ⬅️ Achsenlabel wieder zurück zu Hedges' g
    xlab        = "Hedges' g (change score)",

    main        = "Psychopathology — Post & Follow-up (non-hybrid pooled, HK–PM)\n(only rows marked in 'subgroup_symptoms')"
  )
} else {
  plot.new()
  title("Keine non-hybrid Studien nach Filter vorhanden")
}

dev.off()

cat("\nFertig. Forest-Plot gespeichert:\n", outfile, "\n")
```

Authors: Karolina Foremnik, Gaby Sroczynski, Jan Stratil, Marjan Arvandi, Anja Neumann, Barbara Buchberger
Medical Faculty, University of Duisburg-Essen, Germany
Corresponding author (KF)
E-Mail: karolina.foremnik@uni-due.de

b) Overall Psychosocial functioning - stand alone

```
# ---------------------------- Pakete ----------------------------------------

suppressPackageStartupMessages({
  library(readxl)
  library(meta)
  library(dplyr)
  library(stringr)
  library(tibble)
})

options(stringsAsFactors = FALSE)

# ---------------------------- Pfade -----------------------------------------
infile  <- "/Users/karolinaforemnik/Desktop/Promotion/A_Datenextraktion und synthes/Mappe16.xlsx"
# infile <- "/mnt/data/Mappe16.xlsx"  # im Chat-Notebook verwenden
insheet <- "meta data"
outfile <- "/Users/karolinaforemnik/Desktop/Promotion/A_Datenextraktion und synthes/Forest_YSF_Post_FU_PMHK_OnePlot.pdf"

# ---------------------------- Helper-Funktionen -----------------------------
numify <- function(x) {
  if (is.null(x)) return(NA_real_)
  x_chr <- as.character(x)
  x_chr <- gsub(",", ".", x_chr, fixed = TRUE)
  suppressWarnings(as.numeric(x_chr))
}
```

Article title: Efficacy of home treatment and inpatient treatment for children and adolescents in psychiatric crisis: A systematic review and meta-analysis
Journal: European Child & Adolescent Psychiatry
Authors: Karolina Foremnik, Gaby Sroczynski, Jan Stratil, Marjan Arvandi, Anja Neumann, Barbara Buchberger
Medical Faculty, University of Duisburg-Essen, Germany
Corresponding author (KF)
E-Mail: karolina.foremnik@uni-due.de

```
letter_to_index <- function(s) {

  s <- toupper(gsub("[^A-Z]", "", s))

  chars <- strsplit(s, "")[[1]]

  idx <- 0L

  for (ch in chars) idx <- idx * 26L + match(ch, LETTERS)

  idx

}


col_by_letter <- function(df, letter) {

  i <- letter_to_index(letter)

  if (!is.na(i) && i >= 1L && i <= ncol(df)) df[[i]] else rep(NA, nrow(df))

}


safe_chr_col <- function(df, candidates) {

  for (nm in candidates) if (!is.null(df[[nm]])) return(as.character(df[[nm]]))

  rep(NA_character_, nrow(df))

}


# ---------------------------- Domain & Perspective Abkürzungen ----------------

abbr_domain <- function(x) {

  xl <- tolower(trimws(x))

  d_short <- case_when(

    grepl("^social", xl) & grepl("function", xl)      ~ "Social f.",

    grepl("^overall", xl) & grepl("function", xl)     ~ "Overall f.",

    grepl("school",  xl) & grepl("function", xl)      ~ "School f.",

    TRUE ~ x
```

Authors: Karolina Foremnik, Gaby Sroczynski, Jan Stratil, Marjan Arvandi, Anja Neumann, Barbara Buchberger
Medical Faculty, University of Duisburg-Essen, Germany
Corresponding author (KF)
E-Mail: karolina.foremnik@uni-due.de

```
  )
  d_short
}


abbr_persp <- function(x) {
  xl <- tolower(trimws(x))
  p_short <- case_when(
    grepl("clin", xl) ~ "clin",
    grepl("self", xl) ~ "self",
    grepl("par",  xl) ~ "par",
    TRUE            ~ "n/a"
  )
  p_short
}


# ---------------------------- Daten laden -----------------------------------
raw <- read_excel(infile, sheet = insheet)


# Model type (Spalte C) -> hybrid vs. nicht-hybrid
modeltype_raw <- col_by_letter(raw, "C")
modeltype <- tolower(trimws(as.character(modeltype_raw)))
is_hybrid <- !is.na(modeltype) & modeltype == "hybrid"


# Studienlabel (A + C)
lab_A <- as.character(col_by_letter(raw, "A"))
lab_C <- as.character(col_by_letter(raw, "C"))
```

Authors: Karolina Foremnik, Gaby Sroczynski, Jan Stratil, Marjan Arvandi, Anja Neumann, Barbara Buchberger
Medical Faculty, University of Duisburg-Essen, Germany
Corresponding author (KF)
E-Mail: karolina.foremnik@uni-due.de

```
studylab <- ifelse(!is.na(lab_A) & nzchar(lab_A),
        ifelse(!is.na(lab_C) & nzchar(lab_C), paste0(lab_A, " (", lab_C, ")"), lab_A),
        paste0("Study ", seq_len(nrow(raw))))

# "(stand alone)" überall entfernen
studylab <- gsub("\\(stand[- ]*alone\\)", "", studylab, ignore.case = TRUE)
studylab <- trimws(studylab)

# Kerndaten-Spalten
Outcome_raw <- col_by_letter(raw, "D")   # Outcome
Domain_raw  <- col_by_letter(raw, "N")   # Domain
Time_raw    <- col_by_letter(raw, "P")   # Zeitpunkt
g_raw       <- col_by_letter(raw, "AJ")  # Hedges' g

# Gruppengrößen
n1_raw <- numify(col_by_letter(raw, "V"))  # N_IG
n2_raw <- numify(col_by_letter(raw, "W"))  # N_CG

# Perspektive
perspective_col <- safe_chr_col(raw, c("Perspective", "perspective"))

# ---------------------------- Aufbereitung / Filter -------------------------
df0 <- tibble(
  studyid    = studylab,
  Outcome    = as.character(Outcome_raw),
  DomainN    = as.character(Domain_raw),
  TimeRaw    = as.character(Time_raw),
```

Authors: Karolina Foremnik, Gaby Sroczynski, Jan Stratil, Marjan Arvandi, Anja Neumann, Barbara Buchberger
Medical Faculty, University of Duisburg-Essen, Germany
Corresponding author (KF)
E-Mail: karolina.foremnik@uni-due.de

```
  g        = numify(g_raw),
  n1        = n1_raw,
  n2        = n2_raw,
  perspective = perspective_col,
  modeltype   = modeltype,
  is_hybrid   = is_hybrid
) %>%
  # Domain muss vorhanden sein
  filter(!is.na(DomainN) & nzchar(trimws(DomainN))) %>%
  # Outcome-Filter: Youth (social) functioning
  mutate(
    Outcome_l = tolower(trimws(ifelse(is.na(Outcome), "", Outcome))),
    flag_outcome = grepl("youth", Outcome_l) |
              grepl("social", Outcome_l) |
              grepl("function", Outcome_l)
  ) %>%
  filter(flag_outcome) %>%
  # Zeitlabels
  mutate(
    time_l = tolower(trimws(ifelse(is.na(TimeRaw), "", TimeRaw))),
    time_label = case_when(
      grepl("^post$", time_l)                    ~ "Post effects",
      grepl("follow", time_l) | grepl("long", time_l)   ~ "Follow-up effects",
      TRUE ~ NA_character_
    )
  ) %>%
  filter(!is.na(time_label)) %>%
```

Authors: Karolina Foremnik, Gaby Sroczynski, Jan Stratil, Marjan Arvandi, Anja Neumann, Barbara Buchberger
Medical Faculty, University of Duisburg-Essen, Germany
Corresponding author (KF)
E-Mail: karolina.foremnik@uni-due.de

```
 # gültige Zahlen
 filter(!is.na(g), !is.na(n1), !is.na(n2),
     is.finite(g), is.finite(n1), is.finite(n2),
     n1 > 1, n2 > 1, (n1 + n2 - 2) > 0) %>%
 # Varianz / SE + Säuberung
 mutate(
  v_g  = (n1 + n2)/(n1*n2) + (g^2)/(2*(n1 + n2 - 2)),
  sei  = sqrt(v_g),
  domain_clean     = abbr_domain(DomainN),
  perspective_clean = abbr_persp(perspective)
 ) %>%
 filter(is.finite(sei) & sei > 0)


if (nrow(df0) == 0) stop("Keine Daten nach Filter.")


# Split Stand-alone vs. Hybrid
df_standalone <- df0 %>% filter(!is_hybrid)
df_hybrid    <- df0 %>% filter(is_hybrid)


if (nrow(df_standalone) > 0)
 df_standalone$time_label <- factor(df_standalone$time_label,
                  levels = c("Post effects","Follow-up effects"))
if (nrow(df_hybrid) > 0)
 df_hybrid$time_label    <- factor(df_hybrid$time_label,
                  levels = c("Post effects","Follow-up effects"))


# ---------------------------- Meta-Analysen ---------------------------------
```

Article title: Efficacy of home treatment and inpatient treatment for children and adolescents in psychiatric crisis: A systematic review and meta-analysis

Journal: European Child & Adolescent Psychiatry

Authors: Karolina Foremnik, Gaby Sroczynski, Jan Stratil, Marjan Arvandi, Anja Neumann, Barbara Buchberger
Medical Faculty, University of Duisburg-Essen, Germany
Corresponding author (KF)
E-Mail: karolina.foremnik@uni-due.de

```
# Non-hybrid (gepoolt)

if (nrow(df_standalone) > 0) {

  m_standalone <- metagen(

    TE   = df_standalone$g,

    seTE = df_standalone$sei,

    studlab = df_standalone$studyid,

    data    = df_standalone,

    sm      = "SMD",

    method.tau      = "PM",

    method.random.ci = "HK",

    adhoc.hakn.ci    = "IQWiG6",

    random  = TRUE,

    common  = FALSE,

    subgroup = df_standalone$time_label,

    prediction.subgroup = TRUE,

    keepdata = TRUE

  )

} else {

  m_standalone <- NULL

}


# Hybrid (Single-Study, kein Pooling)

if (nrow(df_hybrid) > 0) {

  m_hybrid <- metagen(

    TE   = df_hybrid$g,

    seTE = df_hybrid$sei,

    studlab = paste0(df_hybrid$studyid, " [Single-study]"),
```

Authors: Karolina Foremnik, Gaby Sroczynski, Jan Stratil, Marjan Arvandi, Anja Neumann, Barbara Buchberger
Medical Faculty, University of Duisburg-Essen, Germany
Corresponding author (KF)
E-Mail: karolina.foremnik@uni-due.de

```
    data    = df_hybrid,
    sm      = "SMD",
    random  = FALSE,
    common  = FALSE,
    subgroup = df_hybrid$time_label,
    keepdata = TRUE
  )
} else {
  m_hybrid <- NULL
}

# ---------------------------- Forest Plot 174 x 200 mm ----------------------
pdf(outfile, width = 174/25.4, height = 200/25.4)

# Zwei Panels: oben non-hybrid (gepoolt), unten Hybrid
layout(matrix(c(1,2), nrow = 2, byrow = TRUE), heights = c(0.65, 0.35))

## -------- Panel 1: Non-hybrid (gepoolt) -----------------------------------
par(mai = c(1.0, 1.2, 0.8, 0.2))  # unten / links / oben / rechts

if (!is.null(m_standalone)) {
  forest(
    m_standalone,
    overall          = FALSE,
    overall.hetstat    = FALSE,
    prediction        = TRUE,
    prediction.subgroup = TRUE,
```

Authors: Karolina Foremnik, Gaby Sroczynski, Jan Stratil, Marjan Arvandi, Anja Neumann, Barbara Buchberger
Medical Faculty, University of Duisburg-Essen, Germany
Corresponding author (KF)
E-Mail: karolina.foremnik@uni-due.de

```
  print.byvar        = TRUE,
  print.subgroup.labels = TRUE,
  bylab              = "",

  leftcols  = c("studlab", "domain_clean", "perspective_clean"),
  leftlabs  = c("Study",  "Dom",        "Persp"),
  rightcols = c("effect", "ci", "w.random"),
  rightlabs = c("Hedges' g", "95% CI", "W"),

  print.I2   = TRUE,
  print.tau2 = TRUE,
  print.Q    = TRUE,

  fontsize       = 8,
  spacing        = 1.35,
  squaresize      = 0.6,
  col.diamond     = "black",
  col.diamond.lines= "black",
  colgap.forest   = "1.0mm",
  fs.hetstat      = 7,
  fs.test.subgroup = 7,

  xlim = c(-1.4, 1.4),
  xlab = "Hedges' g (change score)",
  main = "Youth (social) functioning — Non-hybrid (pooled)\nPost & Follow-up"
)
```

Authors: Karolina Foremnik, Gaby Sroczynski, Jan Stratil, Marjan Arvandi, Anja Neumann, Barbara Buchberger
Medical Faculty, University of Duisburg-Essen, Germany
Corresponding author (KF)
E-Mail: karolina.foremnik@uni-due.de

```
} else {
  plot.new()
  title("Keine non-hybrid Studien nach Filter vorhanden")
}


## -------- Panel 2: Hybrid (Single-Study, narrativ) -------------------------
par(mai = c(1.0, 1.2, 0.4, 0.2))


if (!is.null(m_hybrid)) {
  forest(
    m_hybrid,
    overall          = FALSE,
    overall.hetstat    = FALSE,
    prediction        = FALSE,
    prediction.subgroup = FALSE,


    print.byvar         = TRUE,
    print.subgroup.labels = TRUE,
    bylab             = "",


    leftcols  = c("studlab", "domain_clean", "perspective_clean"),
    leftlabs  = c("Study",  "Dom",        "Persp"),
    rightcols = c("effect", "ci"),
    rightlabs = c("Hedges' g", "95% CI"),


    print.I2   = FALSE,
    print.tau2 = FALSE,
```

Authors: Karolina Foremnik, Gaby Sroczynski, Jan Stratil, Marjan Arvandi, Anja Neumann, Barbara Buchberger
Medical Faculty, University of Duisburg-Essen, Germany
Corresponding author (KF)
E-Mail: karolina.foremnik@uni-due.de

```
    print.Q    = FALSE,


    fontsize        = 8,
    spacing         = 1.35,
    squaresize      = 0.6,
    col.diamond     = "black",
    col.diamond.lines= "black",
    colgap.forest   = "1.0mm",


    xlim = c(-1.4, 1.4),
    xlab = "Hedges' g (change score)",
    main = "Youth (social) functioning — HYBRID (Single-study, narrative)\nPost & Follow-up"
  )
} else {
  plot.new()
  title("Keine HYBRID-Zeilen (Model type = 'hybrid') vorhanden")
}


dev.off()


cat("\nFertig. Forest Plot gespeichert unter:\n", outfile, "\n")


# ---------------------------- optionale Konsolen-Summaries -------------------
if (!is.null(m_standalone)) {
  cat("\n--- Zusammenfassung NON-HYBRID (gepoolt nach Zeit) ---\n")
  print(summary(m_standalone))
```

Authors: Karolina Foremnik, Gaby Sroczynski, Jan Stratil, Marjan Arvandi, Anja Neumann, Barbara Buchberger
Medical Faculty, University of Duisburg-Essen, Germany
Corresponding author (KF)
E-Mail: karolina.foremnik@uni-due.de

```
}

if (!is.null(m_hybrid)) {

  cat("\n--- Übersicht HYBRID (Single-Study, narrativ) ---\n")

  hyb_tab <- data.frame(

    Study   = m_hybrid$studlab,

    Section = m_hybrid$byvar,

    TE      = m_hybrid$TE,

    seTE    = m_hybrid$seTE,

    CI_lower= m_hybrid$lower,

    CI_upper= m_hybrid$upper

  )

  print(hyb_tab, row.names = FALSE)

}
```

Article title: Efficacy of home treatment and inpatient treatment for children and adolescents in psychiatric crisis: A systematic review and meta-analysis
Journal: European Child & Adolescent Psychiatry
Authors: Karolina Foremnik, Gaby Sroczynski, Jan Stratil, Marjan Arvandi, Anja Neumann, Barbara Buchberger
Medical Faculty, University of Duisburg-Essen, Germany
Corresponding author (KF)
E-Mail: karolina.foremnik@uni-due.de

c) Overall Psychopathology- sequential

```
# ========= Psychopathology — Post vs Follow-up (HYBRID, Symptom domains, no pooling) =========


suppressPackageStartupMessages({
  library(readxl)
  library(meta)
  library(dplyr)
  library(grid)
  library(rlang)
  library(stringr)
  library(tibble)
})


options(stringsAsFactors = FALSE)


# ---------------- Pfade ----------------
infile  <- "/Users/karolinaforemnik/Desktop/Promotion/A_Datenextraktion und synthes/Mappe16.xlsx"
insheet <- "meta data"


outfile <- "/Users/karolinaforemnik/Desktop/Promotion/A_Datenextraktion und synthes/Forest_PSY_Post_FU_SYMPTOMDOMAINS_HYBRID_timeLabel_NOPOOL.pdf"


# ---------------- Helpers ---------------
numify <- function(x) {
  if (is.null(x)) return(NA_real_)
  x_chr <- gsub(",", ".", as.character(x), fixed = TRUE)
```

Authors: Karolina Foremnik, Gaby Sroczynski, Jan Stratil, Marjan Arvandi, Anja Neumann, Barbara Buchberger
Medical Faculty, University of Duisburg-Essen, Germany
Corresponding author (KF)
E-Mail: karolina.foremnik@uni-due.de

```
  suppressWarnings(as.numeric(x_chr))
}

letter_to_index <- function(s) {
  s <- toupper(gsub("[^A-Z]", "", s))
  chars <- strsplit(s, "")[[1]]
  idx <- 0L
  for (ch in chars) idx <- idx * 26L + match(ch, LETTERS)
  idx
}

col_by_letter <- function(df, letter) {
  i <- letter_to_index(letter)
  if (!is.na(i) && i >= 1L && i <= ncol(df)) df[[i]] else rep(NA, nrow(df))
}

safe_chr_col <- function(df, candidates) {
  for (nm in candidates) {
    if (nm %in% names(df)) return(as.character(df[[nm]]))
  }
  rep(NA_character_, nrow(df))
}

# ---------------- Daten laden -----------
raw <- read_excel(infile, sheet = insheet)

# Model type (Spalte C)
```

Authors: Karolina Foremnik, Gaby Sroczynski, Jan Stratil, Marjan Arvandi, Anja Neumann, Barbara Buchberger
Medical Faculty, University of Duisburg-Essen, Germany
Corresponding author (KF)
E-Mail: karolina.foremnik@uni-due.de

```
modeltype_raw <- col_by_letter(raw, "C")

modeltype    <- tolower(trimws(as.character(modeltype_raw)))

is_hybrid    <- !is.na(modeltype) & modeltype == "hybrid"


# Studienlabel aus Spalte A

lab_A   <- suppressWarnings(as.character(col_by_letter(raw, "A")))

studylab <- ifelse(!is.na(lab_A) & nzchar(lab_A),

           lab_A,

           paste0("Study ", seq_len(nrow(raw))))


# Outcome (Spalte D)

Outcome_raw <- col_by_letter(raw, "D")


# Symptom domains

Symptom_raw <- safe_chr_col(

  raw,

  c("Symptom domains","symptom domains",

    "Symptom_domains","symptom_domains",

    "Symptom domain","symptom domain")

)


# Time Point/ Interval

Time_raw <- safe_chr_col(

  raw,

  c("Time Point/ Interval","Time point/ interval",

    "time point/ interval","Time Point / Interval","time point / interval")

)
```

Authors: Karolina Foremnik, Gaby Sroczynski, Jan Stratil, Marjan Arvandi, Anja Neumann, Barbara Buchberger
Medical Faculty, University of Duisburg-Essen, Germany
Corresponding author (KF)
E-Mail: karolina.foremnik@uni-due.de

```
# Hedges g (Spalte AJ)

g_raw <- col_by_letter(raw, "AJ")


# Gruppengrößen (pre)

N_IG_pre <- if ("N_IG (pre)" %in% names(raw))

  numify(raw[["N_IG (pre)"]]) else numify(col_by_letter(raw, "V"))

N_CG_pre <- if ("N_CG (pre)" %in% names(raw))

  numify(raw[["N_CG (pre)"]]) else numify(col_by_letter(raw, "W"))


perspective_col <- safe_chr_col(raw, c("Perspective","perspective"))


# ---------------- Basisdatensatz --------

base_dat <- tibble(

  studyid     = studylab,

  Outcome     = as.character(Outcome_raw),

  SymptomDom  = as.character(Symptom_raw),

  TimeRaw     = as.character(Time_raw),

  g           = numify(g_raw),

  n1          = N_IG_pre,

  n2          = N_CG_pre,

  perspective = perspective_col,

  modeltype   = modeltype,

  is_hybrid   = is_hybrid

) %>%

  # Psychopathology filtern

  mutate(
```

Authors: Karolina Foremnik, Gaby Sroczynski, Jan Stratil, Marjan Arvandi, Anja Neumann, Barbara Buchberger
Medical Faculty, University of Duisburg-Essen, Germany
Corresponding author (KF)
E-Mail: karolina.foremnik@uni-due.de

```
  Outcome_l   = tolower(trimws(ifelse(is.na(Outcome), "", Outcome))),
  flag_outcome = grepl("psychopath", Outcome_l)
) %>%
filter(flag_outcome) %>%
# Symptom domains bereinigen
mutate(
  DomainN_trim = trimws(ifelse(is.na(SymptomDom), "", SymptomDom))
) %>%
filter(nzchar(DomainN_trim)) %>%
# ---------- TIME POINT: Post vs Follow-up ----------
mutate(
  Time_l = tolower(trimws(ifelse(is.na(TimeRaw), "", TimeRaw))),
  section = case_when(
    grepl("long\\s*term", Time_l) ~ "Follow-up",   # long term = Follow-up
    grepl("post", Time_l)        ~ "Post",       # post, post-discharge etc.
    TRUE                        ~ NA_character_
  )
) %>%
filter(!is.na(section)) %>%
# ---------- Effektgrößen ----------
filter(is.finite(g), is.finite(n1), is.finite(n2),
       n1 > 1, n2 > 1, (n1 + n2 - 2) > 0) %>%
mutate(
  v_g  = (n1 + n2)/(n1*n2) + (g^2)/(2*(n1 + n2 - 2)),
  sei  = sqrt(v_g),


  # Perspective bereinigen → Persp
```

Authors: Karolina Foremnik, Gaby Sroczynski, Jan Stratil, Marjan Arvandi, Anja Neumann, Barbara Buchberger
Medical Faculty, University of Duisburg-Essen, Germany
Corresponding author (KF)
E-Mail: karolina.foremnik@uni-due.de

```
    Persp = tolower(trimws(perspective)),
    Persp = dplyr::recode(
      Persp,
      "clinician-rated" = "clin",
      "self-rated"      = "self",
      "parent-rated"    = "par",
      .default          = Persp
    ),
    Persp = ifelse(is.na(Persp) | !nzchar(Persp), "n/a", Persp),

    # Domain bereinigen → Dom (inkl. general→gen)
    Dom = tolower(trimws(DomainN_trim)),
    Dom = dplyr::recode(
      Dom,
      "general" = "gen",
      .default  = Dom
    )
  ) %>%
  filter(is.finite(sei) & sei > 0)

# -------- nur HYBRID --------------------
dat_main <- base_dat %>%
  filter(is_hybrid)

if (nrow(dat_main) == 0) {
  pdf(outfile, width = 174/25.4, height = 234/25.4)
  plot.new()
```

Authors: Karolina Foremnik, Gaby Sroczynski, Jan Stratil, Marjan Arvandi, Anja Neumann, Barbara Buchberger
Medical Faculty, University of Duisburg-Essen, Germany
Corresponding author (KF)
E-Mail: karolina.foremnik@uni-due.de

```
  title("Keine HYBRID-Studien nach Filter vorhanden")
  dev.off()
  stop("Keine HYBRID-Daten nach Filter gefunden.")
}

# Subgruppen-Reihenfolge
dat_main$section <- factor(dat_main$section, levels = c("Post", "Follow-up"))

# Sortierung im Plot
dat_main <- dat_main %>%
  arrange(section, Dom, studyid)

# -------- Meta-Objekt: kein Pooling --------
m_main <- metagen(
  TE      = dat_main$g,
  seTE    = dat_main$sei,
  studlab  = dat_main$studyid,
  data    = dat_main,
  sm      = "SMD",
  random   = FALSE,
  common   = FALSE,
  byvar    = section,   # 2 Subgruppen in einem Plot
  keepdata = TRUE
)

m_main$Dom   <- dat_main$Dom
m_main$Persp <- dat_main$Persp
```

Article title: Efficacy of home treatment and inpatient treatment for children and adolescents in psychiatric crisis: A systematic review and meta-analysis
Journal: European Child & Adolescent Psychiatry
Authors: Karolina Foremnik, Gaby Sroczynski, Jan Stratil, Marjan Arvandi, Anja Neumann, Barbara Buchberger
Medical Faculty, University of Duisburg-Essen, Germany
Corresponding author (KF)
E-Mail: karolina.foremnik@uni-due.de

```
# ---------------- Dynamische Höhe für PDF ----------------
# Anzahl der Zeilen (Studien) + Anzahl Subgruppen-Zeilen
n_rows <- nrow(dat_main) + length(unique(dat_main$section))

# Höhe pro Zeile in mm (etwas größer, damit X-Achse nicht abgeschnitten wird)
row_height_mm <- 12.0

# Ober-/Unterrand in mm
margin_top_bottom_mm <- 20

# Gesamthöhe in mm und dann in inch
total_height_mm <- n_rows * row_height_mm + margin_top_bottom_mm
total_height_in <- total_height_mm / 25.4
total_width_in  <- 140 / 25.4   # Breite wie gehabt

pdf(outfile, width = total_width_in, height = total_height_in)
# größerer unterer Rand, damit Achsenbeschriftung + X-Achse sichtbar bleiben
par(mai = c(1.0, 1.6, 0.9, 0.2))

# ---------------- Forest Plot -----------
forest(
  m_main,
  print.byvar      = TRUE,
  bylab          = "Time point",

  overall         = FALSE,
```

Authors: Karolina Foremnik, Gaby Sroczynski, Jan Stratil, Marjan Arvandi, Anja Neumann, Barbara Buchberger
Medical Faculty, University of Duisburg-Essen, Germany
Corresponding author (KF)
E-Mail: karolina.foremnik@uni-due.de

```
  overall.hetstat    = FALSE,
  prediction         = FALSE,
  prediction.subgroup = FALSE,

  leftcols  = c("studlab","Dom","Persp"),
  leftlabs  = c("Study","Dom","Persp"),

  rightcols = c("effect","ci"),
  rightlabs = c("Hedges' g","95% CI"),

  print.I2   = FALSE,
  print.tau2 = FALSE,
  print.Q    = FALSE,

  fontsize     = 8,
  spacing      = 1.35,
  squaresize   = 0.6,
  col.diamond   = "black",
  col.diamond.lines = "black",
  colgap.forest = "1.0mm",

  xlim        = c(-2, 2),
  xlab        = "Hedges' g (Change Score)",
  main        = "Psychopathology — Post vs Follow-up (HYBRID, Symptom domains, no
pooling)"
)
```

Authors: Karolina Foremnik, Gaby Sroczynski, Jan Stratil, Marjan Arvandi, Anja Neumann, Barbara Buchberger
Medical Faculty, University of Duisburg-Essen, Germany
Corresponding author (KF)
E-Mail: karolina.foremnik@uni-due.de

```
dev.off()



cat("\nFertig. Forest-Plot gespeichert:\n", outfile, "\n")
```

Authors: Karolina Foremnik, Gaby Sroczynski, Jan Stratil, Marjan Arvandi, Anja Neumann, Barbara Buchberger
Medical Faculty, University of Duisburg-Essen, Germany
Corresponding author (KF)
E-Mail: karolina.foremnik@uni-due.de

d) Overall Psychosocial functioning- sequential

```
# ========= Youth Social Functioning — Post vs Follow-up (HYBRID, no pooling) =========

suppressPackageStartupMessages({
  library(readxl)
  library(meta)
  library(dplyr)
  library(grid)
  library(rlang)
  library(stringr)
  library(tibble)
})

options(stringsAsFactors = FALSE)

# ---------------- Pfade ----------------
infile  <- "/Users/karolinaforemnik/Desktop/Promotion/A_Datenextraktion und synthes/Mappe16.xlsx"
insheet <- "meta data"

outfile <- "/Users/karolinaforemnik/Desktop/Promotion/A_Datenextraktion und synthes/Forest_YOUTH_Post_FU_HYBRID_NOPOOL.pdf"

# ---------------- Helpers ---------------
numify <- function(x) {
  if (is.null(x)) return(NA_real_)
```

Authors: Karolina Foremnik, Gaby Sroczynski, Jan Stratil, Marjan Arvandi, Anja Neumann, Barbara Buchberger
Medical Faculty, University of Duisburg-Essen, Germany
Corresponding author (KF)
E-Mail: karolina.foremnik@uni-due.de

```
  x_chr <- gsub(",", ".", as.character(x), fixed = TRUE)
  suppressWarnings(as.numeric(x_chr))
}

letter_to_index <- function(s) {
  s <- toupper(gsub("[^A-Z]", "", s))
  chars <- strsplit(s, "")[[1]]
  idx <- 0L
  for (ch in chars) idx <- idx * 26L + match(ch, LETTERS)
  idx
}

col_by_letter <- function(df, letter) {
  i <- letter_to_index(letter)
  if (!is.na(i) && i >= 1L && i <= ncol(df)) df[[i]] else rep(NA, nrow(df))
}

safe_chr_col <- function(df, candidates) {
  for (nm in candidates) {
    if (nm %in% names(df)) return(as.character(df[[nm]]))
  }
  rep(NA_character_, nrow(df))
}

# ---------------- Daten laden -----------
raw <- read_excel(infile, sheet = insheet)
```

Article title: Efficacy of home treatment and inpatient treatment for children and adolescents in psychiatric crisis: A systematic review and meta-analysis
Journal: European Child & Adolescent Psychiatry
Authors: Karolina Foremnik, Gaby Sroczynski, Jan Stratil, Marjan Arvandi, Anja Neumann, Barbara Buchberger
Medical Faculty, University of Duisburg-Essen, Germany
Corresponding author (KF)
E-Mail: karolina.foremnik@uni-due.de

```
# Model type

modeltype_raw <- col_by_letter(raw, "C")

modeltype    <- tolower(trimws(as.character(modeltype_raw)))

is_hybrid    <- !is.na(modeltype) & modeltype == "hybrid"


# Studienlabel

lab_A   <- suppressWarnings(as.character(col_by_letter(raw, "A")))

studylab <- ifelse(!is.na(lab_A) & nzchar(lab_A),

           lab_A,

           paste0("Study ", seq_len(nrow(raw))))


# Outcome (D)

Outcome_raw <- col_by_letter(raw, "D")


# Domain (Symptom / Functioning)

Domain_raw <- safe_chr_col(

  raw,

  c("Symptom domains","symptom domains",

   "Symptom_domains","symptom_domains",

   "Symptom domain","symptom domain",

   "Functioning Domain","functioning domain","Domain","domain")

)


# Time Point/ Interval

Time_raw <- safe_chr_col(

  raw,

  c("Time Point/ Interval","Time point/ interval","time point/ interval",
```

Authors: Karolina Foremnik, Gaby Sroczynski, Jan Stratil, Marjan Arvandi, Anja Neumann, Barbara Buchberger
Medical Faculty, University of Duisburg-Essen, Germany
Corresponding author (KF)
E-Mail: karolina.foremnik@uni-due.de

```
   "Time Point / Interval","time point / interval")
)


# Hedges g (AJ)
g_raw <- col_by_letter(raw, "AJ")


# Gruppengrößen (pre)
N_IG_pre <- if ("N_IG (pre)" %in% names(raw))
  numify(raw[["N_IG (pre)"]]) else numify(col_by_letter(raw, "V"))
N_CG_pre <- if ("N_CG (pre)" %in% names(raw))
  numify(raw[["N_CG (pre)"]]) else numify(col_by_letter(raw, "W"))


perspective_col <- safe_chr_col(raw, c("Perspective","perspective"))


# ---------------- Basisdatensatz --------
base_dat <- tibble(
  studyid     = studylab,
  Outcome     = as.character(Outcome_raw),
  DomainN     = as.character(Domain_raw),
  TimeRaw     = as.character(Time_raw),
  g           = numify(g_raw),
  n1          = N_IG_pre,
  n2          = N_CG_pre,
  perspective = perspective_col,
  modeltype   = modeltype,
  is_hybrid   = is_hybrid
) %>%
```

Authors: Karolina Foremnik, Gaby Sroczynski, Jan Stratil, Marjan Arvandi, Anja Neumann, Barbara Buchberger
Medical Faculty, University of Duisburg-Essen, Germany
Corresponding author (KF)
E-Mail: karolina.foremnik@uni-due.de

```
# ---- Filter für Youth Social Functioning ----
mutate(
  Outcome_l = tolower(trimws(ifelse(is.na(Outcome), "", Outcome))),
  flag_outcome = grepl("youth", Outcome_l) & grepl("function", Outcome_l)
) %>%
filter(flag_outcome) %>%
# Domain = Dom
mutate(
  DomainN_trim = trimws(ifelse(is.na(DomainN), "", DomainN))
) %>%
filter(nzchar(DomainN_trim)) %>%
# ---- Post vs Follow-up ----
mutate(
  Time_l = tolower(trimws(ifelse(is.na(TimeRaw), "", TimeRaw))),
  section = case_when(
    grepl("long\\s*term", Time_l) ~ "Follow-up",
    grepl("post", Time_l)        ~ "Post",
    TRUE                         ~ NA_character_
  )
) %>%
filter(!is.na(section)) %>%
# ---- Effektgrößen ----
filter(is.finite(g), is.finite(n1), is.finite(n2),
       n1 > 1, n2 > 1, (n1 + n2 - 2) > 0) %>%
mutate(
  v_g  = (n1 + n2)/(n1*n2) + (g^2)/(2*(n1 + n2 - 2)),
  sei  = sqrt(v_g),
```

Authors: Karolina Foremnik, Gaby Sroczynski, Jan Stratil, Marjan Arvandi, Anja Neumann, Barbara Buchberger
Medical Faculty, University of Duisburg-Essen, Germany
Corresponding author (KF)
E-Mail: karolina.foremnik@uni-due.de

```
    Persp = tolower(trimws(perspective)),
    Persp = dplyr::recode(
      Persp,
      "clinician-rated" = "clin",
      "self-rated"      = "self",
      "parent-rated"    = "par",
      .default          = Persp
    ),
    Persp = ifelse(is.na(Persp) | !nzchar(Persp), "n/a", Persp),

    Dom = tolower(trimws(DomainN_trim)),
    Dom = dplyr::recode(
      Dom,
      "general"             = "gen",
      "overall"             = "overall f.",
      "overall functioning" = "overall f.",
      "functioning overall" = "overall f.",
      "overall_func"        = "overall f.",
      .default              = Dom
    )
  ) %>%
  filter(is.finite(sei) & sei > 0)

# ---- Nur HYBRID ----
dat_main <- base_dat %>% filter(is_hybrid)
```

Article title: Efficacy of home treatment and inpatient treatment for children and adolescents in psychiatric crisis: A systematic review and meta-analysis
Journal: European Child & Adolescent Psychiatry
Authors: Karolina Foremnik, Gaby Sroczynski, Jan Stratil, Marjan Arvandi, Anja Neumann, Barbara Buchberger
Medical Faculty, University of Duisburg-Essen, Germany
Corresponding author (KF)
E-Mail: karolina.foremnik@uni-due.de

```
if (nrow(dat_main) == 0) {
  pdf(outfile, width = 140/25.4, height = 200/25.4)
  plot.new()
  title("Keine HYBRID-Daten für Youth Social Functioning gefunden")
  dev.off()
  stop("Keine HYBRID-Daten gefunden.")
}

dat_main$section <- factor(dat_main$section, levels = c("Post","Follow-up"))

dat_main <- dat_main %>% arrange(section, Dom, studyid)

# ---- Meta (kein Pooling) ----
m_main <- metagen(
  TE      = dat_main$g,
  seTE    = dat_main$sei,
  studlab  = dat_main$studyid,
  data    = dat_main,
  sm      = "SMD",     # intern SMD, in der Beschriftung Hedges' g
  random   = FALSE,
  common   = FALSE,
  byvar    = section,
  keepdata = TRUE
)

m_main$Dom   <- dat_main$Dom
m_main$Persp <- dat_main$Persp
```

Article title: Efficacy of home treatment and inpatient treatment for children and adolescents in psychiatric crisis: A systematic review and meta-analysis
Journal: European Child & Adolescent Psychiatry
Authors: Karolina Foremnik, Gaby Sroczynski, Jan Stratil, Marjan Arvandi, Anja Neumann, Barbara Buchberger
Medical Faculty, University of Duisburg-Essen, Germany
Corresponding author (KF)
E-Mail: karolina.foremnik@uni-due.de

```
# ---------------- Dynamische Höhe ----------------
n_rows <- nrow(dat_main) + length(unique(dat_main$section))
row_height_mm <- 12.0
margin_top_bottom_mm <- 20

total_height_mm <- n_rows * row_height_mm + margin_top_bottom_mm
total_height_in <- total_height_mm / 25.4
total_width_in  <- 140 / 25.4

pdf(outfile, width = total_width_in, height = total_height_in)
par(mai = c(1.0, 1.6, 0.9, 0.2))

# ---------------- Forest Plot ----------------
forest(
  m_main,
  print.byvar      = TRUE,
  bylab            = "Time point",

  overall          = FALSE,
  overall.hetstat    = FALSE,

  leftcols  = c("studlab","Dom","Persp"),
  leftlabs  = c("Study","Dom","Persp"),

  rightcols = c("effect","ci"),
  rightlabs = c("Hedges' g","95% CI"),
```

Authors: Karolina Foremnik, Gaby Sroczynski, Jan Stratil, Marjan Arvandi, Anja Neumann, Barbara Buchberger
Medical Faculty, University of Duisburg-Essen, Germany
Corresponding author (KF)
E-Mail: karolina.foremnik@uni-due.de

```
  fontsize     = 8,
  spacing      = 1.35,
  squaresize   = 0.6,
  col.diamond   = "black",
  col.diamond.lines = "black",

  xlim        = c(-2, 2),
  xlab        = "Hedges' g (Change Score)",
  main        = "Youth Social Functioning — Post vs Follow-up (HYBRID, no pooling)"
)

dev.off()

cat("\nFertig. Forest-Plot gespeichert:\n", outfile, "\n")
```

Article title: Efficacy of home treatment and inpatient treatment for children and adolescents in psychiatric crisis: A systematic review and meta-analysis
Journal: European Child & Adolescent Psychiatry
Authors: Karolina Foremnik, Gaby Sroczynski, Jan Stratil, Marjan Arvandi, Anja Neumann, Barbara Buchberger
Medical Faculty, University of Duisburg-Essen, Germany
Corresponding author (KF)
E-Mail: karolina.foremnik@uni-due.de

Family functioning

```
# ---------------------------- Pakete ----------------------------------------

suppressPackageStartupMessages({

  library(readxl)

  library(meta)

  library(dplyr)

  library(forcats)

  library(grid)

  library(rlang)

  library(stringr)

  library(tibble)

})


options(stringsAsFactors = FALSE)


# ---------------------------- Pfade -----------------------------------------

infile  <- "/Users/karolinaforemnik/Desktop/Promotion/A_Datenextraktion und synthes/Mappe16.xlsx"

insheet <- "meta data"

outfile <- "/Users/karolinaforemnik/Desktop/Promotion/A_Datenextraktion und synthes/Forest_FamilyFunctioning_POST_FU_NoPooling_SortedDomains.pdf"


# ---------------------------- Helpers ---------------------------------------

numify <- function(x) {

  if (is.null(x)) return(NA_real_)

  x_chr <- as.character(x)

  x_chr <- gsub(",", ".", x_chr, fixed = TRUE)
```

Authors: Karolina Foremnik, Gaby Sroczynski, Jan Stratil, Marjan Arvandi, Anja Neumann, Barbara Buchberger
Medical Faculty, University of Duisburg-Essen, Germany
Corresponding author (KF)
E-Mail: karolina.foremnik@uni-due.de

```
  suppressWarnings(as.numeric(x_chr))
}

letter_to_index <- function(s) {
  s <- toupper(gsub("[^A-Z]", "", s))
  chars <- strsplit(s, "")[[1]]
  idx <- 0L
  for (ch in chars) idx <- idx * 26L + match(ch, LETTERS)
  idx
}

col_by_letter <- function(df, letter) {
  i <- letter_to_index(letter)
  if (!is.na(i) && i >= 1L && i <= ncol(df)) df[[i]] else rep(NA, nrow(df))
}

safe_chr_col <- function(df, candidates) {
  for (nm in candidates) if (!is.null(df[[nm]])) return(as.character(df[[nm]]))
  rep(NA_character_, nrow(df))
}

# ---------------------------- Daten laden -----------------------------------
raw <- read_excel(infile, sheet = insheet)

# Studienlabel (A + C)
lab_A <- suppressWarnings(as.character(col_by_letter(raw, "A")))
lab_C <- suppressWarnings(as.character(col_by_letter(raw, "C")))
```

Article title: Efficacy of home treatment and inpatient treatment for children and adolescents in psychiatric crisis: A systematic review and meta-analysis
Journal: European Child & Adolescent Psychiatry
Authors: Karolina Foremnik, Gaby Sroczynski, Jan Stratil, Marjan Arvandi, Anja Neumann, Barbara Buchberger
Medical Faculty, University of Duisburg-Essen, Germany
Corresponding author (KF)
E-Mail: karolina.foremnik@uni-due.de

```
studylab <- ifelse(
  !is.na(lab_A) & nzchar(lab_A),
  ifelse(!is.na(lab_C) & nzchar(lab_C), paste0(lab_A, " (", lab_C, ")"), lab_A),
  paste0("Study ", seq_len(nrow(raw)))
)


# "(stand alone)" entfernen
studylab <- gsub("\\(stand[- ]*alone\\)", "", studylab, ignore.case = TRUE)
studylab <- trimws(studylab)


# Kerndaten
Outcome_raw <- col_by_letter(raw, "D")
Time_raw    <- col_by_letter(raw, "P")
g_raw       <- col_by_letter(raw, "AJ")


# Stichprobe
N_IG_pre <- if ("N_IG (pre)" %in% names(raw)) numify(raw[["N_IG (pre)"]]) else
numify(col_by_letter(raw, "V"))
N_CG_pre <- if ("N_CG (pre)" %in% names(raw)) numify(raw[["N_CG (pre)"]]) else
numify(col_by_letter(raw, "W"))


# Anzeige-Spalten
perspective_col <- safe_chr_col(raw, c("Perspective","perspective"))
domain_G       <- as.character(col_by_letter(raw, "G"))


# ----------------------------- Aufbereiten/Filtern ----------------------------
dat <- tibble(
```

Article title: Efficacy of home treatment and inpatient treatment for children and adolescents in psychiatric crisis: A systematic review and meta-analysis
Journal: European Child & Adolescent Psychiatry
Authors: Karolina Foremnik, Gaby Sroczynski, Jan Stratil, Marjan Arvandi, Anja Neumann, Barbara Buchberger
Medical Faculty, University of Duisburg-Essen, Germany
Corresponding author (KF)
E-Mail: karolina.foremnik@uni-due.de

```
 studyid    = studylab,
 Outcome    = as.character(Outcome_raw),
 TimeRaw    = as.character(Time_raw),
 g          = numify(g_raw),
 n1         = N_IG_pre,
 n2         = N_CG_pre,
 perspective = perspective_col,
 domain_G   = domain_G
) %>%
 mutate(
  Outcome_l   = tolower(trimws(ifelse(is.na(Outcome), "", Outcome))),
  flag_outcome = grepl("family", Outcome_l) & grepl("function", Outcome_l)
 ) %>%
 filter(flag_outcome) %>%
 mutate(
  time_l = tolower(trimws(ifelse(is.na(TimeRaw), "", TimeRaw))),
  section = case_when(
   grepl("^post$", time_l) ~ "Post effects",
   grepl("follow", time_l) | grepl("long", time_l) ~ "Follow-up effects",
   TRUE ~ NA_character_
  )
 ) %>%
 filter(!is.na(section)) %>%
 filter(
  is.finite(g), is.finite(n1), is.finite(n2),
  n1 > 1, n2 > 1, (n1 + n2 - 2) > 0
 ) %>%
```

Authors: Karolina Foremnik, Gaby Sroczynski, Jan Stratil, Marjan Arvandi, Anja Neumann, Barbara Buchberger
Medical Faculty, University of Duisburg-Essen, Germany
Corresponding author (KF)
E-Mail: karolina.foremnik@uni-due.de

```
 mutate(
  v_g  = (n1 + n2)/(n1*n2) + (g^2)/(2*(n1 + n2 - 2)),
  sei  = sqrt(v_g),

  # Perspektiven-Abkürzung
  perspective_clean = tolower(trimws(perspective)),
  perspective_clean = dplyr::recode(
   perspective_clean,
   "clinician-rated" = "clin",
   "self-rated"      = "self",
   "parent-rated"    = "par",
   .default = ifelse(
    is.na(perspective_clean) | !nzchar(perspective_clean),
    "n/a",
    perspective_clean
   )
  ),

  domain_clean = ifelse(
   is.na(domain_G) | !nzchar(trimws(domain_G)),
   "n/a",
   tolower(trimws(domain_G))
  )
 ) %>%
 filter(is.finite(sei) & sei > 0)

if (nrow(dat) == 0) stop("Keine Daten nach Filter.")
```

Article title: Efficacy of home treatment and inpatient treatment for children and adolescents in psychiatric crisis: A systematic review and meta-analysis
Journal: European Child & Adolescent Psychiatry
Authors: Karolina Foremnik, Gaby Sroczynski, Jan Stratil, Marjan Arvandi, Anja Neumann, Barbara Buchberger
Medical Faculty, University of Duisburg-Essen, Germany
Corresponding author (KF)
E-Mail: karolina.foremnik@uni-due.de

```
# ---------------------------- Domänen sortieren ------------------------------

domain_order <- c(

  "cohesion", "adaptability", "communication", "control",

  "parent emotional distress", "general", "n/a"

)


dat <- dat %>%

  mutate(

    domain_clean = fct_relevel(domain_clean, domain_order, after = 0),

    section      = factor(section, levels = c("Post effects", "Follow-up effects"))

  ) %>%

  arrange(section, domain_clean, studyid)


# ---------------------------- Meta-Objekt (für Forest, kein Pooling im Plot) -

m <- metagen(

  TE   = dat$g,

  seTE = dat$sei,

  studlab = dat$studyid,

  data    = dat,

  sm      = "SMD",

  method.tau      = "PM",

  method.random.ci = "HK",

  random  = TRUE,

  common  = FALSE,

  subgroup = dat$section,

  prediction.subgroup = FALSE,
```

Authors: Karolina Foremnik, Gaby Sroczynski, Jan Stratil, Marjan Arvandi, Anja Neumann, Barbara Buchberger
Medical Faculty, University of Duisburg-Essen, Germany
Corresponding author (KF)
E-Mail: karolina.foremnik@uni-due.de

```
  keepdata = TRUE
)

# ----------------------------- Forest 174 × 234 mm ----------------------------
pdf(outfile, width = 174/25.4, height = 150/25.4)

par(mai = c(1.0, 1.2, 0.8, 0.2))

forest(
  m,
  overall            = FALSE,
  overall.hetstat    = FALSE,
  prediction         = FALSE,
  prediction.subgroup = FALSE,

  subgroup           = FALSE,
  subgroup.hetstat   = FALSE,

  print.byvar        = TRUE,
  print.subgroup.labels = TRUE,
  bylab              = "",

  leftcols  = c("studlab","domain_clean","perspective_clean"),
  leftlabs  = c("Study","Dom","Persp"),

  # *** KEIN weight mehr anzeigen ***
  rightcols = c("effect","ci"),
```

Authors: Karolina Foremnik, Gaby Sroczynski, Jan Stratil, Marjan Arvandi, Anja Neumann, Barbara Buchberger
Medical Faculty, University of Duisburg-Essen, Germany
Corresponding author (KF)
E-Mail: karolina.foremnik@uni-due.de

```
  rightlabs = c("Hedges' g","95% CI"),


  print.I2   = FALSE,
  print.tau2 = FALSE,
  print.Q    = FALSE,


  fontsize     = 8,
  spacing      = 1.35,
  squaresize   = 0.6,
  col.diamond   = "black",
  col.diamond.lines = "black",
  colgap.forest = "1.0mm",


  fs.hetstat       = 7,
  fs.test.subgroup  = 7,


  xlim        = c(-1.4, 1.4),
  xlab        = "Hedges' g (change score)",


  main        = "Family functioning — Post & Follow-up (no pooling)\nFacetten sortiert"
)


dev.off()


cat("\nFertig. Forest-Plot gespeichert:\n", outfile, "\n")
print(summary(m))
```

Article title: Efficacy of home treatment and inpatient treatment for children and adolescents in psychiatric crisis: A systematic review and meta-analysis
Journal: European Child & Adolescent Psychiatry
Authors: Karolina Foremnik, Gaby Sroczynski, Jan Stratil, Marjan Arvandi, Anja Neumann, Barbara Buchberger
Medical Faculty, University of Duisburg-Essen, Germany
Corresponding author (KF)
E-Mail: karolina.foremnik@uni-due.de

Readmissions

```
## ====================== META-ANALYSIS: Readmission, Double-Column Export ======================

if (!requireNamespace("meta", quietly = TRUE)) install.packages("meta")

suppressPackageStartupMessages({
  library(meta)
  library(grid)
})

options(stringsAsFactors = FALSE)

## 0) Output-Dateien explizit für READMISSION definieren ----------------------
outfile_pdf <- "/Users/karolinaforemnik/Desktop/Promotion/A_Datenextraktion und synthes/Forest_Readmission_ModelType_doublecolumn.pdf"
outfile_jpg <- "/Users/karolinaforemnik/Desktop/Promotion/A_Datenextraktion und synthes/Forest_Readmission_ModelType_doublecolumn.jpg"

## 1) Daten -------------------------------------------------------------------
df <- data.frame(
  StudyID = c(
    "Boege et al. 2021",
    "Schoenwald et al. 2000",
    "Schmidt et al. 2006",
    "Ougrin et al. 2021",
    "Graf et al. 2025"
  ),
```

Authors: Karolina Foremnik, Gaby Sroczynski, Jan Stratil, Marjan Arvandi, Anja Neumann, Barbara Buchberger
Medical Faculty, University of Duisburg-Essen, Germany
Corresponding author (KF)
E-Mail: karolina.foremnik@uni-due.de

```
  N_IG = c(28, 57, 59, 53, 27),
  N_CG = c(23, 56, 30, 53, 48),
  p_IG = c(0.167, 0.280, 0.170, NA, 0.593),
  p_CG = c(0.167, 0.200, 0.130, NA, 0.417),
  E_IG_manual = c(NA, NA, NA, 11, NA),
  E_CG_manual = c(NA, NA, NA, 12, NA),
  Model = c("Hybrid", "Stand-alone", "Stand-alone", "Hybrid", "Stand-alone"),
  FollowUp_months = c(52, 3.5, 12, 6, 21),
  stringsAsFactors = FALSE
)


df$Model <- factor(df$Model, levels = c("Stand-alone", "Hybrid"))


k_total <- nrow(df)
do_pool <- k_total >= 3
k_stand <- sum(df$Model == "Stand-alone")
k_hybrid <- sum(df$Model == "Hybrid")


## 2) Events bestimmen -------------------------------------------------------
## Standardmäßig aus Prozenten rekonstruieren,
## aber für Studien mit echten Eventzahlen diese direkt verwenden.
df$E_IG <- ifelse(
  !is.na(df$E_IG_manual),
  df$E_IG_manual,
  pmax(0, pmin(df$N_IG, round(df$p_IG * df$N_IG)))
)
```

Authors: Karolina Foremnik, Gaby Sroczynski, Jan Stratil, Marjan Arvandi, Anja Neumann, Barbara Buchberger
Medical Faculty, University of Duisburg-Essen, Germany
Corresponding author (KF)
E-Mail: karolina.foremnik@uni-due.de

```
df$E_CG <- ifelse(
  !is.na(df$E_CG_manual),
  df$E_CG_manual,
  pmax(0, pmin(df$N_CG, round(df$p_CG * df$N_CG)))
)


## Kontrolle
df$p_IG_used <- df$E_IG / df$N_IG
df$p_CG_used <- df$E_CG / df$N_CG


## 3) Kontinuitätskorrektur -------------------------------------------------
add_cc <- function(E, N, cc = 0.5) {
  zf <- (E == 0) | (E == N)
  list(
    E = ifelse(zf, E + cc, E),
    N = ifelse(zf, N + 2 * cc, N)
  )
}


cc <- 0.5
adj_IG <- add_cc(df$E_IG, df$N_IG, cc)
adj_CG <- add_cc(df$E_CG, df$N_CG, cc)


EIG <- adj_IG$E
NIG <- adj_IG$N
ECG <- adj_CG$E
NCG <- adj_CG$N
```

Article title: Efficacy of home treatment and inpatient treatment for children and adolescents in psychiatric crisis: A systematic review and meta-analysis
Journal: European Child & Adolescent Psychiatry
Authors: Karolina Foremnik, Gaby Sroczynski, Jan Stratil, Marjan Arvandi, Anja Neumann, Barbara Buchberger
Medical Faculty, University of Duisburg-Essen, Germany
Corresponding author (KF)
E-Mail: karolina.foremnik@uni-due.de

```
## 4) ln(RR) & SE -----------------------------------------------------------

lnRR <- log((EIG / NIG) / (ECG / NCG))

SEln <- sqrt(1 / EIG - 1 / NIG + 1 / ECG - 1 / NCG)


## 5) Meta-Analyse -----------------------------------------------------------

res <- metagen(

  TE = lnRR,

  seTE = SEln,

  studlab = df$StudyID,

  sm = "RR",

  method.tau = "PM",

  hakn = TRUE,

  adhoc.hakn = "ci",

  comb.fixed = do_pool,

  comb.random = do_pool,

  byvar = df$Model

)


## Subgruppen-Diamant regeln

if (do_pool && !is.null(res$TE.random.w)) {

  ## Stand-alone Diamant unterdrücken wenn < 3 Studien

  if (k_stand < 3) {

    res$TE.random.w[1] <- NA

    res$seTE.random.w[1] <- NA

    res$lower.random.w[1] <- NA

    res$upper.random.w[1] <- NA
```

Authors: Karolina Foremnik, Gaby Sroczynski, Jan Stratil, Marjan Arvandi, Anja Neumann, Barbara Buchberger
Medical Faculty, University of Duisburg-Essen, Germany
Corresponding author (KF)
E-Mail: karolina.foremnik@uni-due.de

```
  }

  ## Hybrid Diamant immer unterdrücken
  res$TE.random.w[2] <- NA
  res$seTE.random.w[2] <- NA
  res$lower.random.w[2] <- NA
  res$upper.random.w[2] <- NA
}

## 6) Zusatzspalten -----------------------------------------------------------
res$FollowUp_months <- df$FollowUp_months

rightcols <- if (do_pool) c("effect", "ci", "w.random") else c("effect", "ci")
rightlabs <- if (do_pool) c("RR", "95% CI", "W") else c("RR", "95% CI")

## 7) Forest-Zeichenfunktion ---------------------------------------------------
draw_forest <- function() {
  par(mai = c(0.20, 0.50, 0.10, 0.05))  # bottom, left, top, right in inches

  forest(
    res,
    common = FALSE,
    random = do_pool,
    overall = do_pool,
    prediction = do_pool,
    print.tau2 = do_pool,
    print.I2 = do_pool,
```

Authors: Karolina Foremnik, Gaby Sroczynski, Jan Stratil, Marjan Arvandi, Anja Neumann, Barbara Buchberger
Medical Faculty, University of Duisburg-Essen, Germany
Corresponding author (KF)
E-Mail: karolina.foremnik@uni-due.de

```
    bylab = "Model type",
    backtransf = TRUE,
    leftcols = c("studlab", "FollowUp_months"),
    leftlabs = c("Study", "Follow-up (months)"),
    rightcols = rightcols,
    rightlabs = rightlabs,
    fontsize = 8,
    spacing = 0.90,
    squaresize = 0.6,
    col.diamond = "black",
    col.diamond.lines = "black",
    colgap.forest = "1.0mm",
    fs.hetstat = 7,
    xlab = "Readmission: Risk Ratio (RR) with 95% CI",
    subgroup = do_pool,
    subgroup.hetstat = if (do_pool) c(TRUE, FALSE) else FALSE,
    test.subgroup = FALSE
  )
}

## 8) Export ---------------------------------------------------------------
## PDF (Vektor) – 174 mm Breite (zweispaltig)
pdf(outfile_pdf, width = 174 / 25.4, height = 150 / 25.4)
draw_forest()
dev.off()

## JPEG – 174 mm @ 600 dpi (Springer-konform)
```

Authors: Karolina Foremnik, Gaby Sroczynski, Jan Stratil, Marjan Arvandi, Anja Neumann, Barbara Buchberger
Medical Faculty, University of Duisburg-Essen, Germany
Corresponding author (KF)
E-Mail: karolina.foremnik@uni-due.de

```
jpeg(
  outfile_jpg,
  width = 17.4,   # cm = 174 mm
  height = 10.0,  # cm
  units = "cm",
  res = 600,
  quality = 100
)
draw_forest()
dev.off()


cat(
  "\nFertig. Readmission-Forest-Plot gespeichert als:\n",
  outfile_pdf, "\n",
  outfile_jpg, "\n"
)


## 9) Meta-Analyse-Zusammenfassung ausgeben ----------------------------------
print(summary(res))


## 10) Kontrollausgabe für verwendete Eventzahlen ----------------------------
print(df[, c("StudyID", "N_IG", "E_IG", "p_IG_used", "N_CG", "E_CG", "p_CG_used")])
```
